# A second generation genetic map for rainbow trout (*Oncorhynchus mykiss*)

**DOI:** 10.1186/1471-2156-9-74

**Published:** 2008-11-19

**Authors:** Caird E Rexroad, Yniv Palti, Scott A Gahr, Roger L Vallejo

**Affiliations:** 1 USDA/ARS National Center for Cool and Cold Water Aquaculture, Leetown, West Virginia, USA

## Abstract

**Background:**

Genetic maps characterizing the inheritance patterns of traits and markers have been developed for a wide range of species and used to study questions in biomedicine, agriculture, ecology and evolutionary biology. The status of rainbow trout genetic maps has progressed significantly over the last decade due to interest in this species in aquaculture and sport fisheries, and as a model research organism for studies related to carcinogenesis, toxicology, comparative immunology, disease ecology, physiology and nutrition. We constructed a second generation genetic map for rainbow trout using microsatellite markers to facilitate the identification of quantitative trait loci for traits affecting aquaculture production efficiency and the extraction of comparative information from the genome sequences of model fish species.

**Results:**

A genetic map ordering 1124 microsatellite loci spanning a sex-averaged distance of 2927.10 cM (Kosambi) and having 2.6 cM resolution was constructed by genotyping 10 parents and 150 offspring from the National Center for Cool and Cold Water Aquaculture (NCCCWA) reference family mapping panel. Microsatellite markers, representing pairs of loci resulting from an evolutionarily recent whole genome duplication event, identified 180 duplicated regions within the rainbow trout genome. Microsatellites associated with genes through expressed sequence tags or bacterial artificial chromosomes produced comparative assignments with tetraodon, zebrafish, fugu, and medaka resulting in assignments of homology for 199 loci.

**Conclusion:**

The second generation NCCCWA genetic map provides an increased microsatellite marker density and quantifies differences in recombination rate between the sexes in outbred populations. It has the potential to integrate with cytogenetic and other physical maps, identifying paralogous regions of the rainbow trout genome arising from the evolutionarily recent genome duplication event, and anchoring a comparative map with the zebrafish, medaka, tetraodon, and fugu genomes. This resource will facilitate the identification of genes affecting traits of interest through fine mapping and positional cloning of candidate genes.

## Background

Genetic maps characterizing the inheritance patterns of traits and markers have been developed and utilized for a wide range of species and applications associated with studies addressing biomedical, agricultural, ecological and evolutionary questions. These applications often target the discovery of allelic variation affecting traits and have the eventual goal of identifying the exact DNA sequences underlying phenotypes [1]. Other studies highlight differences in recombination rates between the sexes as observed in the earliest linkage maps [2-4], or suggest mechanisms of chromosomal evolution through the identification of regions of conserved synteny across species. For instance, comparisons of conserved synteny and conserved gene orders have been conducted extensively within the vertebrates [5-8]. Whole genome sequences have enabled comparative genomic studies which employ computational approaches [9], including the identification of functional elements [10,11]. As a result, one additional goal for mapping in species not having access to a whole genome reference sequence is to develop high-density comparative maps with whole genome sequences of related species. This may be accomplished through genetic, cytogenetic, radiation hybrid, bacterial artificial chromosome (BAC), and integrated mapping approaches [12-16]. These maps not only enable the use of information across species, but may be used within a species to aid whole genome sequence assembly [17].

The construction of genetic maps for many species begins by genotyping reference families with markers such as microsatellites [18] which were initially developed for population genetic analyses. Microsatellites are often the marker of choice as they exhibit co-dominant inheritance, have high degrees of heterozygosity, are widely distributed throughout the genome, and may provide comparative information between closely related species[19]. When associated with a gene, these markers can provide comparative information across a great diversity of taxa. The limiting factors of microsatellites for map construction are the time and resources required for marker development and genotyping. Alternatively, amplified fragment length polymorphisms (AFLPs) [20] and random amplified polymorphic DNAs (RAPDs) [21] markers are inexpensive to develop and are conducive to high throughput genotyping protocols. Although large numbers of loci can be mapped rather inexpensively in a short amount of time, these markers are not associated with unique sequences and are specific to each mapping population. These efforts result in first generation linkage maps containing hundreds of markers which represent much of the genome with a low resolution of microsatellites [22-28]. As additional markers become available, including those associated with candidate genes, second generation maps containing several hundred to over one thousand markers spanning the entire genome at higher resolutions are constructed [29-38]. The ultimate genetic maps have sub-centiMorgan (cM) resolution and include anywhere from thousands to millions of markers [39-41]. Currently, high-density mapping efforts for human, model organism, and agriculturally important species with whole genome sequences are using single nucleotide polymorphism (SNP) [42] markers. Similar to microsatellites, SNPs are abundant, widely distributed throughout the genome, and are associated with a unique sequence. Although SNPs are amenable to genotyping with high-throughput protocols, they are less polymorphic and will require large numbers of crosses for mapping.

The status of rainbow trout genetic maps has progressed significantly over the last decade due to interest in their economic impacts as an aquaculture species and on sport fisheries, and as a model research organism for studies related to carcinogenesis, toxicology, comparative immunology, disease ecology, physiology and nutrition [43]. Rainbow trout have a genome size estimated to be 2.4 × 10^9 ^bp [44]. While the karyotype of this species varies from 2 N = 58–64, the number of chromosome arms is conserved at 104. An evolutionarily recent whole genome duplication event is estimated to have occurred 25–100 mya [45], and the genome is estimated to be 1/3 of the way along the process of re-diploidization, [46]. Several laboratories have constructed genetic maps including AFLPs, microsatellites, and SNPs to identify quantitative trait loci (QTL) affecting time to hatch, development rate, growth, thermal tolerance, natural killer cell-like activity, albinism and disease resistance [47-56]. The first genetic map based on molecular markers was constructed by Young *et al*. [57] who observed the inheritance of 476 loci (332 AFLPs) on 76 doubled haploid fish. The resulting map contained 31 large linkage groups and spanned a total of 2627.5 cM. In 2000, Sakamoto *et al*. [19] mapped 208 loci (191 microsatellites) to 29 linkage groups by genotyping 186 fish from 3 backcross families. This effort revealed large recombination rate differences between the sexes (3.25:1 female to male) and a female map length over 1000 cM Morgans which is an underestimation as acknowledged by the authors. In 2003, Nichols *et al*. [58] added to the map of Young *et al*[57], ordering 1359 loci consisting primarily of 973 AFLPs and 226 microsatellites, and forming 30 large linkage groups with a map length of 4590 cM. Most recently, Guyomard *et al. *[59] used 120 offspring from two doubled haploid mitogynogenetic families to map 903 microsatellite loci with a map length of 2750 cM. Concurrently, Phillips *et al*. [60] integrated the cytogenetic and genetic maps by assigning linkage groups from Nichols *et al*[61] to specific chromosomes of the OSU doubled haploid line. Using microsatellites as comparative loci between salmonids, Danzmann *et al. *[62] added genetic markers to the map of Sakamoto *et al*. [19], reporting homeologous chromosome arm assignments within species resulting from the genome duplication event and pairwise homologous assignments between rainbow trout, artic char and Atlantic salmon.

In an effort to support the selective breeding of rainbow trout for aquaculture production efficiency, we constructed a genetic map to identify QTL affecting important traits and facilitate positional candidate cloning [63-66]. Most of the markers mapped were anonymous microsatellites from random enriched libraries, but our focus was to add markers with comparative information between the trout genome and other salmonids, and with the genome sequences of model fish species. By genotyping 30 offspring from each of 5 outbred families related to our broodstock germplasm, 1124 loci were ordered into 29 linkage groups representing each chromosome. This allowed for the observation of differences in recombination rate between the sexes and the creation of comparative maps. Anchoring of EST sequence on the genome sequences of other fishes has enabled the construction of comparative maps to facilitate genome research in regions of interest. The development and mapping of a large number of microsatellite loci will facilitate genome mapping efforts in rainbow trout and other salmonids.

## Results

### Genotyping

A total of 1435 microsatellite markers were developed or obtained from the literature including anonymous markers [19,59,61,67-80], markers developed in other salmonids [62,81-83], markers identified from BACs either containing genes or cytogenetic assignments [60,84], or markers representing expressed sequence tags (ESTs) and serving as comparative loci with sequenced genomes of model fish species [80,85] (see Additional File 1, Worksheet 1). In all, 930 new microsatellite markers were developed and mapped for this project. These markers were genotyped on the 10 parents and 150 offspring of the NCCCWA reference family panel. One hundred twenty three markers were scored as duplicates containing two sets of segregating alleles resulting in evaluation of 1558 loci. Of the 1435 markers attempted, 268 amplified poorly and were discarded. Another 87 were not informative in our reference families. A total of 1181 loci were informative for either the male or female map, with 1100 loci informative in the female and 1068 loci informative for the male; 991 loci were informative for both sexes (See Additional File 1, Worksheet 6). A total of 18 loci were observed to demonstrate pseudolinkage in the males [86], therefore only female data for those markers were included for linkage analysis.

### Linkage Analysis

**Figure 1:**
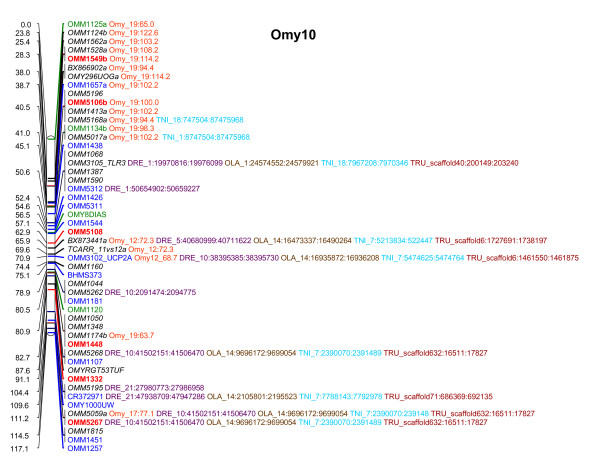
**Genetic map of rainbow trout chromosome 10 including comparative assignments**. Recombination distances are presented as total Kosambi cM for each map on the left, marker names and comparative assignments are on the right. Loci names in **red bold font **are ordered at LOD 4.0, loci in blue font at LOD 3.0, loci in green font at LOD 2.0, and loci in *black italic *at LOD 0.0. Although Omy10 did not have any loci in black font mapping at LOD 1.0, this convention is used for the whole map provided in Additional File 2. Paralogous assignments within the rainbow trout genome are presented in orange in the format Omy_1:10.0 where Omy is the three letter species designation for rainbow trout (Oncorhynchus mykiss), 1 is the chromosome number of the paralogous locus and 10.0 is its map position. Comparative assignments are similarly presented next to loci names in the format species abbreviation_chromosome number:homolog start nucleotide:homolog end nucleotide. Comparative assignments for zebrafish (DRE) are presented in purple, for medaka (OLA) in brown, for tetraodon (TNI) in light blue, and fugu (TRU) in maroon. All chromosomes are presented in this format in Additional File 2.

**Figure 2:**
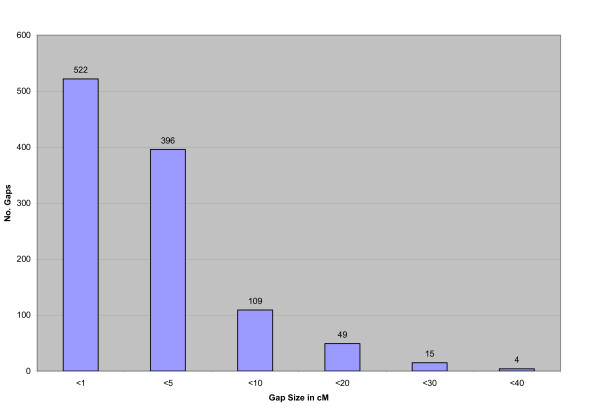
**Gaps in the rainbow trout genetic map**. The NCCCWA Genetic map has an average resolution of one marker per 2.6 cM and contains 1095 gaps, ~84% of which are less than 5 cM and ~94% are less than 10 cM. Figure 2 shows the number of gaps observed in the NCCCWA genetic map and their sizes in terms of cM.

**Figure 3:**
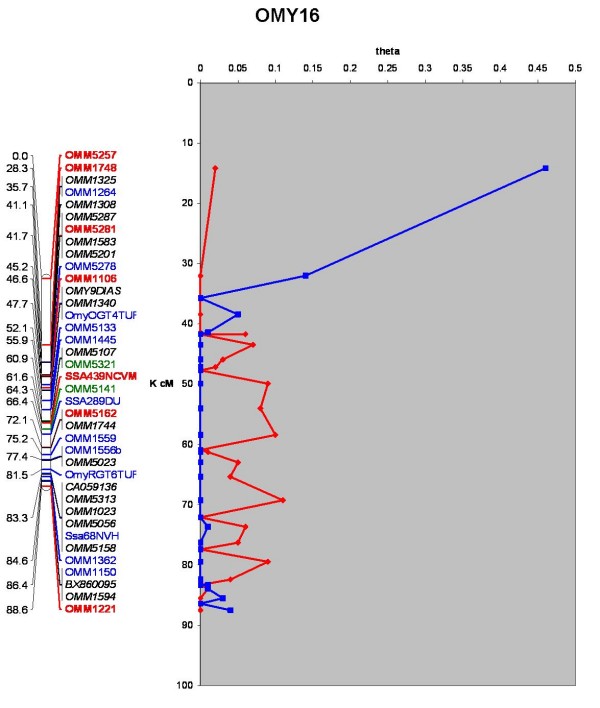
**Chromosome specific variation in recombination rate between sexes**. The genetic map of OMY16 is presented on the left, on the right a chart of male (blue line) and female (red line) recombination fractions between each pair of loci. All chromosomes are presented in this format in Additional File 4. The map length is presented in terms of Kosambi cM, the pairwise recombination fractions (theta) between each marker pair along the length of the chromosome map is presented.

Two-point linkage analysis placed 1124 loci into 29 major linkage groups having 16 or more loci at LOD ≥ 10, an additional 56 were informative but were not localized to single linkage groups with significant LOD scores (See Additional File 1, Worksheet 10). The total combined sex averaged map distance was 2927.10 cM (Kosambi), a map representing OMY10 is presented in Figure 1, and maps representing all chromosomes are presented in Additional Files 2 (comparative map) and 3 (genetic map only). The numbers of markers included in a framework map created at LOD ≥ 4 was 137 and ranged from 0–9 markers per chromosome. Chromosomes OMY1, 5, 21, or 23 did not contain any framework markers. Additional loci were added at LOD ≥ 3 (343), ≥ 2 (67) ≥ 1 (30), and ≥ 0 (547). Additional File 1, Worksheet 10 contains this information and can be used to recreate maps using MapChart software [87]. The average resolution of the genome map was 2.6 cM with individual chromosome maps ranging from 1.64 to 4.09 cM. The map contains 1095 gaps, ~84% of which are less than 5 cM and ~94% are less than 10 cM (See Figure 2). The markers used to assign linkage groups to the cytogenetic map of Phillips *et al*. [60] are presented in Additional File 1, Worksheet 7 in addition to markers comparing linkage groups across genetic maps as modified from Guyomard *et al*. and Nichols *et al*. reported in Worksheet 8 [59,61]. Overall statistics for the map and each chromosome are given in Additional File 1, Worksheet 9 including chromosome, number of markers, number of markers from ESTs, number of markers from BACs, number of markers with homology assignments to genome sequences of 4 model fish species, number of markers ordered at each LOD threshold, total map length, female map length, male map length, female:male recombination ratios, recombination rate correlations and map resolution. The female:male recombination ratio was 1.68:1, with the female having a map length of 4,317.60 cM and the male map 2,564.10 cM. This ratio varied by chromosome, ranging from 0.73:1 to 12.22:1 (Kosambi) (See Table 1). Figure 3 shows a map representing the differences in recombination rate between the sexes along the chromosome length for OMY16. Maps for the other chromosomes are presented in Additional File 4.

**Table 1:** Chromosome specific differences in recombination rates between sexes

**Chr**	**Sex-Averaged cM**	**Female cM**	**Male cM**	**Female:Male**
1	96.8	218.8	17.9	12.22
2	93	128.7	62.4	2.06
3	142.2	187	103.9	1.8
4	120.5	182.8	108.9	1.68
5	109.8	118.1	161.9	0.73
6	82.2	146.9	54.2	2.71
7	128.3	164.6	86.9	1.89
8	115.5	122.3	118.3	1.03
9	106.4	113.7	92.9	1.22
10	117.1	214.6	79	2.72
11	125.9	189.5	99.7	1.9
12	139.2	215.2	105.6	2.04
13	94.7	95.4	70.2	1.36
14	130	146	167.5	0.87
15	101.8	237.3	51.8	4.58
16	88.6	94.7	110.3	0.86
17	136.5	218.8	91.9	2.38
18	110.2	225.3	81.2	2.77
19	155.6	242.6	236.5	1.03
20	105.7	115.6	98.8	1.17
21	83.1	117.9	57.3	2.06
22	61.2	68.4	83.5	0.82
23	57.2	153.8	19.3	7.97
24	37.6	45.8	29.8	1.54
25	146.9	178.2	142.2	1.25
26	58	162	26.4	6.14
27	74.3	86.2	74.4	1.16
28	50.3	64.1	63.6	1.01
Sex	58.5	63.3	67.8	0.93

**total**	**2,927.10**	**4,317.60**	**2,564.10**	**1.68**

The overall female:male recombination ratio was 1.68:1, with the female having a map length of 4,317.60 cM and the male map 2,564.10 cM. However, individual chromosome and regions of chromosomes varied, with a range of .73:1 to 12.22:1. This table shows the sum of sex averaged (r total), female (r F) and male (r M) recombination fractions between adjacent pairs of loci and the ratio of female to male (F:M). In some cases the sex-averaged recombination rate is lower than that for either of the sexes, this is because different sets of loci are informative for each analysis.

### Homeologous Assignments

**Figure 4:**
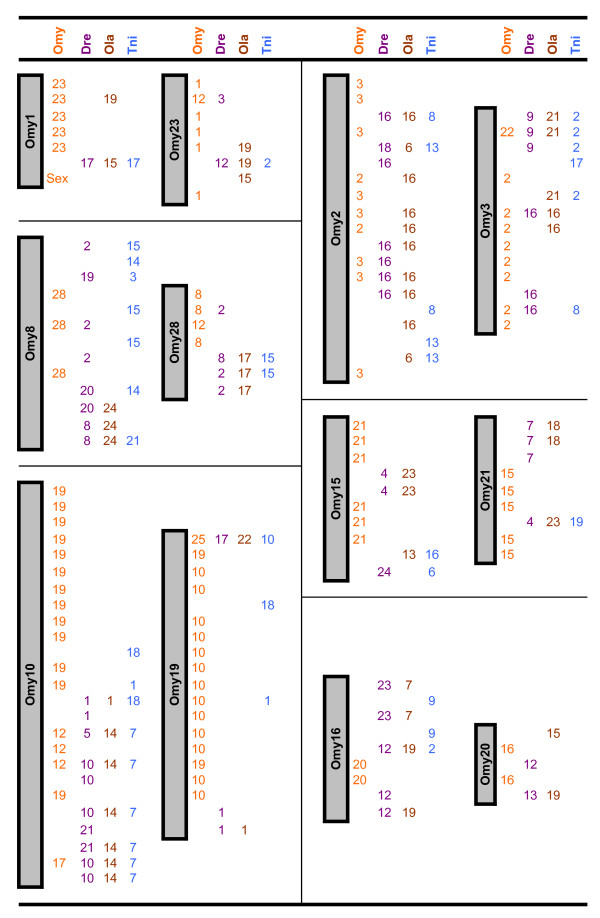
**Comparative maps**. The rainbow trout genetic map serves as a comparative map within its own genome and with the genome sequences of zebrafish (DRE), medaka (OLA), and tetraodon (TNI). Assignments for fugu scaffolds are reported in Additional File 1 Worksheets 2 (ESTs – Comparative Annotation) and 5 (Comparative Info – BACs). Figure 4 shows rainbow trout chromosomes 1, 2, 3, 8, 10, 15, 16, 19, 20, 21, 23 and 28 represented on the left, with regions of homeology identified through duplicated markers from the genetic map reported under the OMY column on the right. Figure 5 similarly shows the comparative map for chromosomes 6, 7, 11, 12, 13, 14, 17, 18, and 26, Figure 6 contains the comparative information for chromosomes 4, 5, 9, 22, 24, 25, 27 and Sex. Chromosomes showing large regions of homeology are grouped together. Further to the right chromosome homology assignments identified through comparative annotation are reported for each species (DRE, OLA, TNI). Many assignments have been made through comparative mapping of single loci, however wherever two or more loci define a region of conserved synteny we have not tried to estimate the size of the conserved fragment.

**Figure 5:**
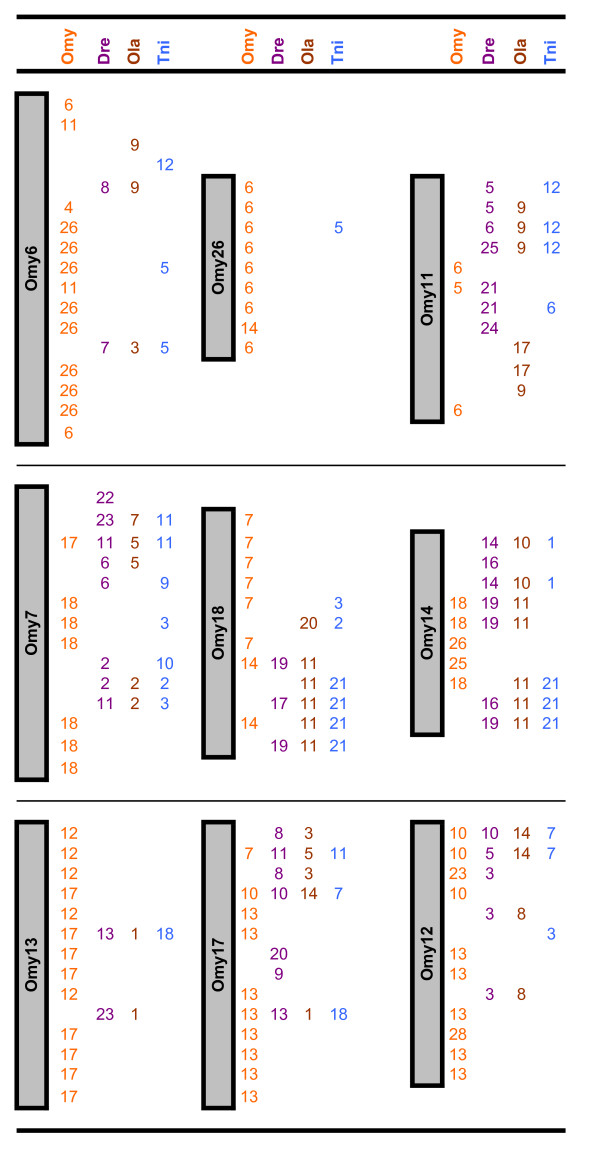
**Comparative maps**. The rainbow trout genetic map serves as a comparative map within its own genome and with the genome sequences of zebrafish (DRE), medaka (OLA), and tetraodon (TNI). Assignments for fugu scaffolds are reported in Additional File 1 Worksheets 2 (ESTs – Comparative Annotation) and 5 (Comparative Info – BACs). Figure 4 shows rainbow trout chromosomes 1, 2, 3, 8, 10, 15, 16, 19, 20, 21, 23 and 28 represented on the left, with regions of homeology identified through duplicated markers from the genetic map reported under the OMY column on the right. Figure 5 similarly shows the comparative map for chromosomes 6, 7, 11, 12, 13, 14, 17, 18, and 26, Figure 6 contains the comparative information for chromosomes 4, 5, 9, 22, 24, 25, 27 and Sex. Chromosomes showing large regions of homeology are grouped together. Further to the right chromosome homology assignments identified through comparative annotation are reported for each species (DRE, OLA, TNI). Many assignments have been made through comparative mapping of single loci, however wherever two or more loci define a region of conserved synteny we have not tried to estimate the size of the conserved fragment.

**Figure 6:**
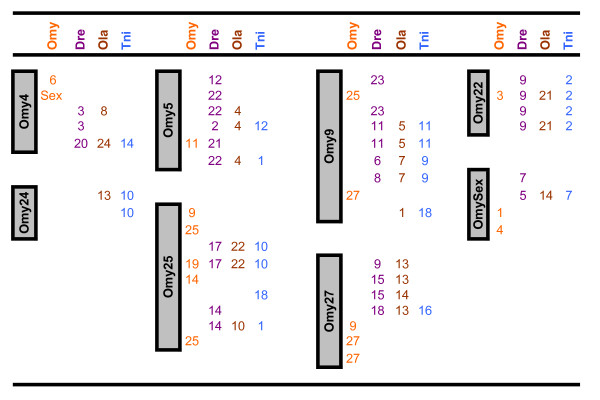
**Comparative maps**. The rainbow trout genetic map serves as a comparative map within its own genome and with the genome sequences of zebrafish (DRE), medaka (OLA), and tetraodon (TNI). Assignments for fugu scaffolds are reported in Additional File 1 Worksheets 2 (ESTs – Comparative Annotation) and 5 (Comparative Info – BACs). Figure 4 shows rainbow trout chromosomes 1, 2, 3, 8, 10, 15, 16, 19, 20, 21, 23 and 28 represented on the left, with regions of homeology identified through duplicated markers from the genetic map reported under the OMY column on the right. Figure 5 similarly shows the comparative map for chromosomes 6, 7, 11, 12, 13, 14, 17, 18, and 26, Figure 6 contains the comparative information for chromosomes 4, 5, 9, 22, 24, 25, 27 and Sex. Chromosomes showing large regions of homeology are grouped together. Further to the right chromosome homology assignments identified through comparative annotation are reported for each species (DRE, OLA, TNI). Many assignments have been made through comparative mapping of single loci, however wherever two or more loci define a region of conserved synteny we have not tried to estimate the size of the conserved fragment.

The frequency of duplicated microsatellite loci in rainbow trout is very high as a result of the recent salmonid whole genome duplication. We were able to score two loci for 123 markers, denoting the loci names with a lower case "a" or "b." Although two loci were informative for all 123 of these markers, in many cases only one locus was successfully ordered on the map. Our map revealed 180 duplicated marker regions, including 30 pairs of homeologues from ESTs and 10 pairs identified from BACs that harbor genes of interest (Figures 4, 5, 6). In addition, 149 loci are potential duplicates with monomorphic amplicons at a putative second locus.

### Comparative Assignments

The ability to use microsatellites developed from one salmonid in other salmonid species provides comparative mapping information. To this end, our map includes 33 markers from Atlantic salmon, 8 from sockeye salmon, 3 from pink salmon, and 1 from Chinook salmon. In addition to comparative maps with the salmonids, our map includes markers representing 325 ESTs and 57 loci from BACs that harbor genes of interest. These have the potential to serve in developing comparative maps with the genome sequences of model fish species. Additional File 1, Worksheet 2 contains comparative assignments of homology for markers developed from ESTs, which have markers names in the OMM5000 series or GenBank accession numbers. Two strategies were used to assign functional annotation to these markers. First, Worksheet 3 contains functional annotation for those markers derived directly from blastx hits. Secondly, Worksheet 4 identifies the corresponding Unigene [88] or Rainbow Trout Gene Index [89] record, including the EST from which each marker was designed. Functional annotation through BLAST and GO assignments are available through these resources. Worksheet 5 contains assignments of comparative homology for markers derived from BACs that contain genes of interest. These marker names are in the OMM3000 series. Homologs for 199 loci were identified in zebrafish (146), medaka (123), tetraodon (164), or fugu (131) (Figures 4, 5, 6).

## Discussion

### Map Overview

The NCCCWA genetic map of rainbow trout was constructed by observing the inheritance of 1124 microsatellite markers in 5 families containing 30 offspring each. Although all linkage groups were identified with a high level of confidence, many markers were ordered at low LOD scores, primarily the result of a low number of informative meiosis. This was especially true for most duplicated markers where only one family with a maximum of 30 offspring could be scored. The map contains many markers which provide comparative information by identifying regions of homeology within the trout genome or regions of conserved synteny with genome sequences of model fish species. We observed whole genome map lengths of 4317.6 cM and 2564.1 cM for females and males, respectively, which is similar to the distances reported by Young *et al*. (2627.5 cM)[90], Nichols *et al*. (4590 cM) [61], and Guyomard *et al*. (2750 cM) [59]. However, our female map length differs significantly from the 10 Morgans reported by Sakamoto *et al*. [19] who reported the differences in sex recombination ratio to be 3.25:1. We observed an average sex recombination ratio of 1.68:1, but it varied greatly by chromosome and sub-chromosomal region. One explanation is that the microsatellites used in our map and the AFLPs used in the previous map differ with respect to their co-location with recombination hot spots. Another explanation is likely due to the marker densities on specific linkage groups which show higher ratios than the rest of the map. The chromosome specific ratios of 12.22:1, 4.58:1, 7.97:1, and 6.14:1, observed for chromosomes OMY 1, 15, 23, and 26, respectively, are well outside of the range for the rest of the chromosomes (.73:1 – 2.77:1). Having less drastic difference in this ratio than observed by Sakamoto *et al*. [19] facilitated the construction of a sex-averaged map in which we could include loci informative in any one of the 10 parents in the NCCCWA mapping reference families. However, the differences in recombination ratios are significant and sex should be accounted for when designing QTL experiments.

### Genome Duplication

Due to the evolutionarily recent whole genome duplication event, many microsatellite markers in salmonids exist as two copies in the genome, frequently resulting in two loci which can be genotyped per primer set [19,43,45,46,78,80,91]. In some instances the two loci can be distinguished due to drastic differences in allele sizes, but more often the loci have overlapping and identical allele sizes and include null alleles. In the latter case, the loci often can be scored in only one family, reducing the observed number of informative meioses supporting map construction. The benefit of these markers is that they identify chromosome fragments that probably share a common ancestor, and are likely to have similar complements of genes in various states of re-diploidization. As presented in Figures 4, 5, 6, we identified 180 assignments of homeology in the rainbow trout genome. As observed previously [19,59,61,62], several chromosomes showed homeology primarily with one other chromosome, including the pairs OMY1/OMY23, OMY8/OMY28, OMY10/OMY19, OMY2/OMY3, OMY15/OMY21, OMY16/OMY20, OMY6/OMY26, OMY7/OMY18, and OMY13/OMY17. Chromosomes OMY2, OMY6, OMY19, OMY25, and OMY27 showed regions of homeology within the chromosome. The mapping of duplicated microsatellites from BACs and ESTs suggests that the gene complements of these regions may be similar and is useful for comparative mapping these regions with other salmonids and with the genomes of model fish species.

### Comparative Assignments

Through the development of microsatellite markers from 325 ESTs and 57 BACs, we identified homologs for 199 loci in zebrafish, medaka, tetraodon, and/or fugu for the construction of a comparative map. Assignments include 146 for zebrafish, 123 for medaka, 164 for tetraodon, and 131 for fugu. As the fugu genome is not fully assembled, we report comparative assignments only for zebrafish, medaka, and tetraodon in Figures 4, 5, 6. There were 34, 30, and 22 comparative assignments for zebrafish, medaka, and tetraodon, respectively, where more than 2 markers from the same chromosome were assigned to the same rainbow trout chromosome. There were 29, 26, and 17 blocks of conserved synteny as defined by two or more consecutive assignments from the same chromosome for zebrafish, medaka, and tetraodon, respectively. These assignments of homology will facilitate candidate gene discovery, potentially providing comparative genome sequence information to marker intervals of interest (e.g. from QTL detection experiments).

## Conclusion

This second generation NCCCWA rainbow trout genetic map provides an increased microsatellite marker density, estimates of sex specific recombination rates across the genome of outbred populations and a framework for producing an integrated genetic and physical map. The map identifies paralogous regions of the rainbow trout genome arising from the evolutionarily recent salmonid genome duplication, and serves as a starting point for comparative maps with the zebrafish, medaka, tetraodon, and fugu genomes. This resource will facilitate the identification of genes affecting traits of interest through fine mapping and positional candidate cloning.

## Methods

### Reference Family Panel

Reference families for mapping studies were selected from the National Center for Cool and Cold Water Aquaculture's 2002 brood year including 10 parental fish originating from the following strains: Clear Spring (CS), Troutlodge (TL), and Donaldson from the University of Washington (UW) [92]. The majority of karyotypes for fish related to the parents were determined to have 2 N = 58 chromosomes, with low frequencies of variation of up to 2 N = 64. Parental fin clips and 30 offspring from each mating, including one intra-strain cross (CS × CS) and 4 inter-strain crosses (2 TL × UW, 2 UW × TL), were sampled for DNA extractions using the phenol-chloroform method described in Sambrook and Russell [93]. DNA samples were quantified by spectrophotometer (Beckman DU 640, Beckman Instruments, St. Louis, MO, USA) and diluted to a concentration of 12.5 ng/ul for PCR.

### Microsatellite Genotyping

A total of 1435 microsatellite markers were developed or obtained from the literature including anonymous markers [19,59,61,67-80], markers developed in other salmonids [62,81-83], markers identified from BACs either containing genes or cytogenetic assignments [60,84,94-96], or markers representing expressed sequence tags (ESTs) and serving as comparative loci with sequenced genomes of model fish species [80,85]. Marker information including locus names, optimum annealing temperatures and magnesium concentrations, GenBank accession numbers, and primer sequences are reported in Additional File 1, Worksheet 1. Markers were either genotyped using the tailed protocol of Boutin-Ganache *et al*. [97] or by direct fluorescent labelling (with FAM, HEX, or NED) of the forward primer according to manufacturer protocols (ABI, Foster City, CA, USA). Primer pairs were obtained from commercial sources (forward primers labelled with FAM or HEX from Alpha DNA, Montreal, Quebec, Canada, or NED from ABI, Foster City, CA, USA). PCR reactions consisted of 12 μl reaction volumes containing 12.5 ng DNA, 1.5–2.5 mM MgCl_2_, 1.0 μM of each primer, 200 μM of dNTPs, 1× manufacturer's reaction buffer and 0.5 units Taq DNA polymerase. Thermal cycling consisted of an initial denaturation at 95°C for 15 min followed by 30 cycles of 95°C for 1 min, annealing temperature for 45 s, 72°C extension for 45 s, then a final extension at 72°C for 10 min. PCR products were visualized on agarose gels after staining with ethidium bromide. Markers were grouped in combinations of two or three markers based on differences in fluorescent dye color and amplicon size. Three μl of each PCR product was added to 20 μl of water, 1 μl of the diluted sample was added to 12.5 μl of loading mixture made up with 12 μl of HiDi formamide and 0.5 of Genscan 400 ROX internal size standard. Samples were denatured at 95°C for 5 min and kept on ice until loading on an automated DNA sequencer ABI 3730 DNA Analyzer (ABI, Foster City, CA, USA). Output files were analyzed using GeneMapper version 3.7 (ABI, Foster City, CA, USA), formatted using Microsoft Excel and stored in Microsoft Access. As a result of the evolutionarily recent genome duplication, microsatellite markers in salmonids are often present in two copies in the genome, each copy potentially having overlapping allele size ranges and possibly including alleles having identical sizes. Markers which were duplicated were scored as independent loci, adding an "a" and "b" to differentiate their locus names. Duplicated loci with overlapping and/or identical allele sizes were scored only in the family containing the most informative meiosis.

### Linkage Analyses

Genotype data combined for both sexes were formatted using MAKEPED of the LINKAGE [98] program and checked for inconsistencies with Mendelian inheritance using PEDCHECK [99]. RECODE [100] and LNKTOCRI [101] were used to assemble the data into CRIMAP [102] format. MULTIMAP [103] was used to conduct two-point and multi-point linkage analyses. Two-point linkage analysis included parameters of LOD ≥ 10 and recombination fraction r ≤ 0.5. Multipoint linkage analysis was conducted on individual linkage groups, including loci unlinked at LOD ≥ 10 but linked to loci in that linkage group at LOD ≥ 4. Framework maps were constructed using default parameters, markers were added to comprehensive maps by lowering the LOD threshold one integer at a time and starting with the previous order. Resulting maps are consensus maps, accounting for co-informative meiosis across the five families.

### Linkage Group Nomenclature

Linkage groups were assigned chromosome names using the integrated cytogenetic/linkage map of Phillips *et al*. [60]. Specific markers used to identify cytogenetic chromosome names are listed in Additional File 1 Worksheet 7 (Markers for Map Integration). In an effort to identify common linkage groups between published maps, Additional File 1 Worksheet 8 (Linkage Group Translation) has been adapted from Guyomard *et al*. [59].

### Estimating Differences in Recombination Rates between the Sexes

Multimap reports sex averaged, female and male recombination rates for any given map order. Whole-genome map lengths were obtained by adding the total cM for each chromosome for the sex-averaged, female, and male maps. To estimate the genome wide female:male recombination ratio, the entire map length for the female was divided by that of the male. To evaluate chromosome specific rates, pairwise distances in cM between adjacent map intervals were calculated and presented in Figure 3 and Additional File 4, chromosome specific ratios are reported in Additional File 1 Worksheet 9 (Chromosome Information).

### Comparative Assignments

Expressed Sequences associated with microsatellites (OMM5000 and GenBank accession no. designations) for markers were BLASTed [104] using blastn against the transcripts of each genome obtained from . Only matches having a minimum alignment length over 50 bp and percent identity over 78% were treated as potential matches. Data were hand checked and assignments which were questionable were removed. Microsatellites identified from bacterial artificial chromosomes were annotated with genes known to be contained within those clones by sequence analysis.

## Authors' contributions

CER designed the study, collected genotypes and conducted the linkage analysis. RV developed the linkage analysis pipeline and participated in linkage analysis, YP participated in marker development and genotyping, SAG participated in marker development. Dr. Jeffrey Silverstein participated in cross design and execution.

## Supplementary Material

Additional file 1 **Marker and Mapping Information**. Additional Files are included as numbered spreadsheets in a MS Excel file:
1 Marker PCR Information (PCR primers, conditions, etc...)
2 ESTs – Comparative Assignments (identification of homologs of OMM5000 markers, developed from ESTs, in the zebrafish, medaka, tetraodon, and fugu genomes)
3 ESTs – BLASTx Annotation (Functional annotation for EST markers assigned through blastx of the GenBank nr database)
4 ESTs – RTGI and Unigene (Identification of Rainbow Trout Gene Index and Unigene Assemblies for which EST markers belong)
5 Comparative Info – BACs (Comparative information for OMM3000 markers derived from BACs containing genes of interest)
6 Inf Mei and Allele#s (informative meiosis and number of alleles for markers screened on the NCCCWA reference families)
7 Markers for Map Integration (Markers used to integrate NCCCWA linkage groups and the rainbow trout cytogenetic map)
8 Linkage Group Translation (identifying names of common linkage groups between published rainbow trout genetic maps)
9 Chromosome Info (Linkage analysis information broken down by chromosome)
10 2pt Info for loci informative but not mapped (Reporting loci which were informative in the NCCCWA reference families but not able to be placed on the map)
11 Maps (Genetic map data which can be used to recreate map figues in MapChart) Click here for file

Additional file 2 **Genetic Map**. This Adobe PDF file includes figures representing the 29 linkage groups/chromosomes of the NCCCWA rainbow trout genetic map. Recombination distances are presented as total Kosambi cM for each map on the left, marker names and comparative assignments are on the right. Loci names in red bold font are ordered at LOD 4.0, loci in blue font at LOD 3.0, loci in green font at LOD 2.0, loci in black font at LOD 1.0 and loci in *black italic *at LOD 0.0. Paralogous assignments within the rainbow trout genome are presented in orange in the format Omy_1:10.0 where Omy is the three letter species designation for rainbow trout (*Oncorhynchus mykiss*), 1 is the chromosome number of the paralogous locus and 10.0 is its map position. Comparative assignments are similarly presented next to loci names in the format species abbreviation_chromosome number:homolog start nucleotide:homolog end nucleotide. Comparative assignments for zebrafish are presented in purple, for medaka in brown, for tetraodon in light blue, and fugu in maroon. Click here for file

Additional file 3 **Genetic Map**. This Adobe PDF file includes figures representing the 29 linkage groups/chromosomes of the NCCCWA rainbow trout genetic map. Recombination distances are presented as total Kosambi cM for each map on the left, marker names are on the right. Loci names in red bold font are ordered at LOD 4.0, loci in blue font at LOD 3.0, loci in green font at LOD 2.0, loci in black font at LOD 1.0 and loci in *black italic *at LOD 0.0. Click here for file

Additional file 4 **Sex Recombination Ratios**. This Adobe PDF file includes figures representing the 29 linkage groups/chromosomes of the NCCCWA rainbow trout genetic map and figures representing the differences in recombination rate between the sexes along each chromosome. The map length is presented in terms of Kosambi cM, the pairwise recombination fractions (theta) between each marker pair along the length of the chromosome map is presented. Click here for file
