# Supplementary material for: A second generation genetic map for rainbow trout (Oncorhynchus mykiss)
Source: BMC Genet. 2008 Nov 19;9:74. doi: 10.1186/1471-2156-9-74 (PMC2605456; doi:10.1186/1471-2156-9-74)
Supplement: Additional file 2 — Genetic Map. This Adobe PDF file includes figures representing the 29 linkage groups/chromosomes of the NCCCWA rainbow trout genetic map. Recombination distances are presented as total Kosambi cM for each map on the left, marker names and comparative assignments are on the right. Loci names in red bold font are ordered at LOD 4.0, loci in blue font at LOD 3.0, loci in green font at LOD 2.0, loci in black font at LOD 1.0 and loci in black italic at LOD 0.0. Paralogous assignments within the rainbow trout genome are presented in orange in the format Omy_1:10.0 where Omy is the three letter species designation for rainbow trout (Oncorhynchus mykiss), 1 is the chromosome number of the paralogous locus and 10.0 is its map position. Comparative assignments are similarly presented next to loci names in the format species abbreviation_chromosome number:homolog start nucleotide:homolog end nucleotide. Comparative assignments for zebrafish are presented in purple, for medaka in brown, for tetraodon in light blue, and fugu in maroon. [file 1471-2156-9-74-S2.pdf]

Omy1

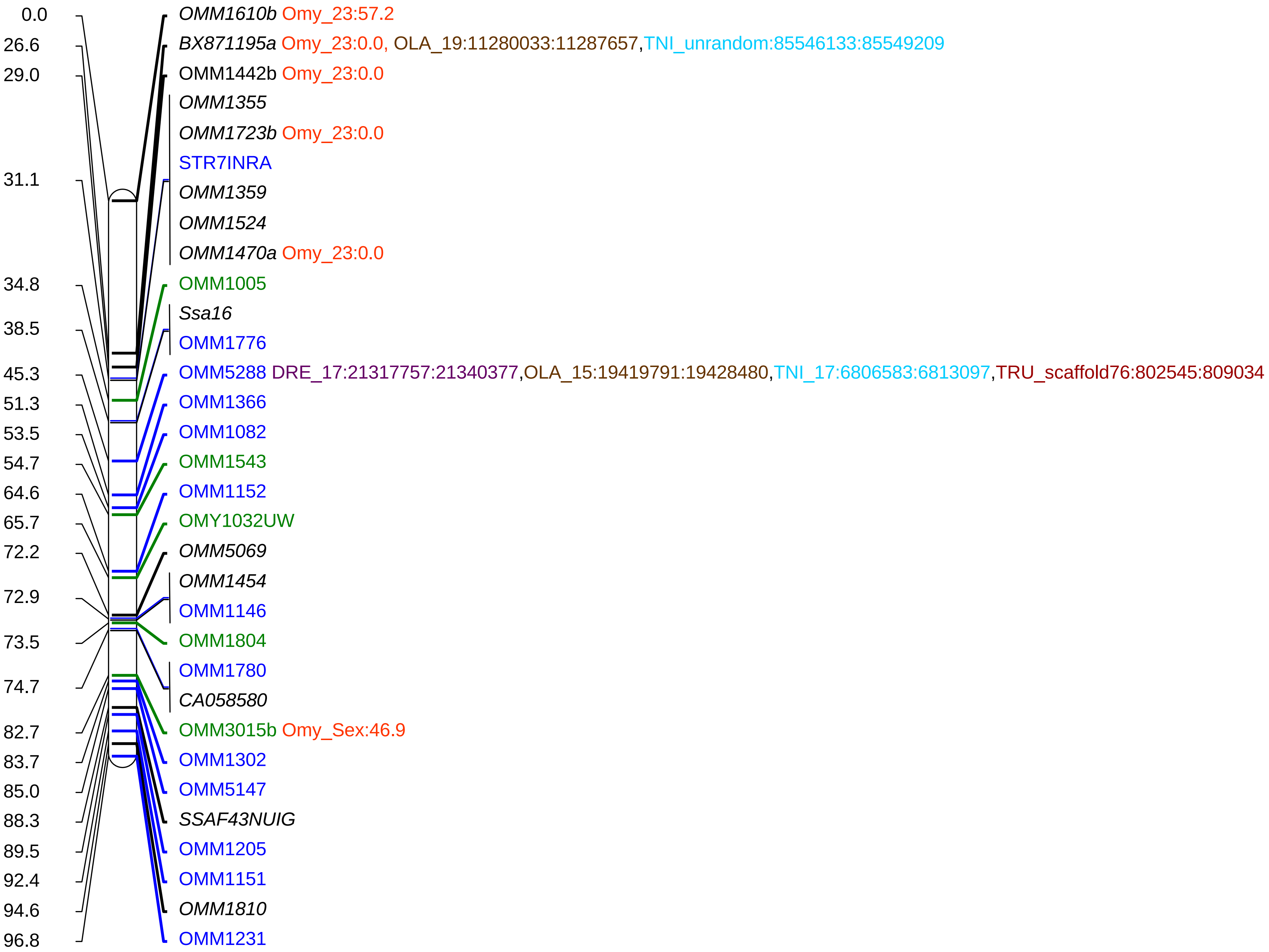

0.0
26.6
29.0
31.1
34.8
38.5
45.3
51.3
53.5
54.7
64.6
65.7
72.2
72.9
73.5
74.7
82.7
83.7
85.0
88.3
89.5
92.4
94.6
96.8
OMM1610b Omy_23:57.2
BX871195a Omy_23:0.0, OLA_19:11280033:11287657,TNI_unrandom:85546133:85549209
OMM1442b Omy_23:0.0
OMM1355
OMM1723b Omy_23:0.0
STR7INRA
OMM1359
OMM1524
OMM1470a Omy_23:0.0
OMM1005
Ssa16
OMM1776
OMM5288 DRE_17:21317757:21340377,OLA_15:19419791:19428480,TNI_17:6806583:6813097,TRU_scaffold76:802545:809034
OMM1366
OMM1082
OMM1543
OMM1152
OMY1032UW
OMM5069
OMM1454
OMM1146
OMM1804
OMM1780
CA058580
OMM3015b Omy_Sex:46.9
OMM1302
OMM5147
SSAF43NUIG
OMM1205
OMM1151
OMM1810
OMM1231

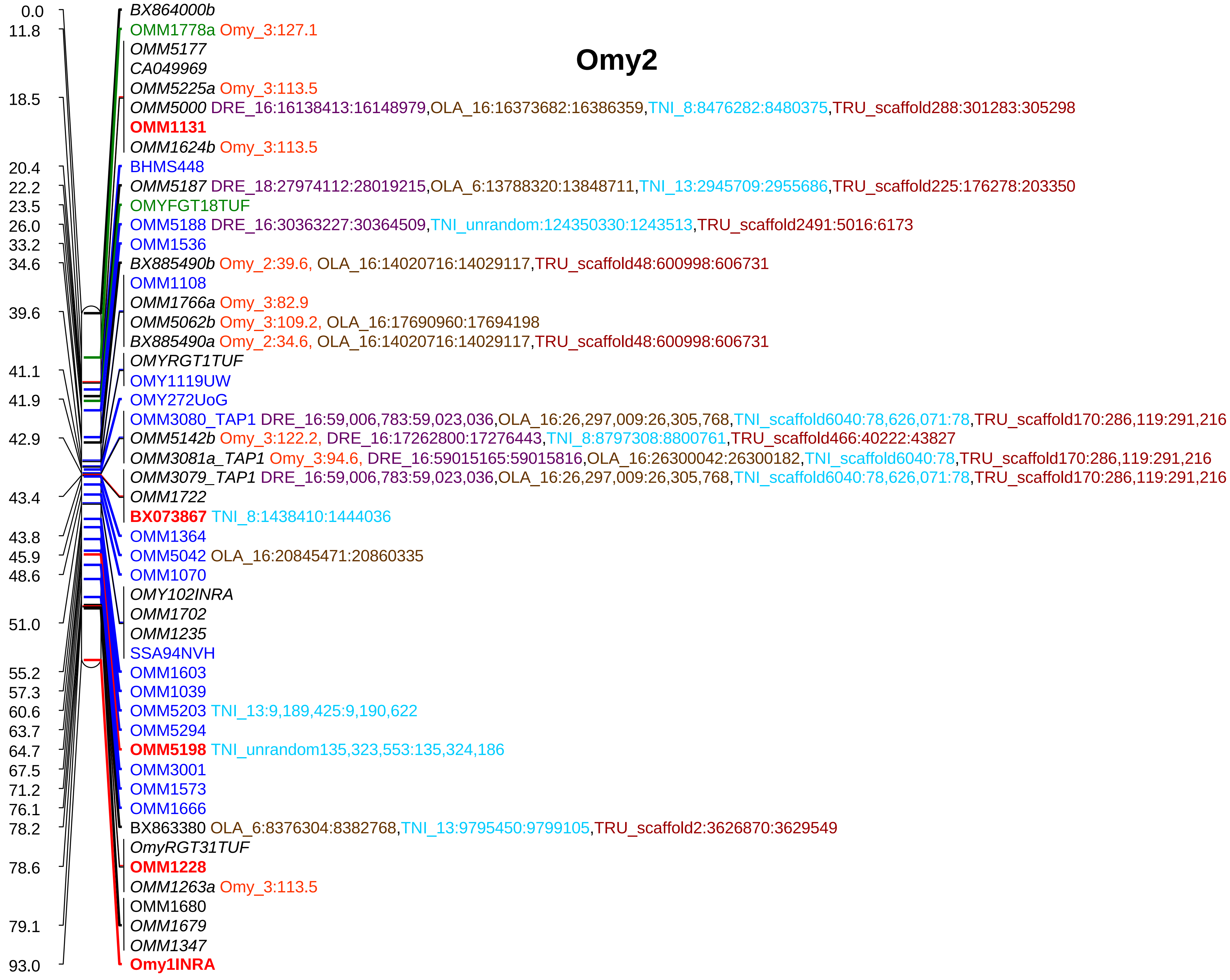
Omy2
0.0
11.8
18.5
20.4
22.2
23.5
26.0
33.2
34.6
39.6
41.1
41.9
42.9
43.4
43.8
45.9
48.6
51.0
55.2
57.3
60.6
63.7
64.7
67.5
71.2
76.1
78.2
78.6
79.1
93.0
BX864000b
OMM1778a Omy_3:127.1
OMM5177
CA049969
OMM5225a Omy_3:113.5
OMM5000 DRE_16:16138413:16148979,OLA_16:16373682:16386359,TNI_8:8476282:8480375,TRU_scaffold288:301283:305298
OMM1131
OMM1624b Omy_3:113.5
BHMS448
OMM5187 DRE_18:27974112:28019215,OLA_6:13788320:13848711,TNI_13:2945709:2955686,TRU_scaffold225:176278:203350
OMYFGT18TUF
OMM5188 DRE_16:30363227:30364509,TNI_unrandom:124350330:1243513,TRU_scaffold2491:5016:6173
OMM1536
BX885490b Omy_2:39.6, OLA_16:14020716:14029117,TRU_scaffold48:600998:606731
OMM1108
OMM1766a Omy_3:82.9
OMM5062b Omy_3:109.2, OLA_16:17690960:17694198
BX885490a Omy_2:34.6, OLA_16:14020716:14029117,TRU_scaffold48:600998:606731
OMYRGT1TUF
OMY1119UW
OMY272UoG
OMM3080_TAP1 DRE_16:59,006,783:59,023,036,OLA_16:26,297,009:26,305,768,TNI_scaffold6040:78,626,071:78,TRU_scaffold170:286,119:291,216
OMM5142b Omy_3:122.2, DRE_16:17262800:17276443,TNI_8:8797308:8800761,TRU_scaffold466:40222:43827
OMM3081a_TAP1 Omy_3:94.6, DRE_16:59015165:59015816,OLA_16:26300042:26300182,TNI_scaffold6040:78,TRU_scaffold170:286,119:291,216
OMM3079_TAP1 DRE_16:59,006,783:59,023,036,OLA_16:26,297,009:26,305,768,TNI_scaffold6040:78,626,071:78,TRU_scaffold170:286,119:291,216
OMM1722
BX073867 TNI_8:1438410:1444036
OMM1364
OMM5042 OLA_16:20845471:20860335
OMM1070
OMY102INRA
OMM1702
OMM1235
SSA94NVH
OMM1603
OMM1039
OMM5203 TNI_13:9,189,425:9,190,622
OMM5294
OMM5198 TNI_unrandom135,323,553:135,324,186
OMM3001
OMM1573
OMM1666
BX863380 OLA_6:8376304:8382768,TNI_13:9795450:9799105,TRU_scaffold2:3626870:3629549
OmyRGT31TUF
OMM1228
OMM1263a Omy_3:113.5
OMM1680
OMM1679
OMM1347
Omy1INRA

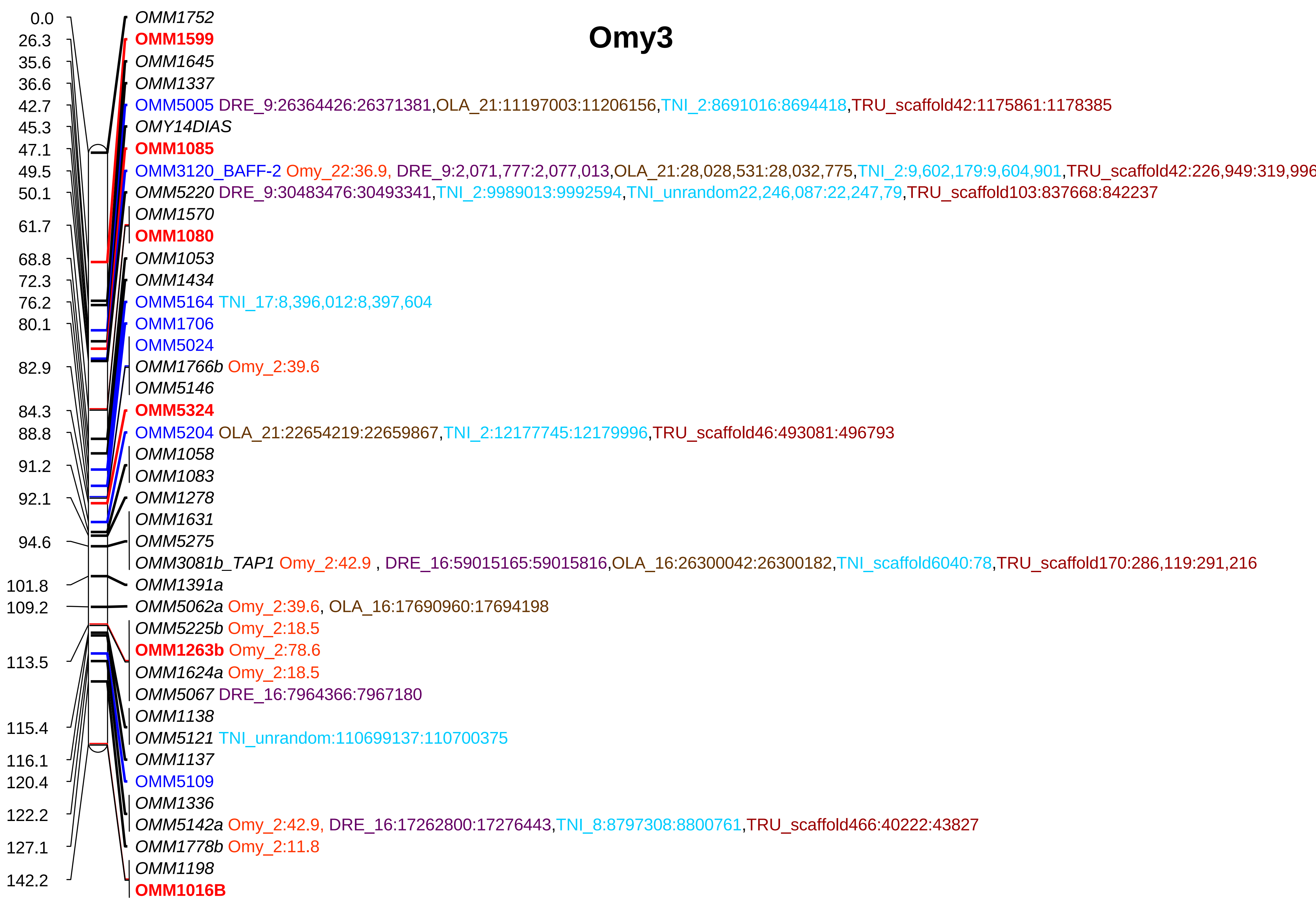
Omy3
0.0 OMM1752
26.3 OMM1599
35.6 OMM1645
36.6 OMM1337
42.7 OMM5005 DRE_9:26364426:26371381,OLA_21:11197003:11206156,TNI_2:8691016:8694418,TRU_scaffold42:1175861:1178385
45.3 OMY14DIAS
47.1 OMM1085
49.5 OMM3120_BAFF-2 Omy_22:36.9, DRE_9:2,071,777:2,077,013,OLA_21:28,028,531:28,032,775,TNI_2:9,602,179:9,604,901,TRU_scaffold42:226,949:319,996
50.1 OMM5220 DRE_9:30483476:30493341,TNI_2:9989013:9992594,TNI_unrandom22,246,087:22,247,79,TRU_scaffold103:837668:842237
61.7 OMM1570
OMM1080
68.8 OMM1053
72.3 OMM1434
76.2 OMM5164 TNI_17:8,396,012:8,397,604
80.1 OMM1706
OMM5024
82.9 OMM1766b Omy_2:39.6
OMM5146
84.3 OMM5324
88.8 OMM5204 OLA_21:22654219:22659867,TNI_2:12177745:12179996,TRU_scaffold46:493081:496793
OMM1058
91.2 OMM1083
92.1 OMM1278
OMM1631
94.6 OMM5275
OMM3081b_TAP1 Omy_2:42.9 , DRE_16:59015165:59015816,OLA_16:26300042:26300182,TNI_scaffold6040:78,TRU_scaffold170:286,119:291,216
101.8 OMM1391a
109.2 OMM5062a Omy_2:39.6, OLA_16:17690960:17694198
OMM5225b Omy_2:18.5
OMM1263b Omy_2:78.6
113.5 OMM1624a Omy_2:18.5
OMM5067 DRE_16:7964366:7967180
OMM1138
115.4 OMM5121 TNI_unrandom:110699137:110700375
116.1 OMM1137
120.4 OMM5109
OMM1336
122.2 OMM5142a Omy_2:42.9, DRE_16:17262800:17276443,TNI_8:8797308:8800761,TRU_scaffold466:40222:43827
127.1 OMM1778b Omy_2:11.8
OMM1198
142.2 OMM1016B

Omy4

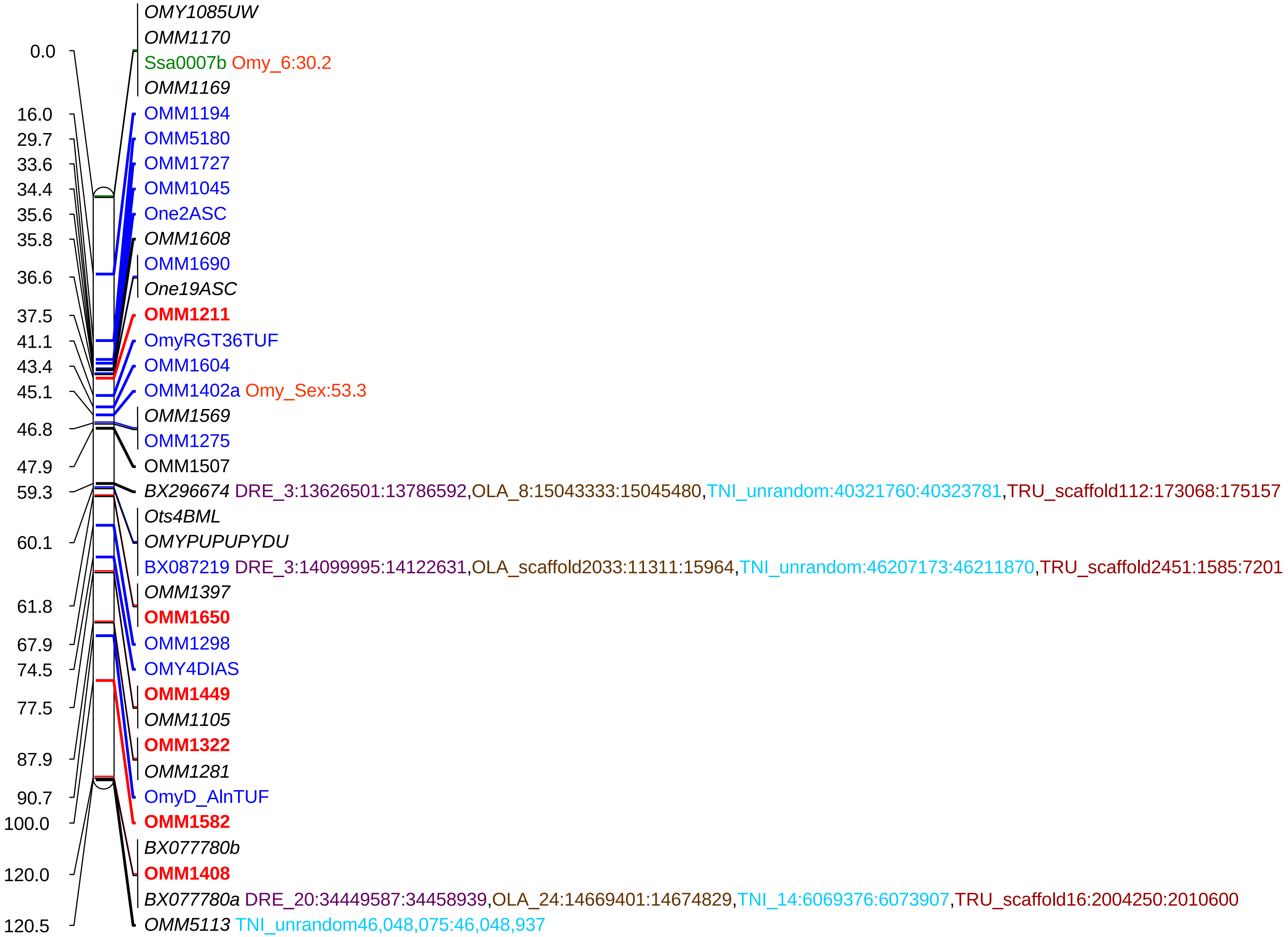

0.0
16.0
29.7
33.6
34.4
35.6
35.8
36.6
37.5
41.1
43.4
45.1
46.8
47.9
59.3
60.1
61.8
67.9
74.5
77.5
87.9
90.7
100.0
120.0
120.5
OMY1085UW
OMM1170
Ssa0007b Omy_6:30.2
OMM1169
OMM1194
OMM5180
OMM1727
OMM1045
One2ASC
OMM1608
OMM1690
One19ASC
OMM1211
OmyRGT36TUF
OMM1604
OMM1402a Omy_Sex:53.3
OMM1569
OMM1275
OMM1507
BX296674 DRE_3:13626501:13786592,OLA_8:15043333:15045480,TNI_unrandom:40321760:40323781,TRU_scaffold112:173068:175157
Ots4BML
OMYPUPUPYDU
BX087219 DRE_3:14099995:14122631,OLA_scaffold2033:11311:15964,TNI_unrandom:46207173:46211870,TRU_scaffold2451:1585:7201
OMM1397
OMM1650
OMM1298
OMY4DIAS
OMM1449
OMM1105
OMM1322
OMM1281
OmyD_AlnTUF
OMM1582
BX077780b
OMM1408
BX077780a DRE_20:34449587:34458939,OLA_24:14669401:14674829,TNI_14:6069376:6073907,TRU_scaffold16:2004250:2010600
OMM5113 TNI_unrandom46,048,075:46,048,937

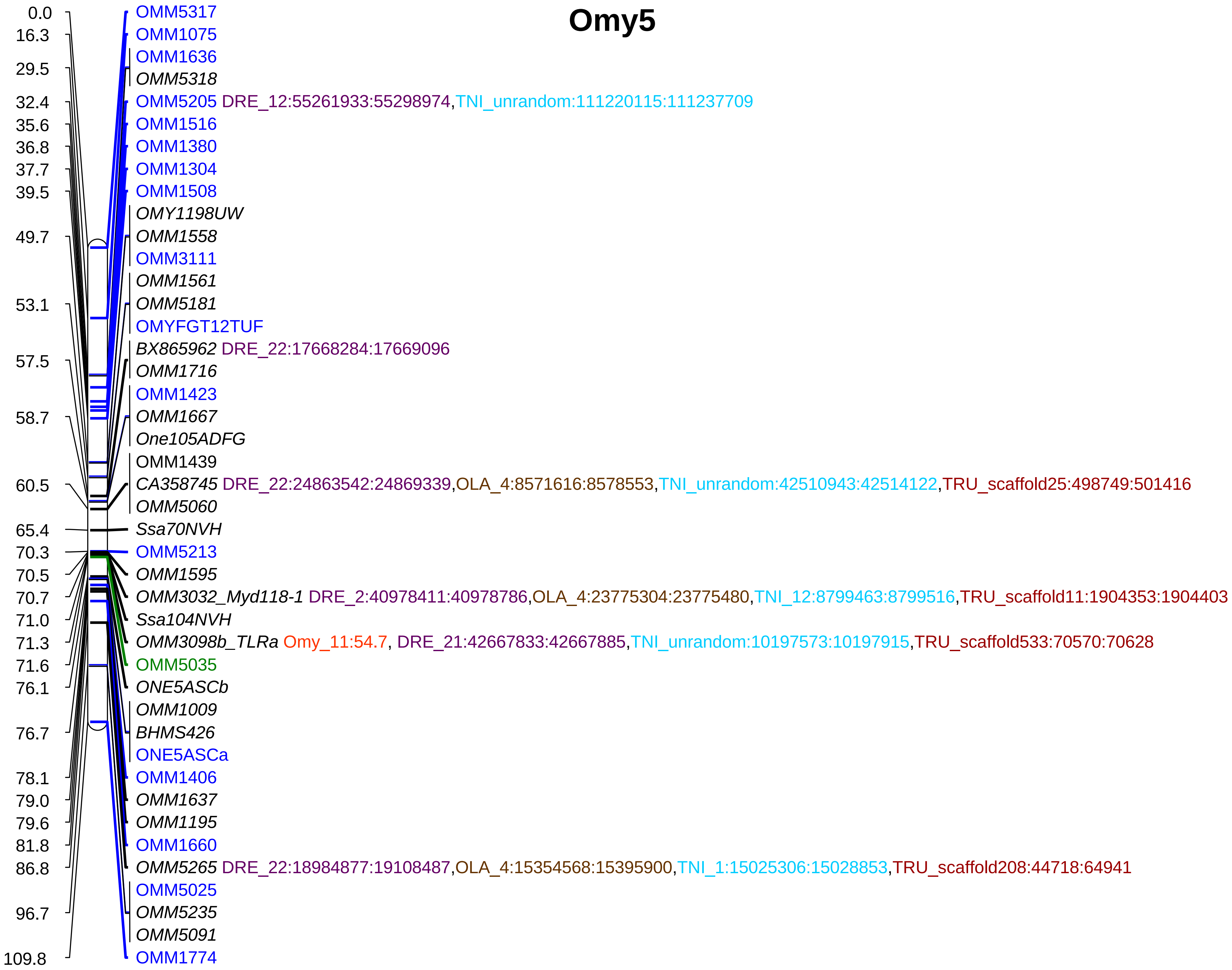
Omy5
0.0
16.3
29.5
32.4
35.6
36.8
37.7
39.5
49.7
53.1
57.5
58.7
60.5
65.4
70.3
70.5
70.7
71.0
71.3
71.6
76.1
76.7
78.1
79.0
79.6
81.8
86.8
96.7
109.8
OMM5317
OMM1075
OMM1636
OMM5318
OMM5205 DRE_12:55261933:55298974,TNI_unrandom:111220115:111237709
OMM1516
OMM1380
OMM1304
OMM1508
OMY1198UW
OMM1558
OMM3111
OMM1561
OMM5181
OMYFGT12TUF
BX865962 DRE_22:17668284:17669096
OMM1716
OMM1423
OMM1667
One105ADFG
OMM1439
CA358745 DRE_22:24863542:24869339,OLA_4:8571616:8578553,TNI_unrandom:42510943:42514122,TRU_scaffold25:498749:501416
OMM5060
Ssa70NVH
OMM5213
OMM1595
OMM3032_Myd118-1 DRE_2:40978411:40978786,OLA_4:23775304:23775480,TNI_12:8799463:8799516,TRU_scaffold11:1904353:1904403
Ssa104NVH
OMM3098b_TLRa Omy_11:54.7, DRE_21:42667833:42667885,TNI_unrandom:10197573:10197915,TRU_scaffold533:70570:70628
OMM5035
ONE5ASCb
OMM1009
BHMS426
ONE5ASCa
OMM1406
OMM1637
OMM1195
OMM1660
OMM5265 DRE_22:18984877:19108487,OLA_4:15354568:15395900,TNI_1:15025306:15028853,TRU_scaffold208:44718:64941
OMM5025
OMM5235
OMM5091
OMM1774

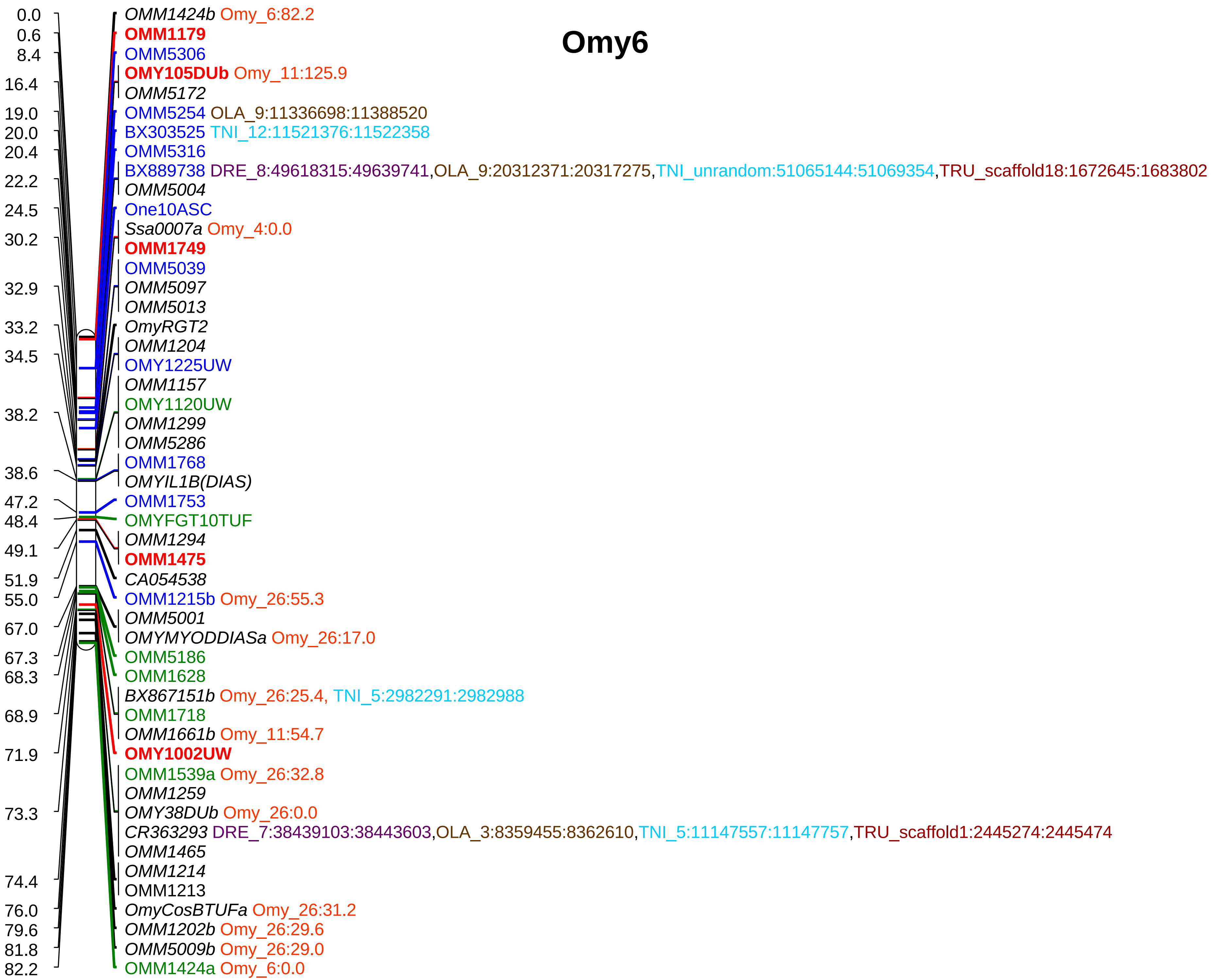

Omy6
0.0 OMM1424b Omy_6:82.2
0.6 OMM1179
8.4 OMM5306
16.4 OMY105DUb Omy_11:125.9
OMM5172
19.0 OMM5254 OLA_9:11336698:11388520
20.0 BX303525 TNI_12:11521376:11522358
20.4 OMM5316
22.2 BX889738 DRE_8:49618315:49639741,OLA_9:20312371:20317275,TNI_unrandom:51065144:51069354,TRU_scaffold18:1672645:1683802
OMM5004
24.5 One10ASC
30.2 Ssa0007a Omy_4:0.0
OMM1749
OMM5039
32.9 OMM5097
OMM5013
33.2 OmyRGT2
34.5 OMM1204
OMY1225UW
OMM1157
38.2 OMY1120UW
OMM1299
OMM5286
38.6 OMM1768
OMYIL1B(DIAS)
47.2 OMM1753
48.4 OMYFGT10TUF
49.1 OMM1294
OMM1475
51.9 CA054538
55.0 OMM1215b Omy_26:55.3
67.0 OMM5001
OMYMYODDIASa Omy_26:17.0
67.3 OMM5186
68.3 OMM1628
BX867151b Omy_26:25.4, TNI_5:2982291:2982988
68.9 OMM1718
OMM1661b Omy_11:54.7
71.9 OMY1002UW
OMM1539a Omy_26:32.8
OMM1259
73.3 OMY38DUb Omy_26:0.0
CR363293 DRE_7:38439103:38443603,OLA_3:8359455:8362610,TNI_5:11147557:11147757,TRU_scaffold1:2445274:2445474
OMM1465
74.4 OMM1214
OMM1213
76.0 OmyCosBTUFa Omy_26:31.2
79.6 OMM1202b Omy_26:29.6
81.8 OMM5009b Omy_26:29.0
82.2 OMM1424a Omy_6:0.0

Omy7

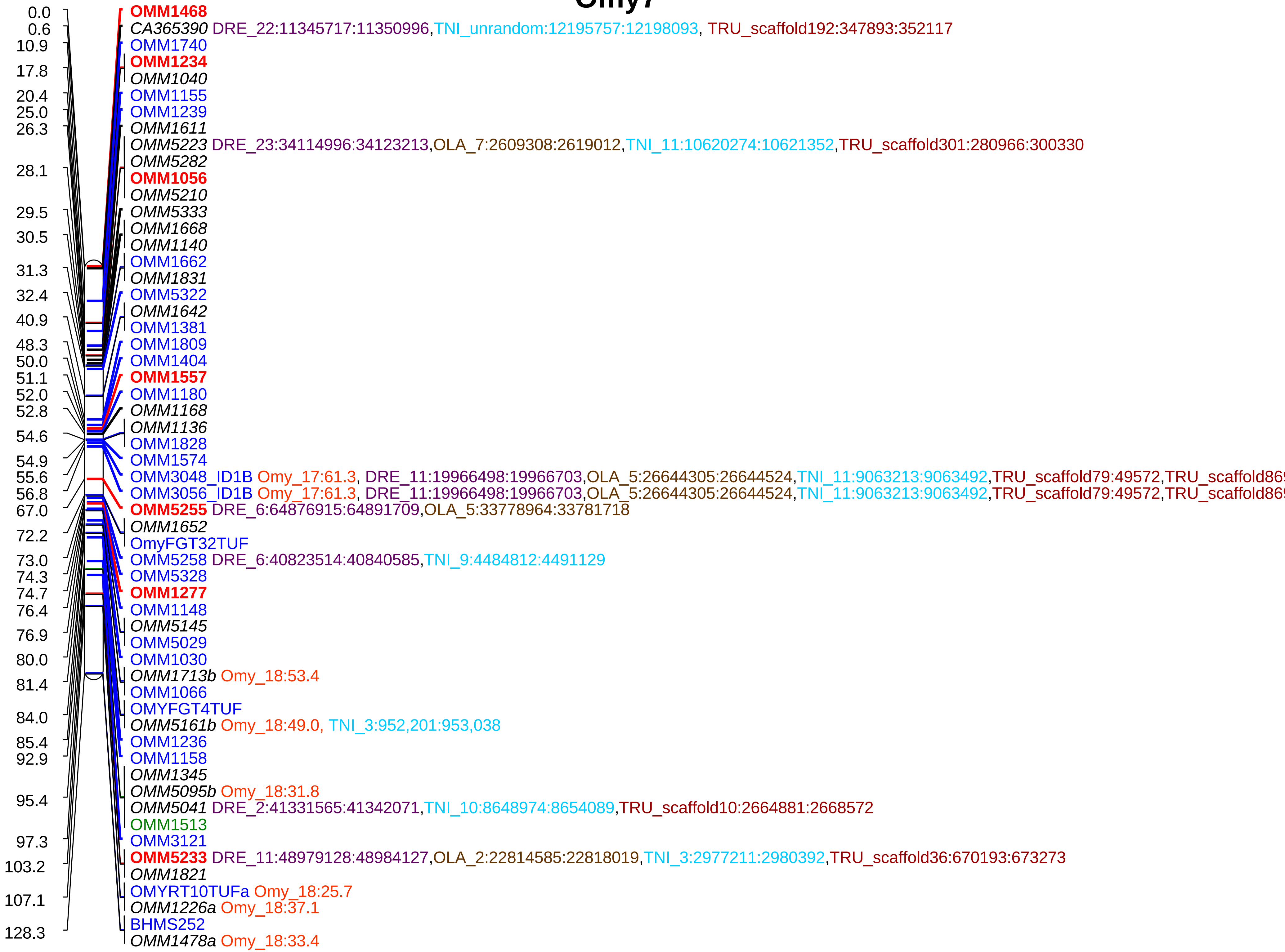
0.0
0.6
10.9
17.8
20.4
25.0
26.3
28.1
29.5
30.5
31.3
32.4
40.9
48.3
50.0
51.1
52.0
52.8
54.6
54.9
55.6
56.8
67.0
72.2
73.0
74.3
74.7
76.4
76.9
80.0
81.4
84.0
85.4
92.9
95.4
97.3
103.2
107.1
128.3
OMM1468
CA365390 DRE_22:11345717:11350996,TNI_unrandom:12195757:12198093, TRU_scaffold192:347893:352117
OMM1740
OMM1234
OMM1040
OMM1155
OMM1239
OMM1611
OMM5223 DRE_23:34114996:34123213,OLA_7:2609308:2619012,TNI_11:10620274:10621352,TRU_scaffold301:280966:300330
OMM5282
OMM1056
OMM5210
OMM5333
OMM1668
OMM1140
OMM1662
OMM1831
OMM5322
OMM1642
OMM1381
OMM1809
OMM1404
OMM1557
OMM1180
OMM1168
OMM1136
OMM1828
OMM1574
OMM3048_ID1B Omy_17:61.3, DRE_11:19966498:19966703,OLA_5:26644305:26644524,TNI_11:9063213:9063492,TRU_scaffold79:49572,TRU_scaffold86
OMM3056_ID1B Omy_17:61.3, DRE_11:19966498:19966703,OLA_5:26644305:26644524,TNI_11:9063213:9063492,TRU_scaffold79:49572,TRU_scaffold86
OMM5255 DRE_6:64876915:64891709,OLA_5:33778964:33781718
OMM1652
OmyFGT32TUF
OMM5258 DRE_6:40823514:40840585,TNI_9:4484812:4491129
OMM5328
OMM1277
OMM1148
OMM5145
OMM5029
OMM1030
OMM1713b Omy_18:53.4
OMM1066
OMYFGT4TUF
OMM5161b Omy_18:49.0, TNI_3:952,201:953,038
OMM1236
OMM1158
OMM1345
OMM5095b Omy_18:31.8
OMM5041 DRE_2:41331565:41342071,TNI_10:8648974:8654089,TRU_scaffold10:2664881:2668572
OMM1513
OMM3121
OMM5233 DRE_11:48979128:48984127,OLA_2:22814585:22818019,TNI_3:2977211:2980392,TRU_scaffold36:670193:673273
OMM1821
OMYRT10TUFa Omy_18:25.7
OMM1226a Omy_18:37.1
BHMS252
OMM1478a Omy_18:33.4

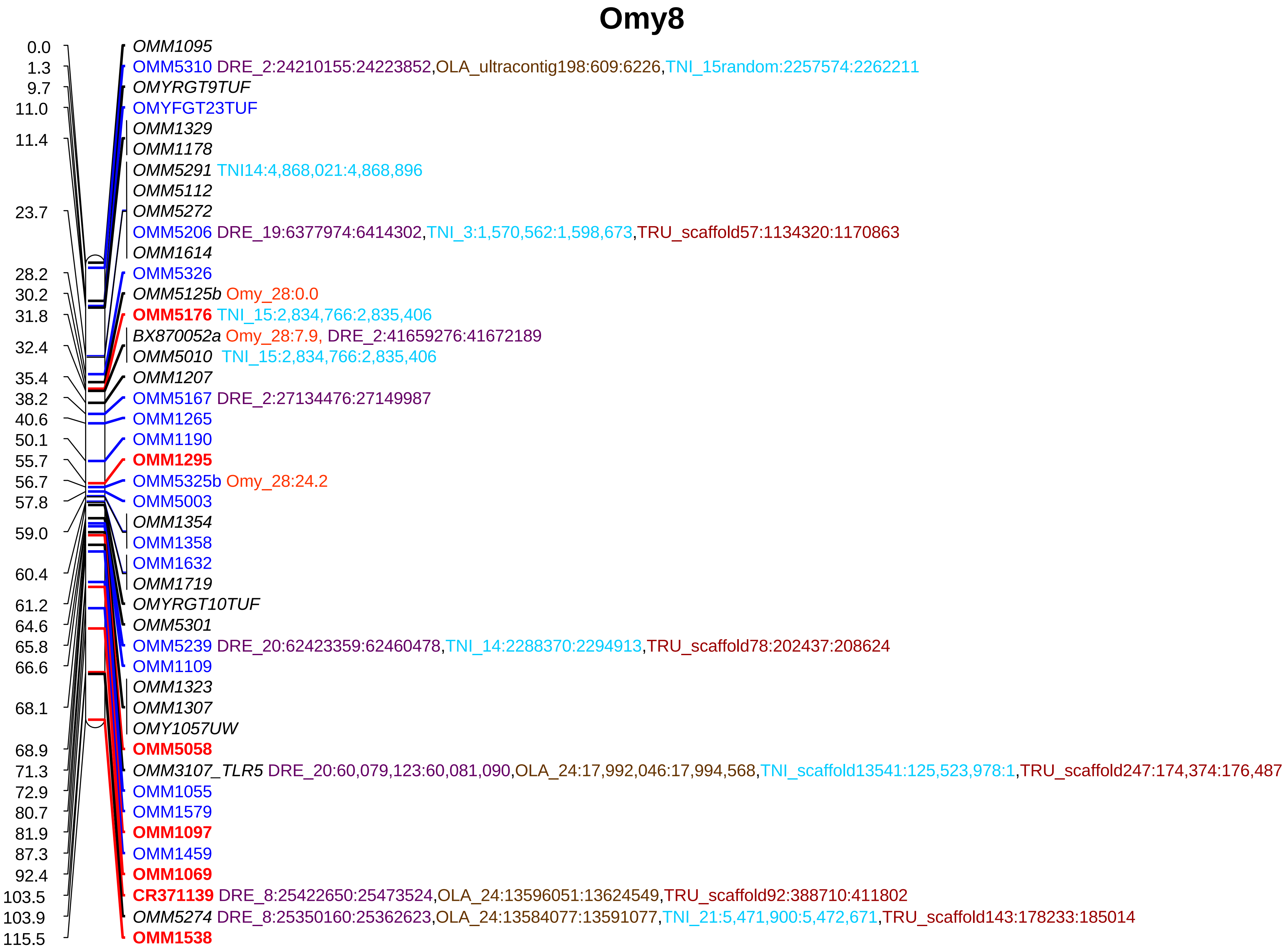

Omy8
0.0
1.3
9.7
11.0
11.4
23.7
28.2
30.2
31.8
32.4
35.4
38.2
40.6
50.1
55.7
56.7
57.8
59.0
60.4
61.2
64.6
65.8
66.6
68.1
68.9
71.3
72.9
80.7
81.9
87.3
92.4
103.5
103.9
115.5
OMM1095
OMM5310 DRE_2:24210155:24223852,OLA_ultracontig198:609:6226,TNI_15random:2257574:2262211
OMYRGT9TUF
OMYFGT23TUF
OMM1329
OMM1178
OMM5291 TNI14:4,868,021:4,868,896
OMM5112
OMM5272
OMM5206 DRE_19:6377974:6414302,TNI_3:1,570,562:1,598,673,TRU_scaffold57:1134320:1170863
OMM1614
OMM5326
OMM5125b Omy_28:0.0
OMM5176 TNI_15:2,834,766:2,835,406
BX870052a Omy_28:7.9, DRE_2:41659276:41672189
OMM5010 TNI_15:2,834,766:2,835,406
OMM1207
OMM5167 DRE_2:27134476:27149987
OMM1265
OMM1190
OMM1295
OMM5325b Omy_28:24.2
OMM5003
OMM1354
OMM1358
OMM1632
OMM1719
OMYRGT10TUF
OMM5301
OMM5239 DRE_20:62423359:62460478,TNI_14:2288370:2294913,TRU_scaffold78:202437:208624
OMM1109
OMM1323
OMM1307
OMY1057UW
OMM5058
OMM3107_TLR5 DRE_20:60,079,123:60,081,090,OLA_24:17,992,046:17,994,568,TNI_scaffold13541:125,523,978:1,TRU_scaffold247:174,374:176,487
OMM1055
OMM1579
OMM1097
OMM1459
OMM1069
CR371139 DRE_8:25422650:25473524,OLA_24:13596051:13624549,TRU_scaffold92:388710:411802
OMM5274 DRE_8:25350160:25362623,OLA_24:13584077:13591077,TNI_21:5,471,900:5,472,671,TRU_scaffold143:178233:185014
OMM1538

Omy9

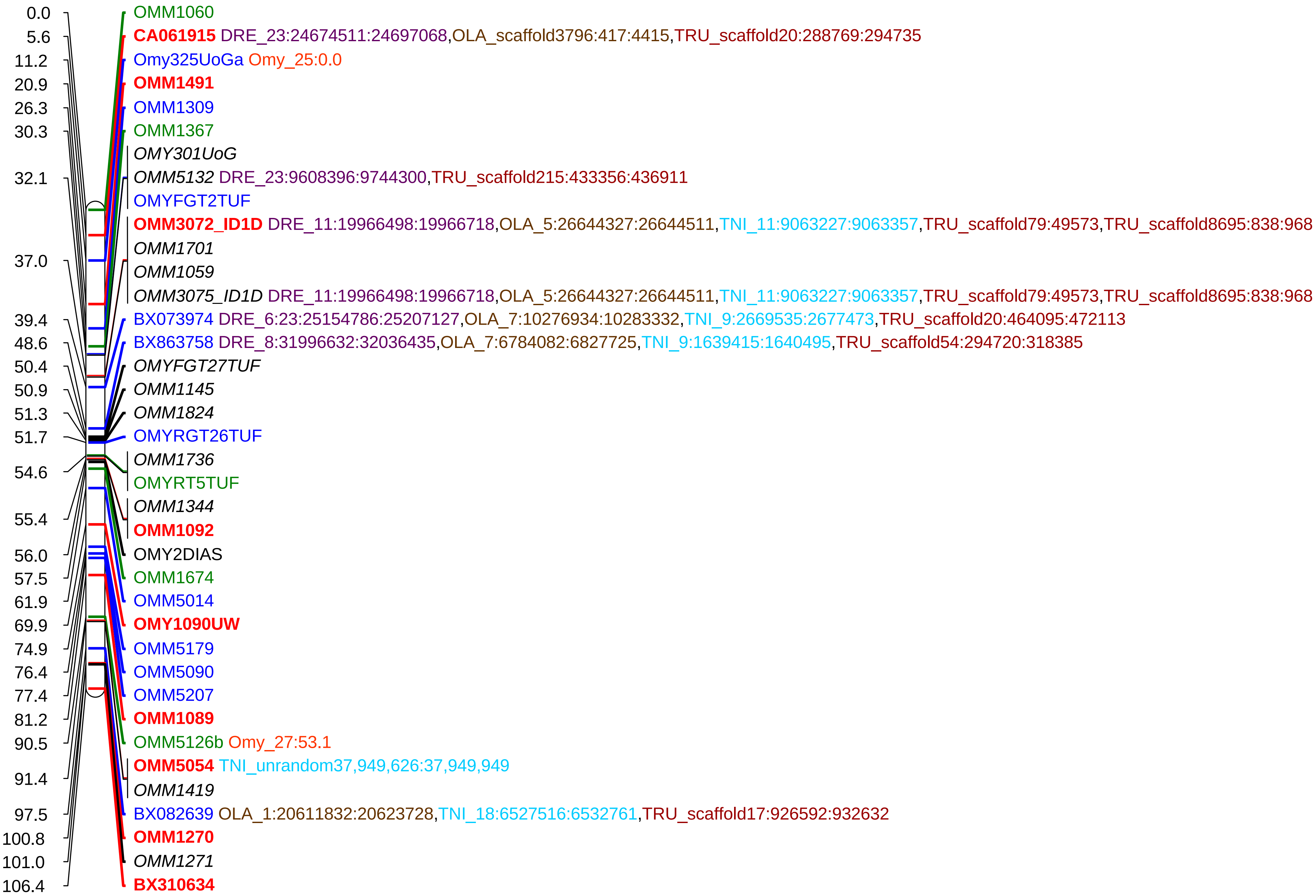
0.0
5.6
11.2
20.9
26.3
30.3
32.1
37.0
39.4
48.6
50.4
50.9
51.3
51.7
54.6
55.4
56.0
57.5
61.9
69.9
74.9
76.4
77.4
81.2
90.5
91.4
97.5
100.8
101.0
106.4
OMM1060
CA061915 DRE_23:24674511:24697068,OLA_scaffold3796:417:4415,TRU_scaffold20:288769:294735
Omy325UoGa Omy_25:0.0
OMM1491
OMM1309
OMM1367
OMY301UoG
OMM5132 DRE_23:9608396:9744300,TRU_scaffold215:433356:436911
OMYFGT2TUF
OMM3072_ID1D DRE_11:19966498:19966718,OLA_5:26644327:26644511,TNI_11:9063227:9063357,TRU_scaffold79:49573,TRU_scaffold8695:838:968
OMM1701
OMM1059
OMM3075_ID1D DRE_11:19966498:19966718,OLA_5:26644327:26644511,TNI_11:9063227:9063357,TRU_scaffold79:49573,TRU_scaffold8695:838:968
BX073974 DRE_6:23:25154786:25207127,OLA_7:10276934:10283332,TNI_9:2669535:2677473,TRU_scaffold20:464095:472113
BX863758 DRE_8:31996632:32036435,OLA_7:6784082:6827725,TNI_9:1639415:1640495,TRU_scaffold54:294720:318385
OMYFGT27TUF
OMM1145
OMM1824
OMYRGT26TUF
OMM1736
OMYRT5TUF
OMM1344
OMM1092
OMY2DIAS
OMM1674
OMM5014
OMY1090UW
OMM5179
OMM5090
OMM5207
OMM1089
OMM5126b Omy_27:53.1
OMM5054 TNI_unrandom37,949,626:37,949,949
OMM1419
BX082639 OLA_1:20611832:20623728,TNI_18:6527516:6532761,TRU_scaffold17:926592:932632
OMM1270
OMM1271
BX310634

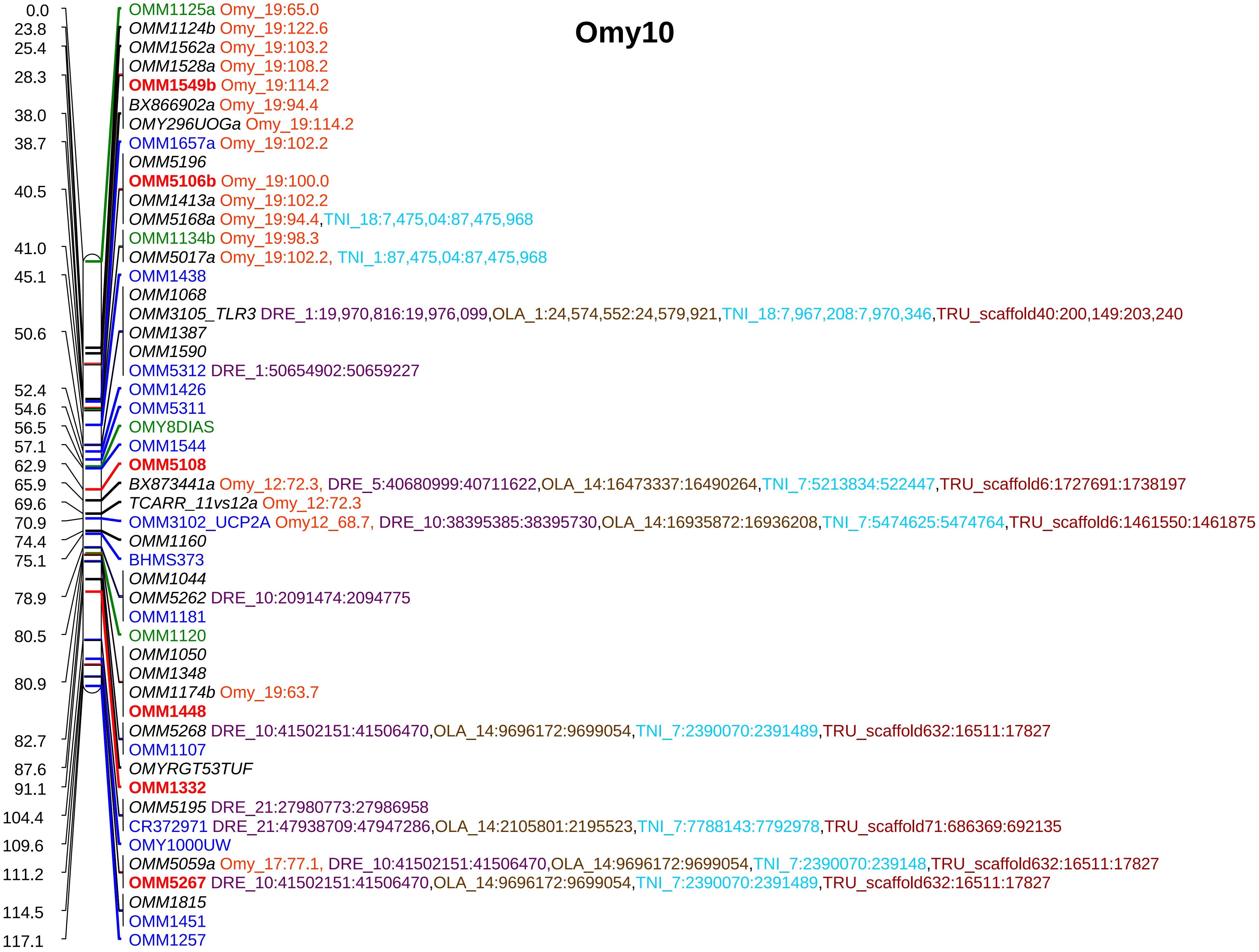
Omy10
0.0
23.8
25.4
28.3
38.0
38.7
40.5
41.0
45.1
50.6
52.4
54.6
56.5
57.1
62.9
65.9
69.6
70.9
74.4
75.1
78.9
80.5
80.9
82.7
87.6
91.1
104.4
109.6
111.2
114.5
117.1
OMM1125a Omy_19:65.0
OMM1124b Omy_19:122.6
OMM1562a Omy_19:103.2
OMM1528a Omy_19:108.2
OMM1549b Omy_19:114.2
BX866902a Omy_19:94.4
OMY296UOGa Omy_19:114.2
OMM1657a Omy_19:102.2
OMM5196
OMM5106b Omy_19:100.0
OMM1413a Omy_19:102.2
OMM5168a Omy_19:94.4,TNI_18:7,475,04:87,475,968
OMM1134b Omy_19:98.3
OMM5017a Omy_19:102.2, TNI_1:87,475,04:87,475,968
OMM1438
OMM1068
OMM3105_TLR3 DRE_1:19,970,816:19,976,099,OLA_1:24,574,552:24,579,921,TNI_18:7,967,208:7,970,346,TRU_scaffold40:200,149:203,240
OMM1387
OMM1590
OMM5312 DRE_1:50654902:50659227
OMM1426
OMM5311
OMY8DIAS
OMM1544
OMM5108
BX873441a Omy_12:72.3, DRE_5:40680999:40711622,OLA_14:16473337:16490264,TNI_7:5213834:522447,TRU_scaffold6:1727691:1738197
TCARR_11vs12a Omy_12:72.3
OMM3102_UCP2A Omy12_68.7, DRE_10:38395385:38395730,OLA_14:16935872:16936208,TNI_7:5474625:5474764,TRU_scaffold6:1461550:1461875
OMM1160
BHMS373
OMM1044
OMM5262 DRE_10:2091474:2094775
OMM1181
OMM1120
OMM1050
OMM1348
OMM1174b Omy_19:63.7
OMM1448
OMM5268 DRE_10:41502151:41506470,OLA_14:9696172:9699054,TNI_7:2390070:2391489,TRU_scaffold632:16511:17827
OMM1107
OMYRGT53TUF
OMM1332
OMM5195 DRE_21:27980773:27986958
CR372971 DRE_21:47938709:47947286,OLA_14:2105801:2195523,TNI_7:7788143:7792978,TRU_scaffold71:686369:692135
OMY1000UW
OMM5059a Omy_17:77.1, DRE_10:41502151:41506470,OLA_14:9696172:9699054,TNI_7:2390070:239148,TRU_scaffold632:16511:17827
OMM5267 DRE_10:41502151:41506470,OLA_14:9696172:9699054,TNI_7:2390070:2391489,TRU_scaffold632:16511:17827
OMM1815
OMM1451
OMM1257

Omy11

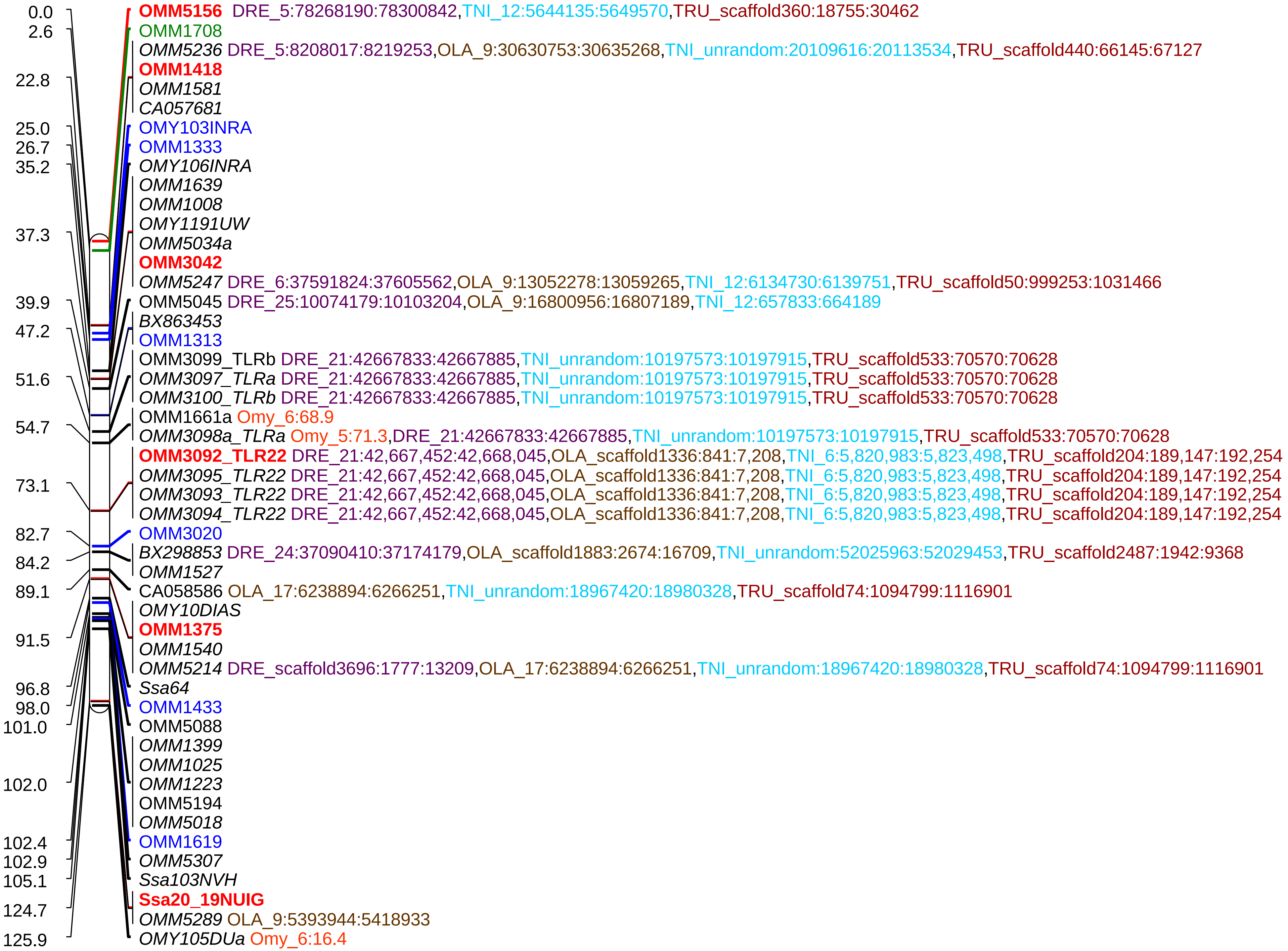
0.0
2.6
22.8
25.0
26.7
35.2
37.3
39.9
47.2
51.6
54.7
73.1
82.7
84.2
89.1
91.5
96.8
98.0
101.0
102.0
102.4
102.9
105.1
124.7
125.9
OMM5156 DRE_5:78268190:78300842,TNI_12:5644135:5649570,TRU_scaffold360:18755:30462
OMM1708
OMM5236 DRE_5:8208017:8219253,OLA_9:30630753:30635268,TNI_unrandom:20109616:20113534,TRU_scaffold440:66145:67127
OMM1418
OMM1581
CA057681
OMY103INRA
OMM1333
OMY106INRA
OMM1639
OMM1008
OMY1191UW
OMM5034a
OMM3042
OMM5247 DRE_6:37591824:37605562,OLA_9:13052278:13059265,TNI_12:6134730:6139751,TRU_scaffold50:999253:1031466
OMM5045 DRE_25:10074179:10103204,OLA_9:16800956:16807189,TNI_12:657833:664189
BX863453
OMM1313
OMM3099_TLRb DRE_21:42667833:42667885,TNI_unrandom:10197573:10197915,TRU_scaffold533:70570:70628
OMM3097_TLRa DRE_21:42667833:42667885,TNI_unrandom:10197573:10197915,TRU_scaffold533:70570:70628
OMM3100_TLRb DRE_21:42667833:42667885,TNI_unrandom:10197573:10197915,TRU_scaffold533:70570:70628
OMM1661a Omy_6:68.9
OMM3098a_TLRa Omy_5:71.3,DRE_21:42667833:42667885,TNI_unrandom:10197573:10197915,TRU_scaffold533:70570:70628
OMM3092_TLR22 DRE_21:42,667,452:42,668,045,OLA_scaffold1336:841:7,208,TNI_6:5,820,983:5,823,498,TRU_scaffold204:189,147:192,254
OMM3095_TLR22 DRE_21:42,667,452:42,668,045,OLA_scaffold1336:841:7,208,TNI_6:5,820,983:5,823,498,TRU_scaffold204:189,147:192,254
OMM3093_TLR22 DRE_21:42,667,452:42,668,045,OLA_scaffold1336:841:7,208,TNI_6:5,820,983:5,823,498,TRU_scaffold204:189,147:192,254
OMM3094_TLR22 DRE_21:42,667,452:42,668,045,OLA_scaffold1336:841:7,208,TNI_6:5,820,983:5,823,498,TRU_scaffold204:189,147:192,254
OMM3020
BX298853 DRE_24:37090410:37174179,OLA_scaffold1883:2674:16709,TNI_unrandom:52025963:52029453,TRU_scaffold2487:1942:9368
OMM1527
CA058586 OLA_17:6238894:6266251,TNI_unrandom:18967420:18980328,TRU_scaffold74:1094799:1116901
OMY10DIAS
OMM1375
OMM1540
OMM5214 DRE_scaffold3696:1777:13209,OLA_17:6238894:6266251,TNI_unrandom:18967420:18980328,TRU_scaffold74:1094799:1116901
Ssa64
OMM1433
OMM5088
OMM1399
OMM1025
OMM1223
OMM5194
OMM5018
OMM1619
OMM5307
Ssa103NVH
Ssa20_19NUIG
OMM5289 OLA_9:5393944:5418933
OMY105DUa Omy_6:16.4

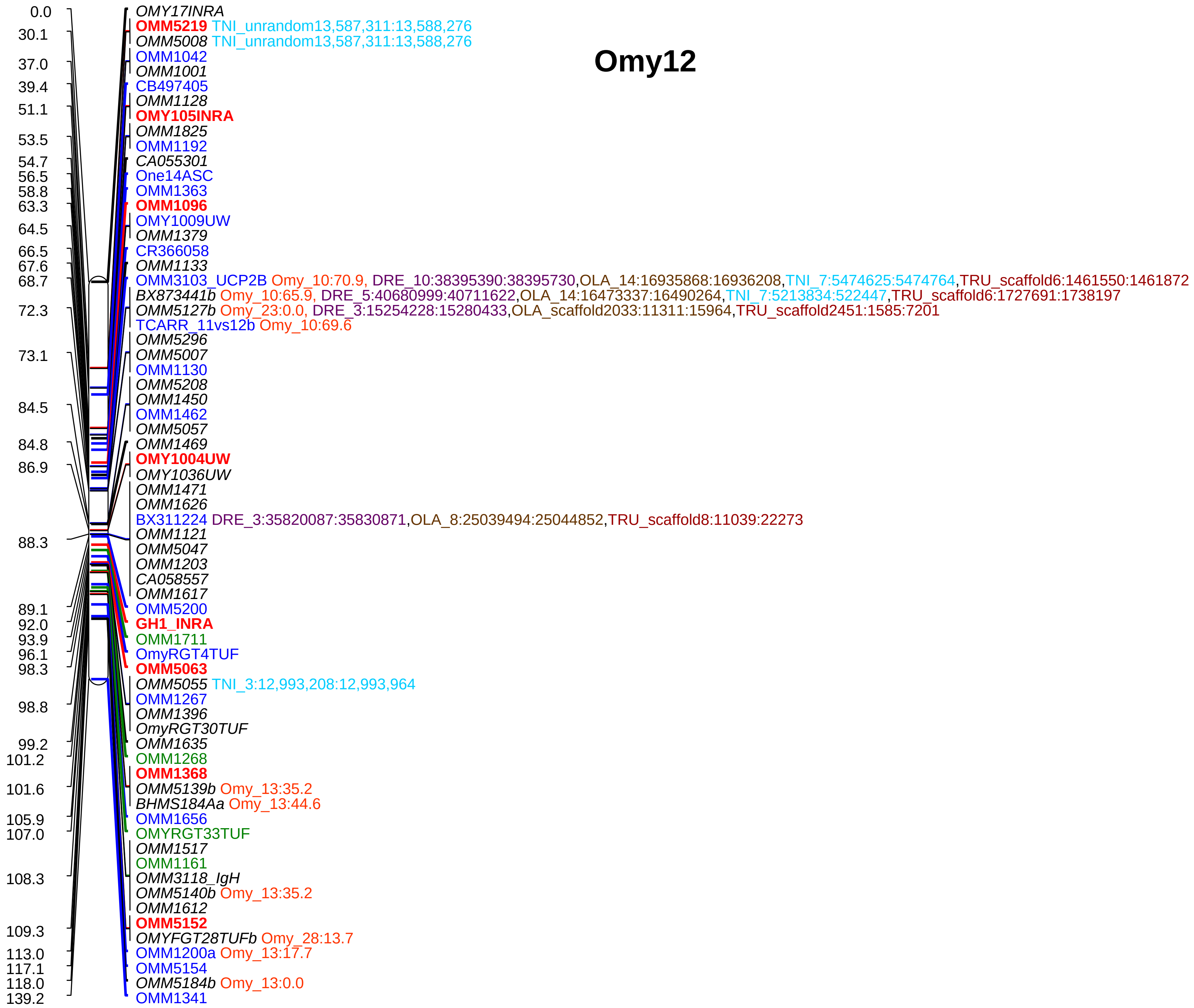
Omy12
0.0
30.1
37.0
39.4
51.1
53.5
54.7
56.5
58.8
63.3
64.5
66.5
67.6
68.7
72.3
73.1
84.5
84.8
86.9
88.3
89.1
92.0
93.9
96.1
98.3
98.8
99.2
101.2
101.6
105.9
107.0
108.3
109.3
113.0
117.1
118.0
139.2
OMY17INRA
OMM5219 TNI_unrandom13,587,311:13,588,276
OMM5008 TNI_unrandom13,587,311:13,588,276
OMM1042
OMM1001
CB497405
OMM1128
OMY105INRA
OMM1825
OMM1192
CA055301
One14ASC
OMM1363
OMM1096
OMY1009UW
OMM1379
CR366058
OMM1133
OMM3103_UCP2B Omy_10:70.9, DRE_10:38395390:38395730,OLA_14:16935868:16936208,TNI_7:5474625:5474764,TRU_scaffold6:1461550:1461872
BX873441b Omy_10:65.9, DRE_5:40680999:40711622,OLA_14:16473337:16490264,TNI_7:5213834:522447,TRU_scaffold6:1727691:1738197
OMM5127b Omy_23:0.0, DRE_3:15254228:15280433,OLA_scaffold2033:11311:15964,TRU_scaffold2451:1585:7201
TCARR_11vs12b Omy_10:69.6
OMM5296
OMM5007
OMM1130
OMM5208
OMM1450
OMM1462
OMM5057
OMM1469
OMY1004UW
OMY1036UW
OMM1471
OMM1626
BX311224 DRE_3:35820087:35830871,OLA_8:25039494:25044852,TRU_scaffold8:11039:22273
OMM1121
OMM5047
OMM1203
CA058557
OMM1617
OMM5200
GH1_INRA
OMM1711
OmyRGT4TUF
OMM5063
OMM5055 TNI_3:12,993,208:12,993,964
OMM1267
OMM1396
OmyRGT30TUF
OMM1635
OMM1268
OMM1368
OMM5139b Omy_13:35.2
BHMS184Aa Omy_13:44.6
OMM1656
OMYRGT33TUF
OMM1517
OMM1161
OMM3118_IgH
OMM5140b Omy_13:35.2
OMM1612
OMM5152
OMYFGT28TUFb Omy_28:13.7
OMM1200a Omy_13:17.7
OMM5154
OMM5184b Omy_13:0.0
OMM1341

Omy13

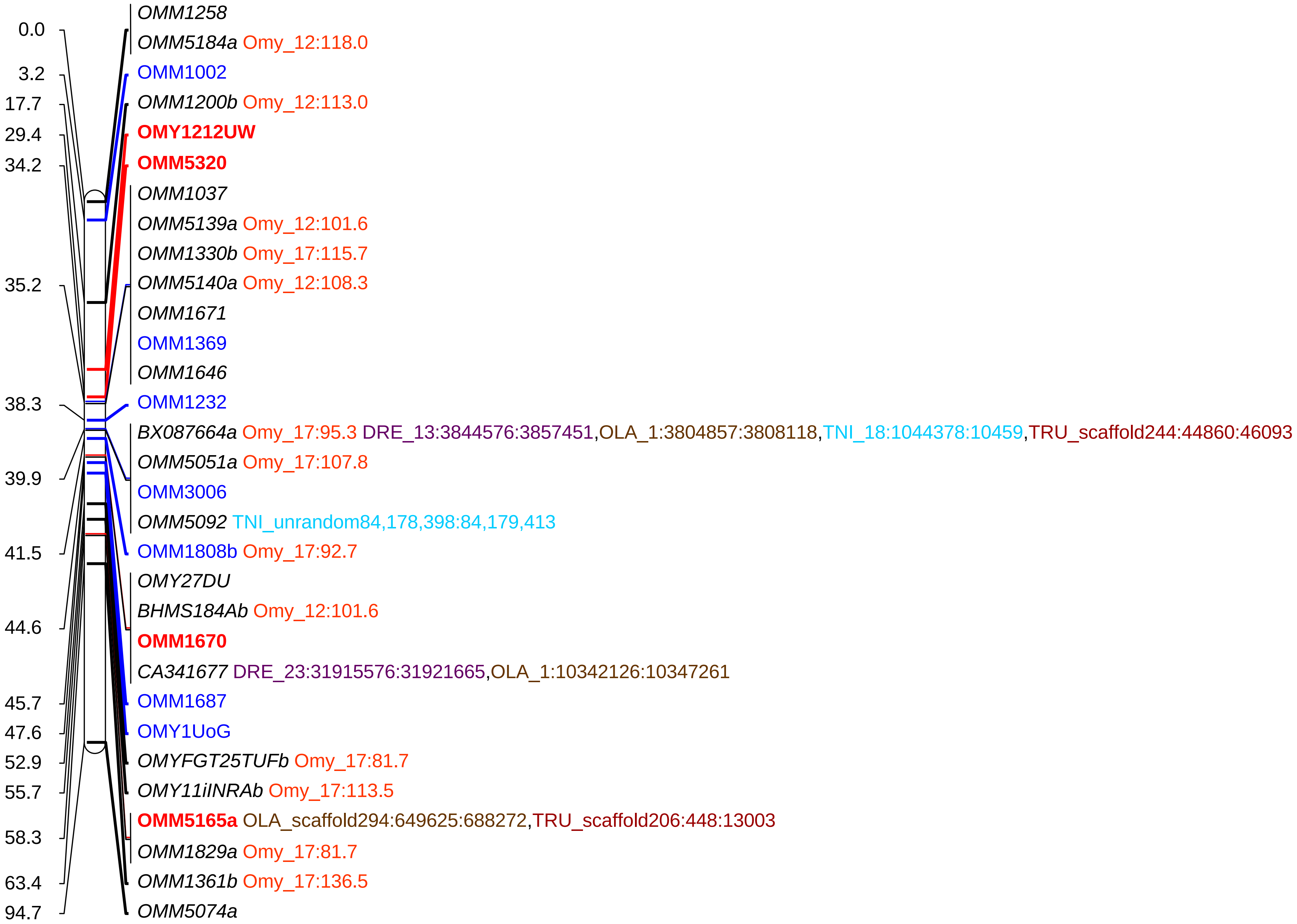

0.0
3.2
17.7
29.4
34.2
35.2
38.3
39.9
41.5
44.6
45.7
47.6
52.9
55.7
58.3
63.4
94.7
OMM1258
OMM5184a Omy_12:118.0
OMM1002
OMM1200b Omy_12:113.0
OMY1212UW
OMM5320
OMM1037
OMM5139a Omy_12:101.6
OMM1330b Omy_17:115.7
OMM5140a Omy_12:108.3
OMM1671
OMM1369
OMM1646
OMM1232
BX087664a Omy_17:95.3 DRE_13:3844576:3857451,OLA_1:3804857:3808118,TNI_18:1044378:10459,TRU_scaffold244:44860:46093
OMM5051a Omy_17:107.8
OMM3006
OMM5092 TNI_unrandom84,178,398:84,179,413
OMM1808b Omy_17:92.7
OMY27DU
BHMS184Ab Omy_12:101.6
OMM1670
CA341677 DRE_23:31915576:31921665,OLA_1:10342126:10347261
OMM1687
OMY1UoG
OMYFGT25TUFb Omy_17:81.7
OMY11iINRAb Omy_17:113.5
OMM5165a OLA_scaffold294:649625:688272,TRU_scaffold206:448:13003
OMM1829a Omy_17:81.7
OMM1361b Omy_17:136.5
OMM5074a

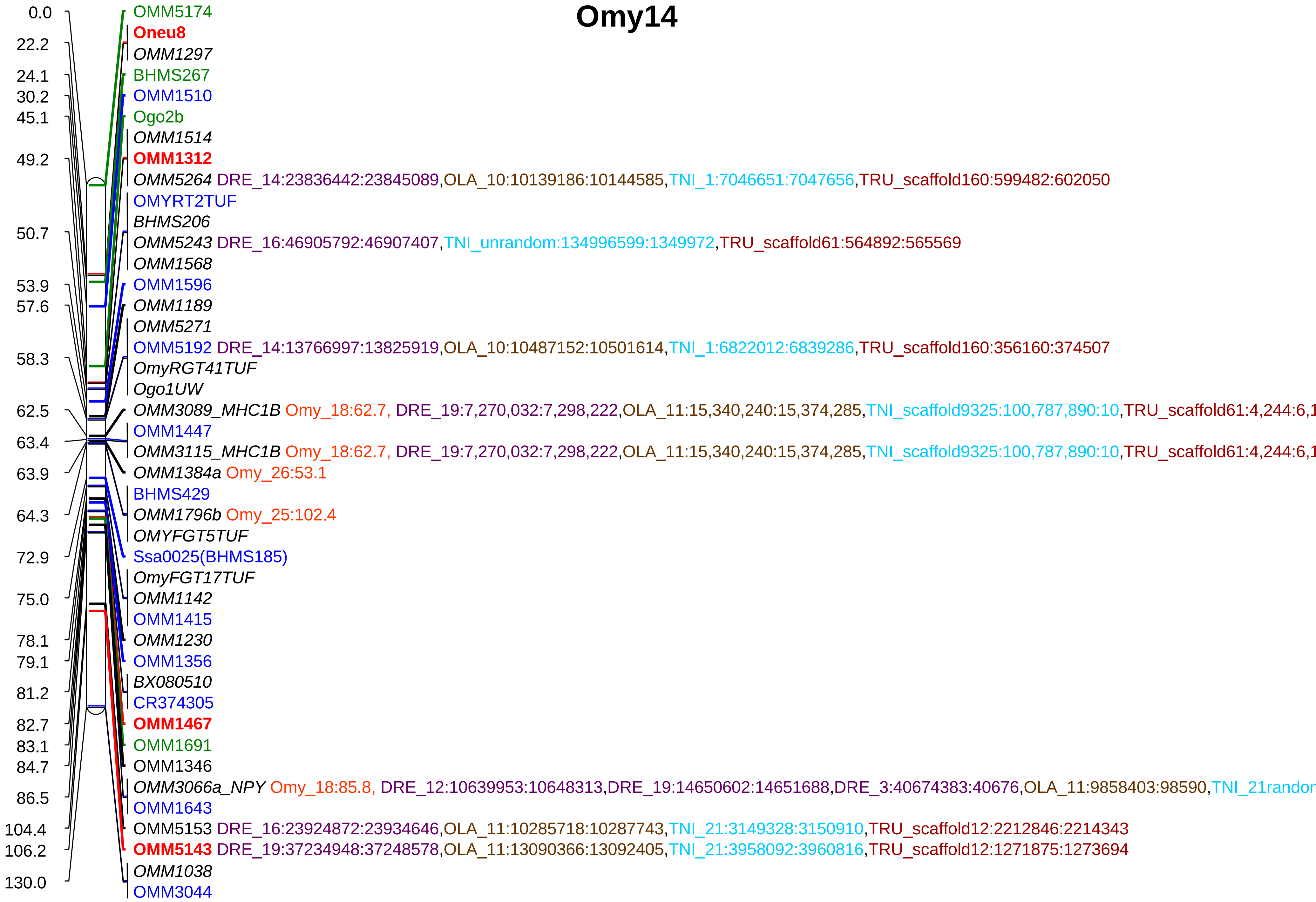
Omy14
0.0
22.2
24.1
30.2
45.1
49.2
50.7
53.9
57.6
58.3
62.5
63.4
63.9
64.3
72.9
75.0
78.1
79.1
81.2
82.7
83.1
84.7
86.5
104.4
106.2
130.0
OMM5174
Oneu8
OMM1297
BHMS267
OMM1510
Ogo2b
OMM1514
OMM1312
OMM5264 DRE_14:23836442:23845089,OLA_10:10139186:10144585,TNI_1:7046651:7047656,TRU_scaffold160:599482:602050
OMYRT2TUF
BHMS206
OMM5243 DRE_16:46905792:46907407,TNI_unrandom:134996599:1349972,TRU_scaffold61:564892:565569
OMM1568
OMM1596
OMM1189
OMM5271
OMM5192 DRE_14:13766997:13825919,OLA_10:10487152:10501614,TNI_1:6822012:6839286,TRU_scaffold160:356160:374507
OmyRGT41TUF
Ogo1UW
OMM3089_MHC1B Omy_18:62.7, DRE_19:7,270,032:7,298,222,OLA_11:15,340,240:15,374,285,TNI_scaffold9325:100,787,890:10,TRU_scaffold61:4,244:6,1
OMM1447
OMM3115_MHC1B Omy_18:62.7, DRE_19:7,270,032:7,298,222,OLA_11:15,340,240:15,374,285,TNI_scaffold9325:100,787,890:10,TRU_scaffold61:4,244:6,1
OMM1384a Omy_26:53.1
BHMS429
OMM1796b Omy_25:102.4
OMYFGT5TUF
Ssa0025(BHMS185)
OmyFGT17TUF
OMM1142
OMM1415
OMM1230
OMM1356
BX080510
CR374305
OMM1467
OMM1691
OMM1346
OMM3066a_NPY Omy_18:85.8, DRE_12:10639953:10648313,DRE_19:14650602:14651688,DRE_3:40674383:40676,OLA_11:9858403:98590,TNI_21randor
OMM1643
OMM5153 DRE_16:23924872:23934646,OLA_11:10285718:10287743,TNI_21:3149328:3150910,TRU_scaffold12:2212846:2214343
OMM5143 DRE_19:37234948:37248578,OLA_11:13090366:13092405,TNI_21:3958092:3960816,TRU_scaffold12:1271875:1273694
OMM1038
OMM3044

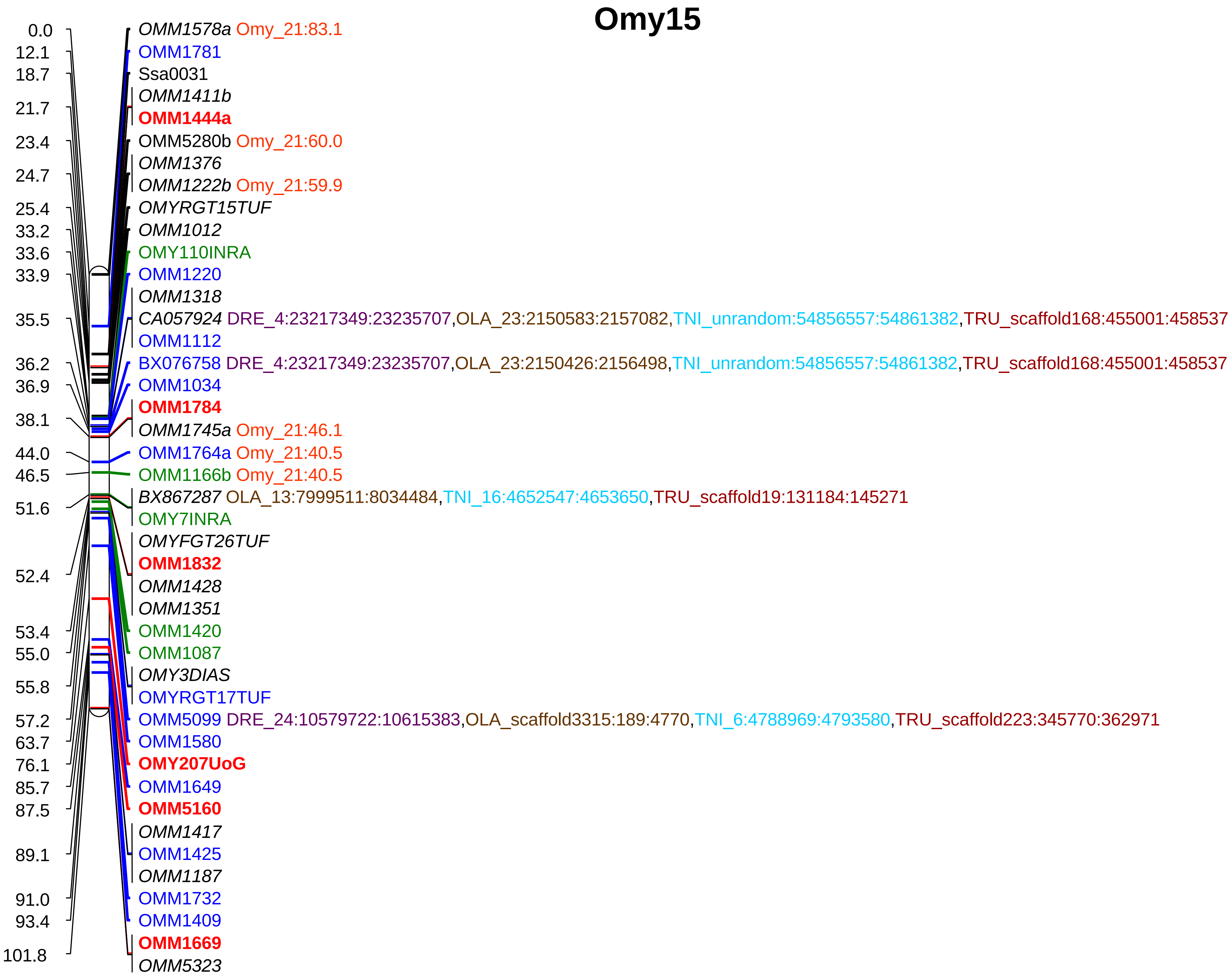
Omy15
0.0
12.1
18.7
21.7
23.4
24.7
25.4
33.2
33.6
33.9
35.5
36.2
36.9
38.1
44.0
46.5
51.6
52.4
53.4
55.0
55.8
57.2
63.7
76.1
85.7
87.5
89.1
91.0
93.4
101.8
OMM1578a Omy_21:83.1
OMM1781
Ssa0031
OMM1411b
OMM1444a
OMM5280b Omy_21:60.0
OMM1376
OMM1222b Omy_21:59.9
OMYRGT15TUF
OMM1012
OMY110INRA
OMM1220
OMM1318
CA057924 DRE_4:23217349:23235707,OLA_23:2150583:2157082,TNI_unrandom:54856557:54861382,TRU_scaffold168:455001:458537
OMM1112
BX076758 DRE_4:23217349:23235707,OLA_23:2150426:2156498,TNI_unrandom:54856557:54861382,TRU_scaffold168:455001:458537
OMM1034
OMM1784
OMM1745a Omy_21:46.1
OMM1764a Omy_21:40.5
OMM1166b Omy_21:40.5
BX867287 OLA_13:7999511:8034484,TNI_16:4652547:4653650,TRU_scaffold19:131184:145271
OMY7INRA
OMYFGT26TUF
OMM1832
OMM1428
OMM1351
OMM1420
OMM1087
OMY3DIAS
OMYRGT17TUF
OMM5099 DRE_24:10579722:10615383,OLA_scaffold3315:189:4770,TNI_6:4788969:4793580,TRU_scaffold223:345770:362971
OMM1580
OMY207UoG
OMM1649
OMM5160
OMM1417
OMM1425
OMM1187
OMM1732
OMM1409
OMM1669
OMM5323

Omy16

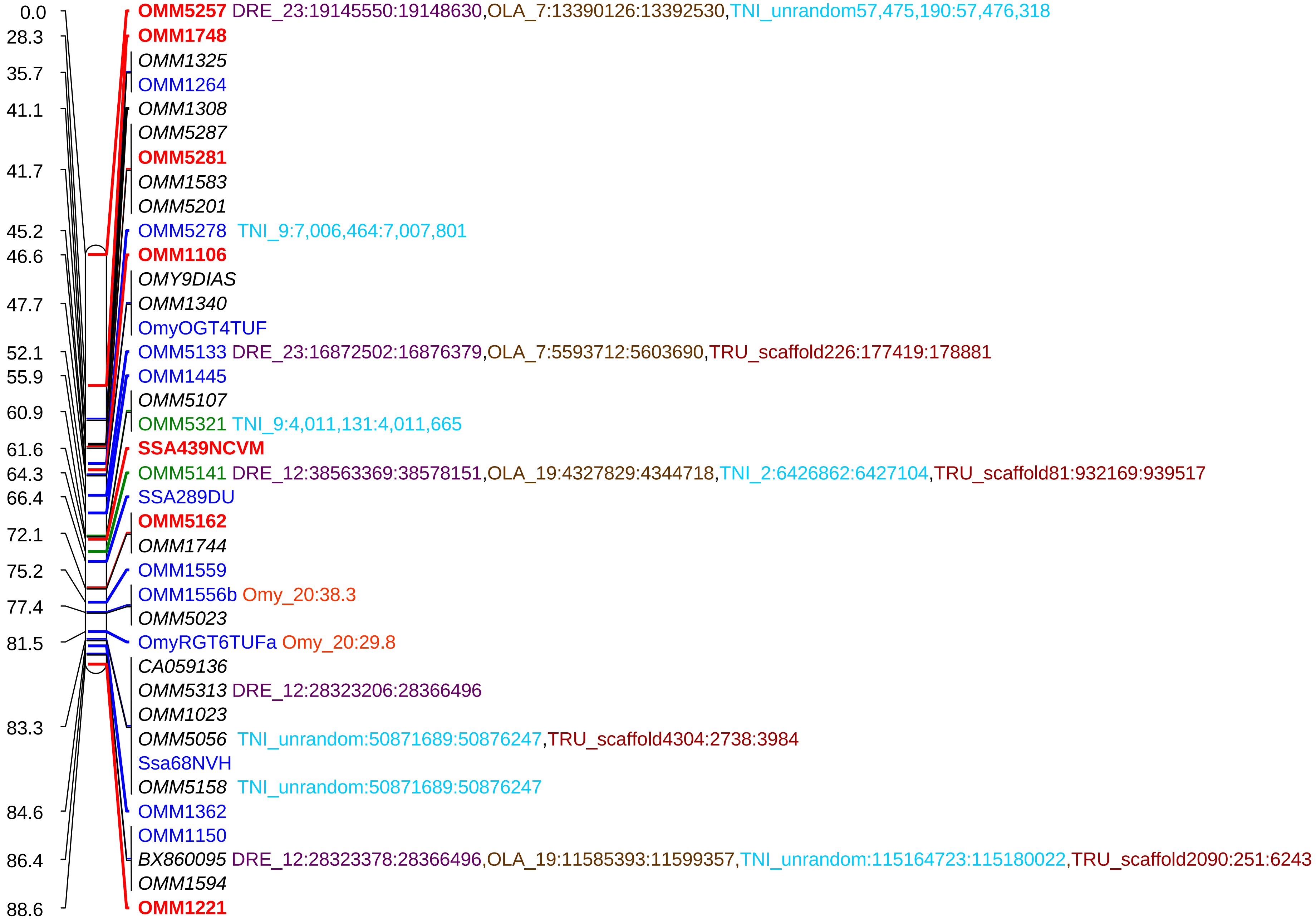
0.0
28.3
35.7
41.1
41.7
45.2
46.6
47.7
52.1
55.9
60.9
61.6
64.3
66.4
72.1
75.2
77.4
81.5
83.3
84.6
86.4
88.6
OMM5257 DRE_23:19145550:19148630,OLA_7:13390126:13392530,TNI_unrandom57,475,190:57,476,318
OMM1748
OMM1325
OMM1264
OMM1308
OMM5287
OMM5281
OMM1583
OMM5201
OMM5278 TNI_9:7,006,464:7,007,801
OMM1106
OMY9DIAS
OMM1340
OmyOGT4TUF
OMM5133 DRE_23:16872502:16876379,OLA_7:5593712:5603690,TRU_scaffold226:177419:178881
OMM1445
OMM5107
OMM5321 TNI_9:4,011,131:4,011,665
SSA439NCVM
OMM5141 DRE_12:38563369:38578151,OLA_19:4327829:4344718,TNI_2:6426862:6427104,TRU_scaffold81:932169:939517
SSA289DU
OMM5162
OMM1744
OMM1559
OMM1556b Omy_20:38.3
OMM5023
OmyRGT6TUFa Omy_20:29.8
CA059136
OMM5313 DRE_12:28323206:28366496
OMM1023
OMM5056 TNI_unrandom:50871689:50876247,TRU_scaffold4304:2738:3984
Ssa68NVH
OMM5158 TNI_unrandom:50871689:50876247
OMM1362
OMM1150
BX860095 DRE_12:28323378:28366496,OLA_19:11585393:11599357,TNI_unrandom:115164723:115180022,TRU_scaffold2090:251:6243
OMM1594
OMM1221

Omy17

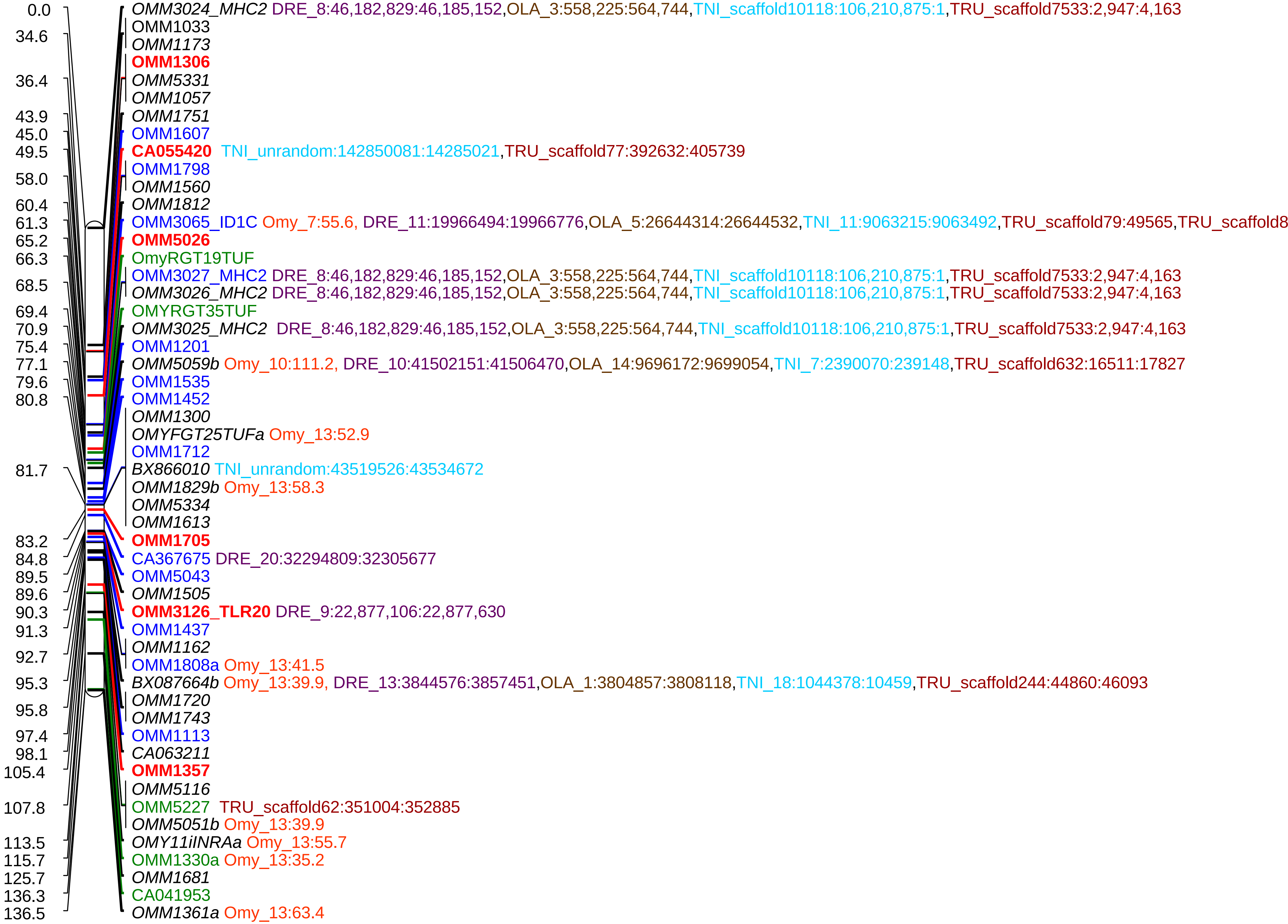
0.0
34.6
36.4
43.9
45.0
49.5
58.0
60.4
61.3
65.2
66.3
68.5
69.4
70.9
75.4
77.1
79.6
80.8
81.7
83.2
84.8
89.5
89.6
90.3
91.3
92.7
95.3
95.8
97.4
98.1
105.4
107.8
113.5
115.7
125.7
136.3
136.5
OMM3024_MHC2 DRE_8:46,182,829:46,185,152,OLA_3:558,225:564,744,TNI_scaffold10118:106,210,875:1,TRU_scaffold7533:2,947:4,163
OMM1033
OMM1173
OMM1306
OMM5331
OMM1057
OMM1751
OMM1607
CA055420 TNI_unrandom:142850081:14285021,TRU_scaffold77:392632:405739
OMM1798
OMM1560
OMM1812
OMM3065_ID1C Omy_7:55.6, DRE_11:19966494:19966776,OLA_5:26644314:26644532,TNI_11:9063215:9063492,TRU_scaffold79:49565,TRU_scaffold8
OMM5026
OmyRGT19TUF
OMM3027_MHC2 DRE_8:46,182,829:46,185,152,OLA_3:558,225:564,744,TNI_scaffold10118:106,210,875:1,TRU_scaffold7533:2,947:4,163
OMM3026_MHC2 DRE_8:46,182,829:46,185,152,OLA_3:558,225:564,744,TNI_scaffold10118:106,210,875:1,TRU_scaffold7533:2,947:4,163
OMYRGT35TUF
OMM3025_MHC2 DRE_8:46,182,829:46,185,152,OLA_3:558,225:564,744,TNI_scaffold10118:106,210,875:1,TRU_scaffold7533:2,947:4,163
OMM1201
OMM5059b Omy_10:111.2, DRE_10:41502151:41506470,OLA_14:9696172:9699054,TNI_7:2390070:239148,TRU_scaffold632:16511:17827
OMM1535
OMM1452
OMM1300
OMYFGT25TUFa Omy_13:52.9
OMM1712
BX866010 TNI_unrandom:43519526:43534672
OMM1829b Omy_13:58.3
OMM5334
OMM1613
OMM1705
CA367675 DRE_20:32294809:32305677
OMM5043
OMM1505
OMM3126_TLR20 DRE_9:22,877,106:22,877,630
OMM1437
OMM1162
OMM1808a Omy_13:41.5
BX087664b Omy_13:39.9, DRE_13:3844576:3857451,OLA_1:3804857:3808118,TNI_18:1044378:10459,TRU_scaffold244:44860:46093
OMM1720
OMM1743
OMM1113
CA063211
OMM1357
OMM5116
OMM5227 TRU_scaffold62:351004:352885
OMM5051b Omy_13:39.9
OMY11iINRAa Omy_13:55.7
OMM1330a Omy_13:35.2
OMM1681
CA041953
OMM1361a Omy_13:63.4

Omy18

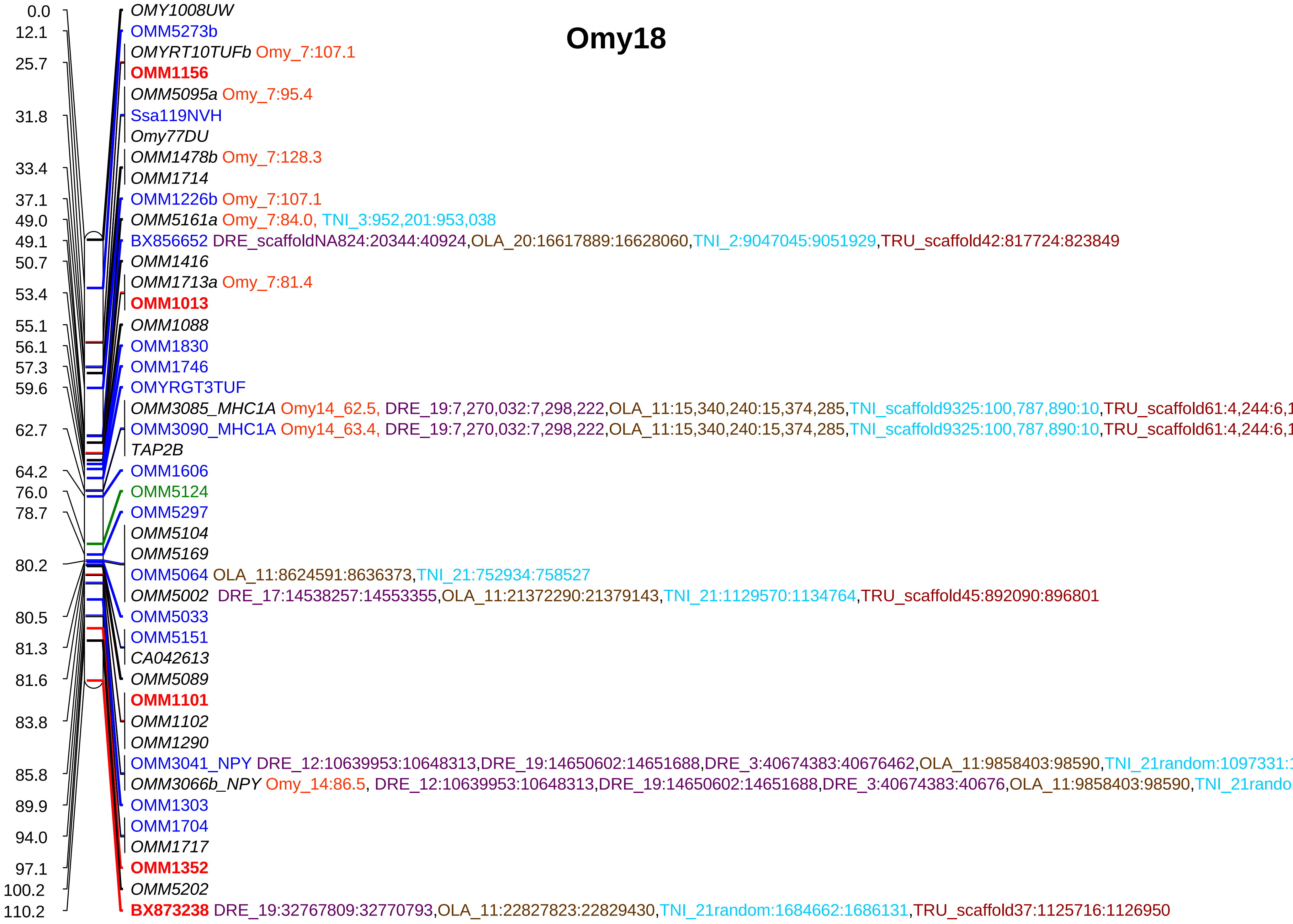
0.0 OMY1008UW
12.1 OMM5273b
OMYRT10TUFb Omy_7:107.1
25.7 OMM1156
OMM5095a Omy_7:95.4
31.8 Ssa119NVH
Omy77DU
OMM1478b Omy_7:128.3
33.4 OMM1714
37.1 OMM1226b Omy_7:107.1
49.0 OMM5161a Omy_7:84.0, TNI_3:952,201:953,038
49.1 BX856652 DRE_scaffoldNA824:20344:40924,OLA_20:16617889:16628060,TNI_2:9047045:9051929,TRU_scaffold42:817724:823849
50.7 OMM1416
OMM1713a Omy_7:81.4
53.4 OMM1013
55.1 OMM1088
56.1 OMM1830
57.3 OMM1746
59.6 OMYRGT3TUF
OMM3085_MHC1A Omy14_62.5, DRE_19:7,270,032:7,298,222,OLA_11:15,340,240:15,374,285,TNI_scaffold9325:100,787,890:10,TRU_scaffold61:4,244:6,1
62.7 OMM3090_MHC1A Omy14_63.4, DRE_19:7,270,032:7,298,222,OLA_11:15,340,240:15,374,285,TNI_scaffold9325:100,787,890:10,TRU_scaffold61:4,244:6,1
TAP2B
64.2 OMM1606
76.0 OMM5124
78.7 OMM5297
OMM5104
OMM5169
80.2 OMM5064 OLA_11:8624591:8636373,TNI_21:752934:758527
OMM5002 DRE_17:14538257:14553355,OLA_11:21372290:21379143,TNI_21:1129570:1134764,TRU_scaffold45:892090:896801
80.5 OMM5033
OMM5151
81.3 CA042613
81.6 OMM5089
OMM1101
83.8 OMM1102
OMM1290
OMM3041_NPY DRE_12:10639953:10648313,DRE_19:14650602:14651688,DRE_3:40674383:40676462,OLA_11:9858403:98590,TNI_21random:1097331:1
85.8 OMM3066b_NPY Omy_14:86.5, DRE_12:10639953:10648313,DRE_19:14650602:14651688,DRE_3:40674383:40676,OLA_11:9858403:98590,TNI_21rando
89.9 OMM1303
OMM1704
94.0 OMM1717
97.1 OMM1352
100.2 OMM5202
110.2 BX873238 DRE_19:32767809:32770793,OLA_11:22827823:22829430,TNI_21random:1684662:1686131,TRU_scaffold37:1125716:1126950

Omy19

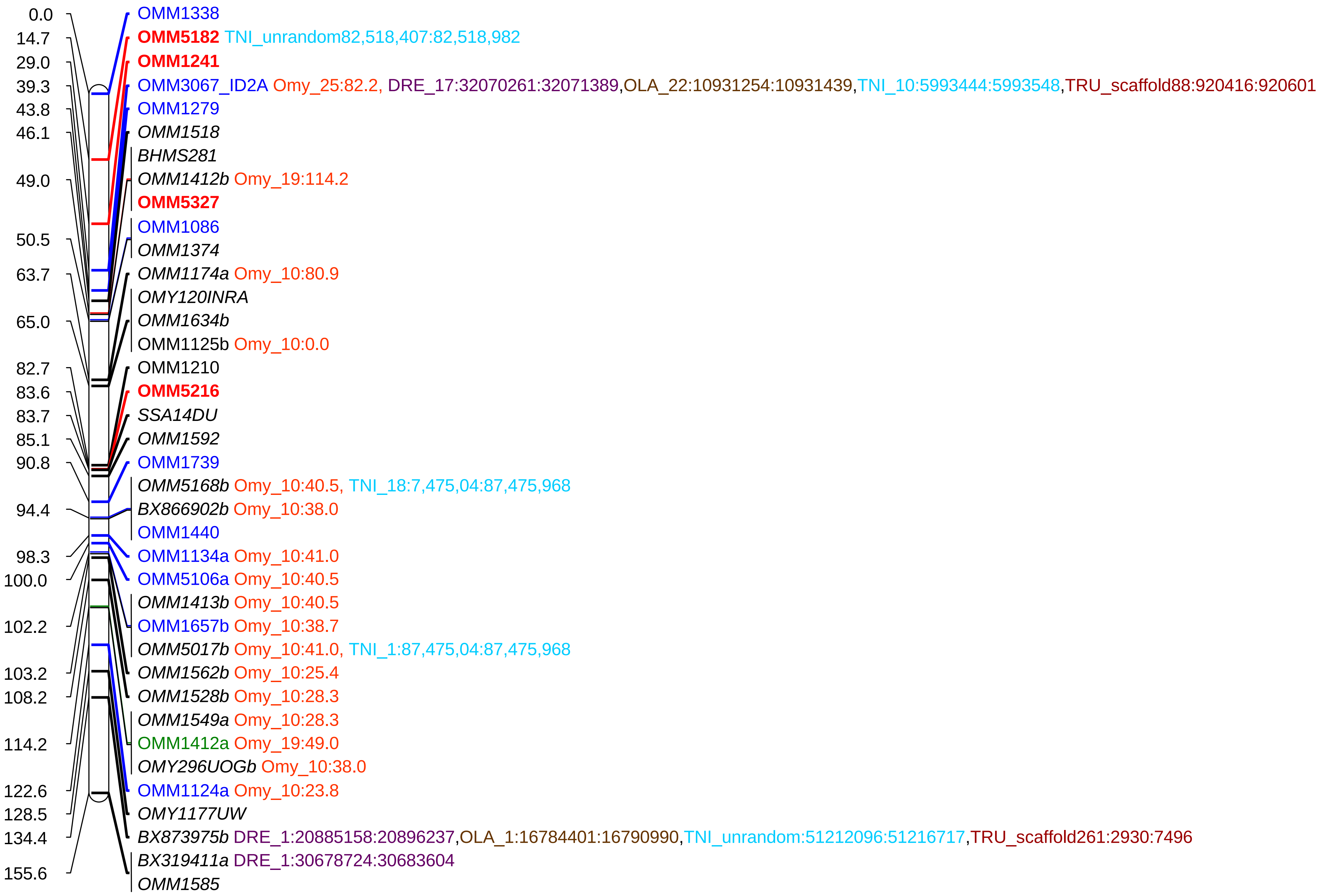
0.0 OMM1338
14.7 OMM5182 TNI_unrandom82,518,407:82,518,982
29.0 OMM1241
39.3 OMM3067_ID2A Omy_25:82.2, DRE_17:32070261:32071389,OLA_22:10931254:10931439,TNI_10:5993444:5993548,TRU_scaffold88:920416:920601
43.8 OMM1279
46.1 OMM1518
BHMS281
49.0 OMM1412b Omy_19:114.2
OMM5327
OMM1086
50.5 OMM1374
63.7 OMM1174a Omy_10:80.9
OMY120INRA
65.0 OMM1634b
OMM1125b Omy_10:0.0
82.7 OMM1210
83.6 OMM5216
83.7 SSA14DU
85.1 OMM1592
90.8 OMM1739
OMM5168b Omy_10:40.5, TNI_18:7,475,04:87,475,968
94.4 BX866902b Omy_10:38.0
OMM1440
98.3 OMM1134a Omy_10:41.0
100.0 OMM5106a Omy_10:40.5
OMM1413b Omy_10:40.5
102.2 OMM1657b Omy_10:38.7
OMM5017b Omy_10:41.0, TNI_1:87,475,04:87,475,968
103.2 OMM1562b Omy_10:25.4
108.2 OMM1528b Omy_10:28.3
OMM1549a Omy_10:28.3
114.2 OMM1412a Omy_19:49.0
OMY296UOGb Omy_10:38.0
122.6 OMM1124a Omy_10:23.8
128.5 OMY1177UW
134.4 BX873975b DRE_1:20885158:20896237,OLA_1:16784401:16790990,TNI_unrandom:51212096:51216717,TRU_scaffold261:2930:7496
BX319411a DRE_1:30678724:30683604
155.6 OMM1585

Omy20

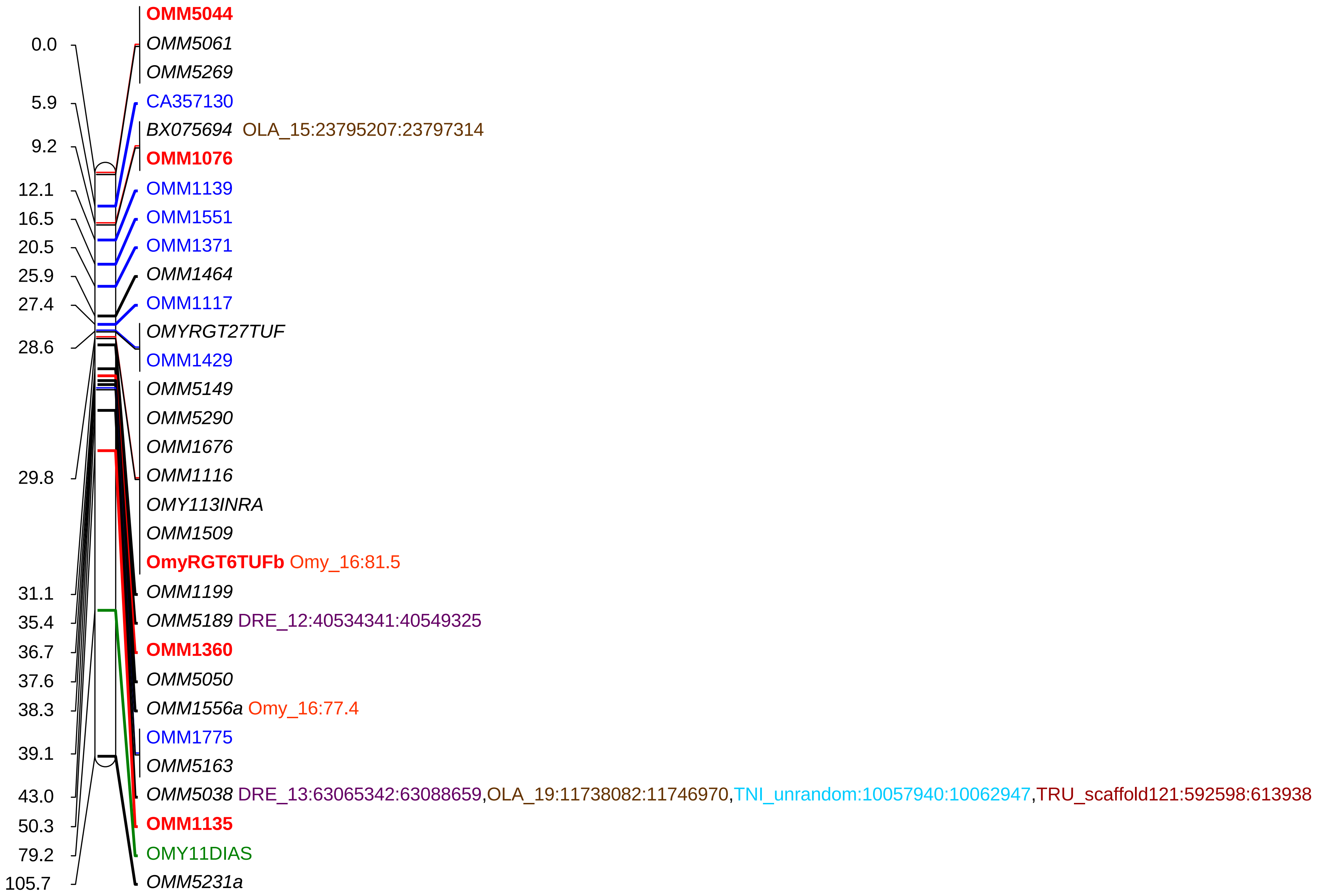

0.0
5.9
9.2
12.1
16.5
20.5
25.9
27.4
28.6
29.8
31.1
35.4
36.7
37.6
38.3
39.1
43.0
50.3
79.2
105.7
OMM5044
OMM5061
OMM5269
CA357130
BX075694 OLA_15:23795207:23797314
OMM1076
OMM1139
OMM1551
OMM1371
OMM1464
OMM1117
OMYRGT27TUF
OMM1429
OMM5149
OMM5290
OMM1676
OMM1116
OMY113INRA
OMM1509
OmyRGT6TUFb Omy_16:81.5
OMM1199
OMM5189 DRE_12:40534341:40549325
OMM1360
OMM5050
OMM1556a Omy_16:77.4
OMM1775
OMM5163
OMM5038 DRE_13:63065342:63088659,OLA_19:11738082:11746970,TNI_unrandom:10057940:10062947,TRU_scaffold121:592598:613938
OMM1135
OMY11DIAS
OMM5231a

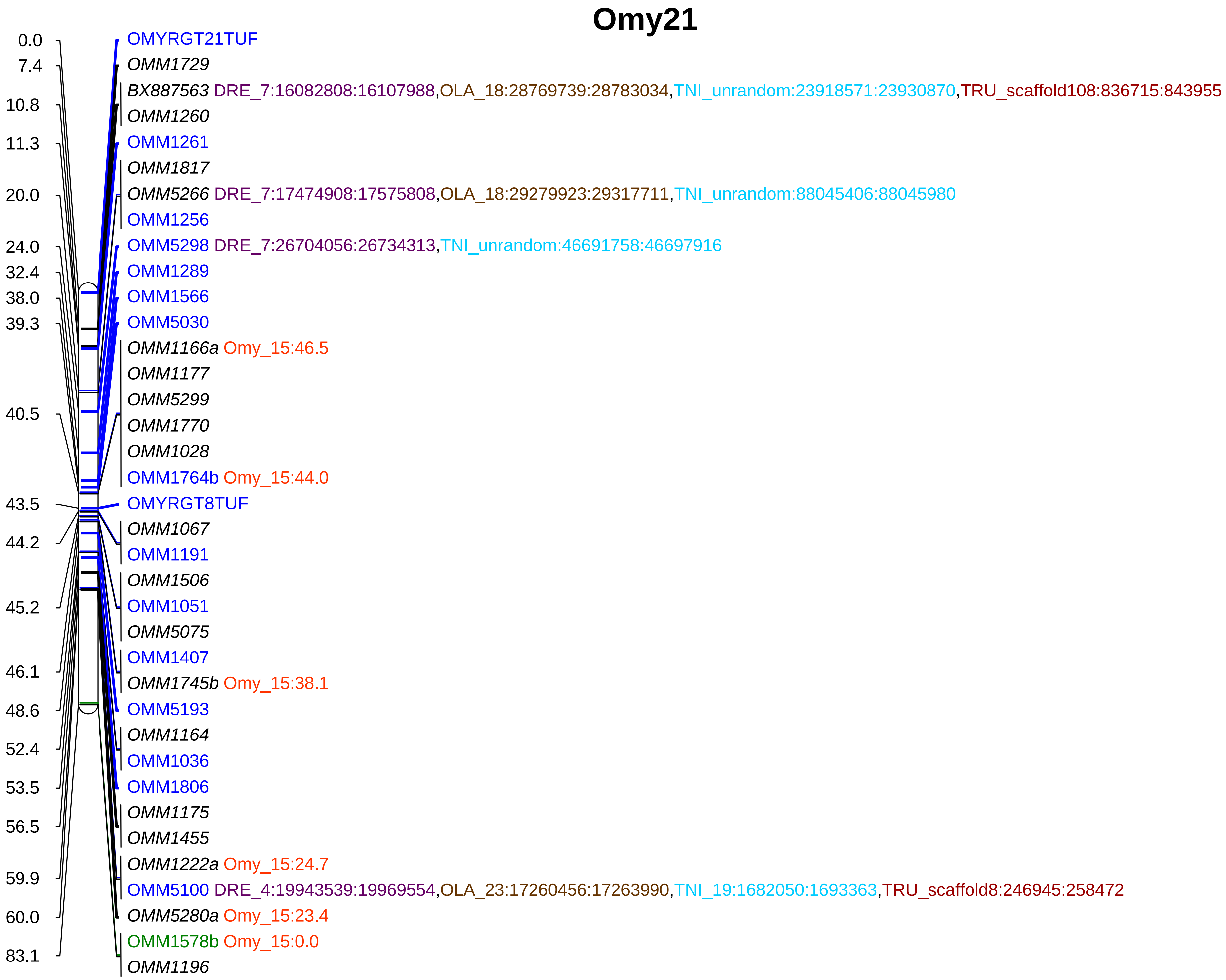
Omy21
0.0
7.4
10.8
11.3
20.0
24.0
32.4
38.0
39.3
40.5
43.5
44.2
45.2
46.1
48.6
52.4
53.5
56.5
59.9
60.0
83.1
OMYRGT21TUF
OMM1729
BX887563 DRE_7:16082808:16107988,OLA_18:28769739:28783034,TNI_unrandom:23918571:23930870,TRU_scaffold108:836715:843955
OMM1260
OMM1261
OMM1817
OMM5266 DRE_7:17474908:17575808,OLA_18:29279923:29317711,TNI_unrandom:88045406:88045980
OMM1256
OMM5298 DRE_7:26704056:26734313,TNI_unrandom:46691758:46697916
OMM1289
OMM1566
OMM5030
OMM1166a Omy_15:46.5
OMM1177
OMM5299
OMM1770
OMM1028
OMM1764b Omy_15:44.0
OMYRGT8TUF
OMM1067
OMM1191
OMM1506
OMM1051
OMM5075
OMM1407
OMM1745b Omy_15:38.1
OMM5193
OMM1164
OMM1036
OMM1806
OMM1175
OMM1455
OMM1222a Omy_15:24.7
OMM5100 DRE_4:19943539:19969554,OLA_23:17260456:17263990,TNI_19:1682050:1693363,TRU_scaffold8:246945:258472
OMM5280a Omy_15:23.4
OMM1578b Omy_15:0.0
OMM1196

Omy22

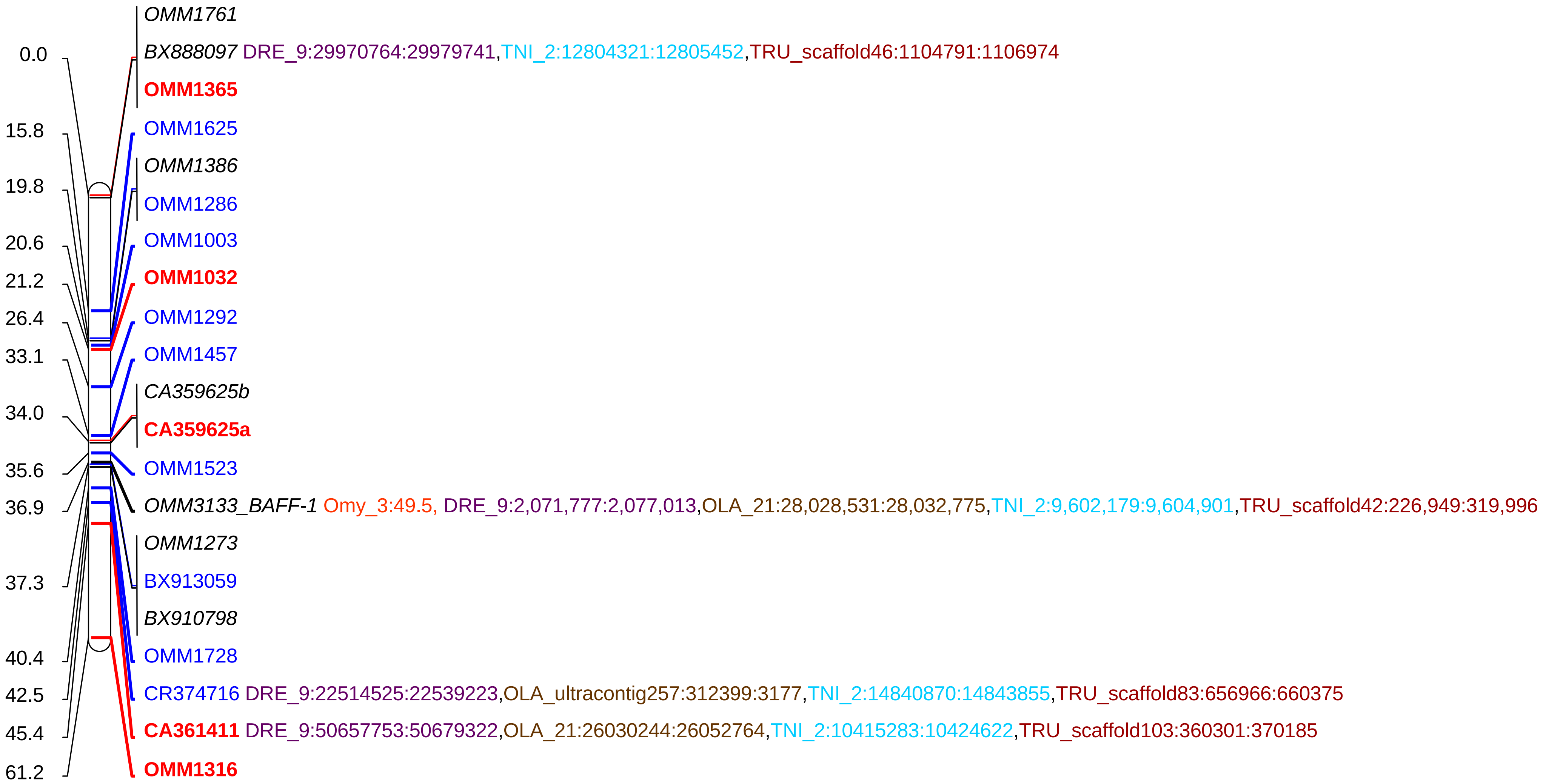
0.0
15.8
19.8
20.6
21.2
26.4
33.1
34.0
35.6
36.9
37.3
40.4
42.5
45.4
61.2
OMM1761
BX888097 DRE_9:29970764:29979741,TNI_2:12804321:12805452,TRU_scaffold46:1104791:1106974
OMM1365
OMM1625
OMM1386
OMM1286
OMM1003
OMM1032
OMM1292
OMM1457
CA359625b
CA359625a
OMM1523
OMM3133_BAFF-1 Omy_3:49.5, DRE_9:2,071,777:2,077,013,OLA_21:28,028,531:28,032,775,TNI_2:9,602,179:9,604,901,TRU_scaffold42:226,949:319,996
OMM1273
BX913059
BX910798
OMM1728
CR374716 DRE_9:22514525:22539223,OLA_ultracontig257:312399:3177,TNI_2:14840870:14843855,TRU_scaffold83:656966:660375
CA361411 DRE_9:50657753:50679322,OLA_21:26030244:26052764,TNI_2:10415283:10424622,TRU_scaffold103:360301:370185
OMM1316

Omy23

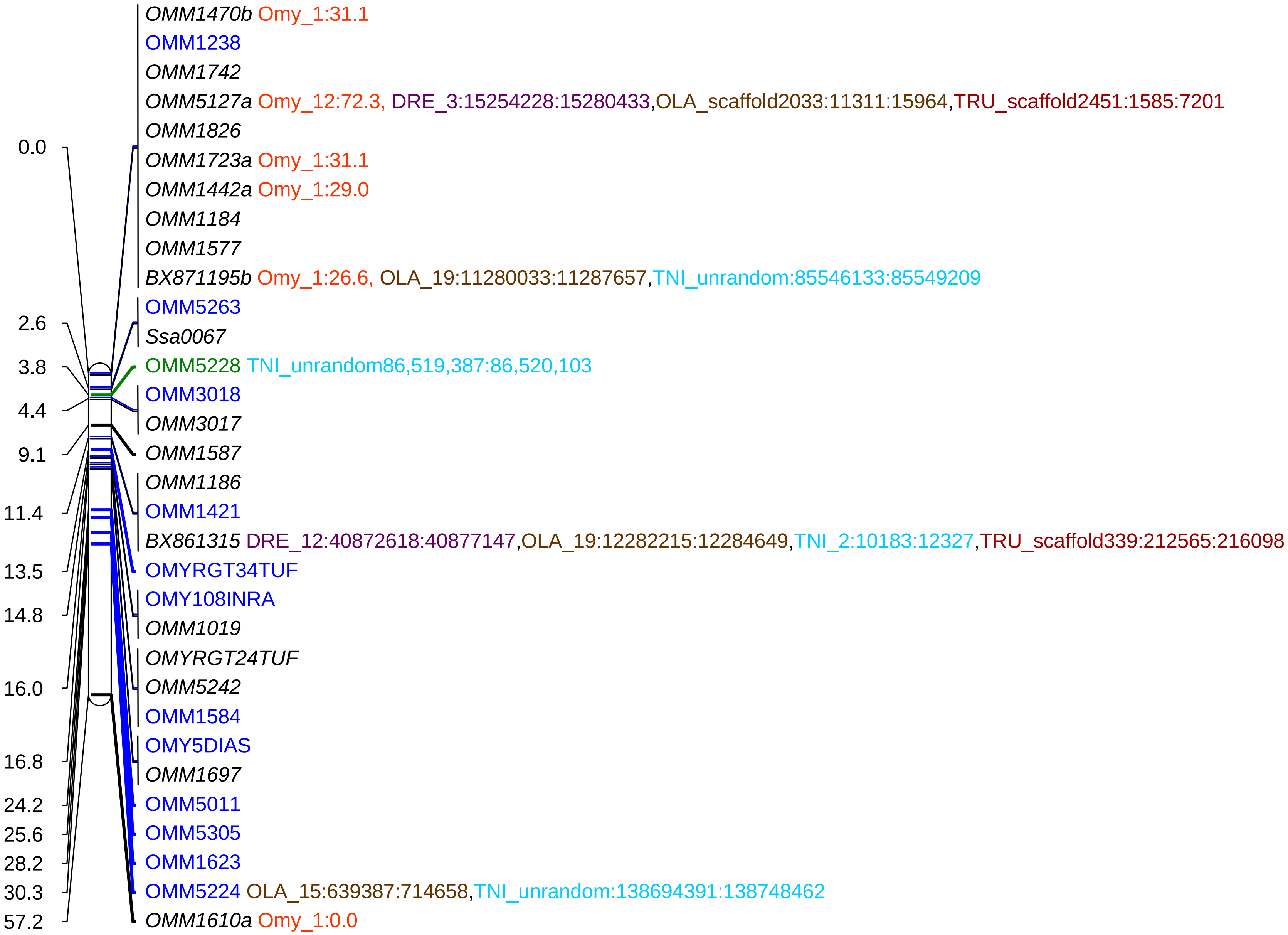
0.0
2.6
3.8
4.4
9.1
11.4
13.5
14.8
16.0
16.8
24.2
25.6
28.2
30.3
57.2
OMM1470b Omy_1:31.1
OMM1238
OMM1742
OMM5127a Omy_12:72.3, DRE_3:15254228:15280433,OLA_scaffold2033:11311:15964,TRU_scaffold2451:1585:7201
OMM1826
OMM1723a Omy_1:31.1
OMM1442a Omy_1:29.0
OMM1184
OMM1577
BX871195b Omy_1:26.6, OLA_19:11280033:11287657,TNI_unrandom:85546133:85549209
OMM5263
Ssa0067
OMM5228 TNI_unrandom86,519,387:86,520,103
OMM3018
OMM3017
OMM1587
OMM1186
OMM1421
BX861315 DRE_12:40872618:40877147,OLA_19:12282215:12284649,TNI_2:10183:12327,TRU_scaffold339:212565:216098
OMYRGT34TUF
OMY108INRA
OMM1019
OMYRGT24TUF
OMM5242
OMM1584
OMY5DIAS
OMM1697
OMM5011
OMM5305
OMM1623
OMM5224 OLA_15:639387:714658,TNI_unrandom:138694391:138748462
OMM1610a Omy_1:0.0

Omy24

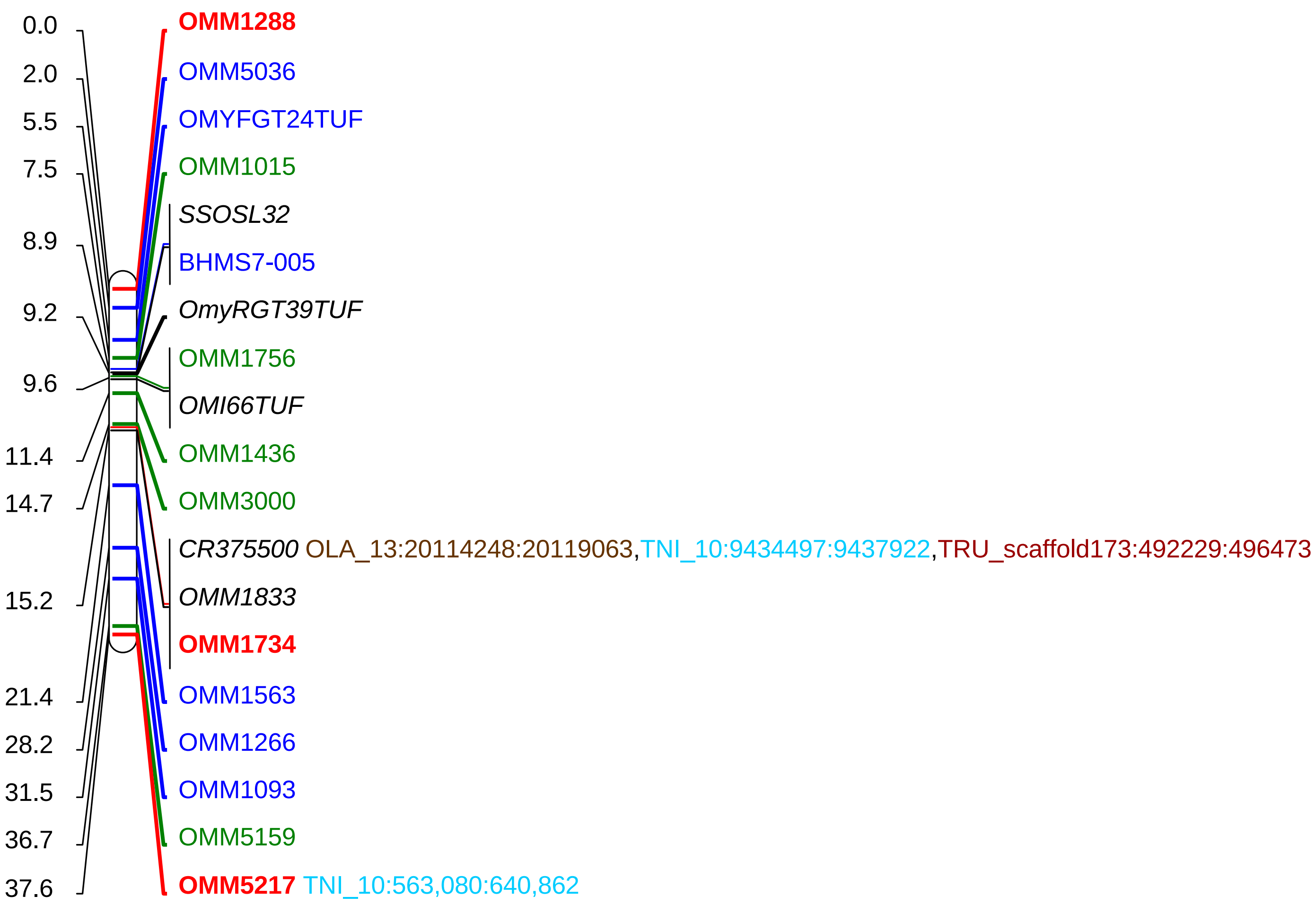
0.0
2.0
5.5
7.5
8.9
9.2
9.6
11.4
14.7
15.2
21.4
28.2
31.5
36.7
37.6
OMM1288
OMM5036
OMYFGT24TUF
OMM1015
SSOSL32
BHMS7-005
OmyRGT39TUF
OMM1756
OMI66TUF
OMM1436
OMM3000
CR375500 OLA_13:20114248:20119063,TNI_10:9434497:9437922,TRU_scaffold173:492229:496473
OMM1833
OMM1734
OMM1563
OMM1266
OMM1093
OMM5159
OMM5217 TNI_10:563,080:640,862

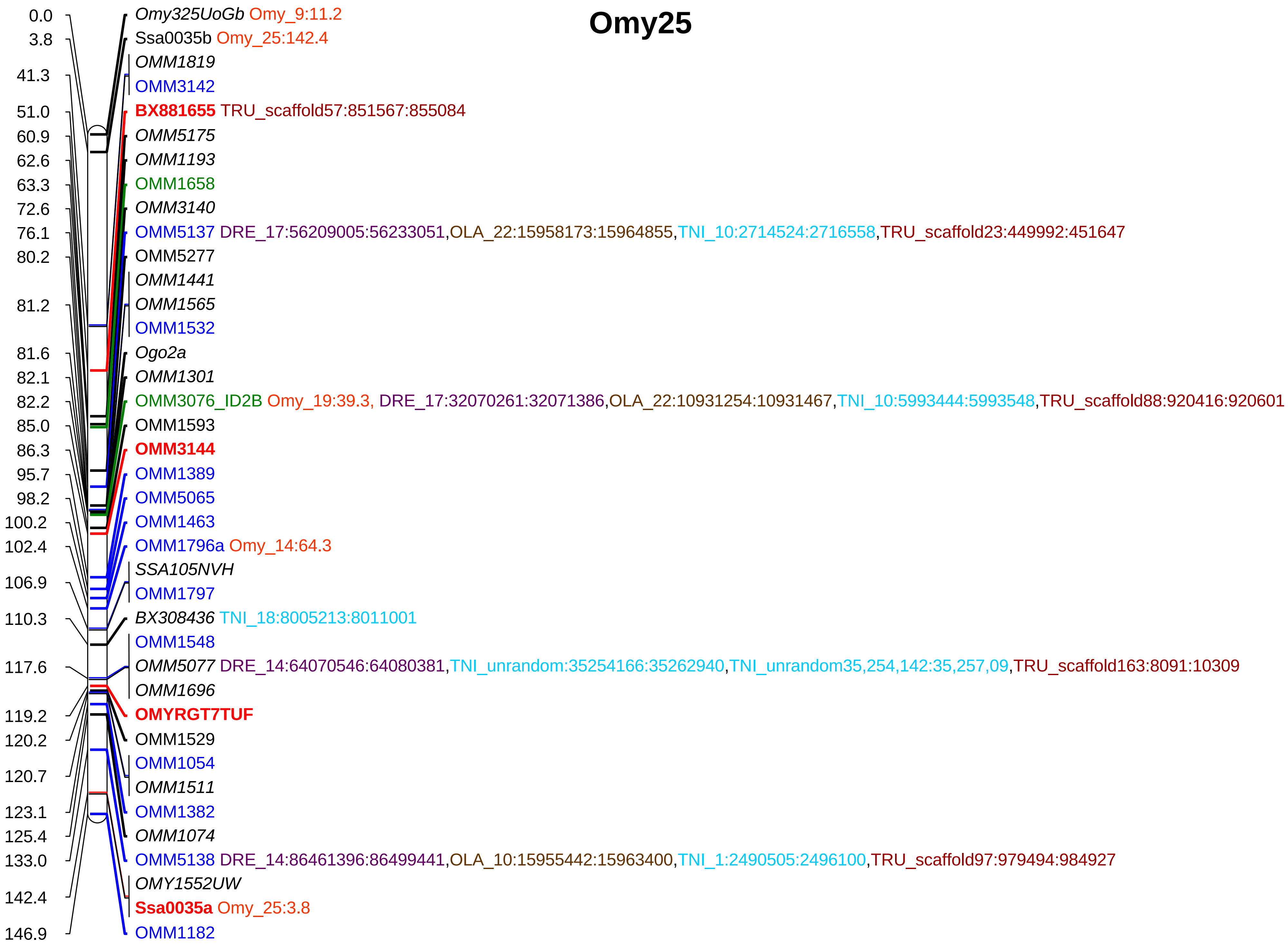
Omy25
0.0
3.8
41.3
51.0
60.9
62.6
63.3
72.6
76.1
80.2
81.2
81.6
82.1
82.2
85.0
86.3
95.7
98.2
100.2
102.4
106.9
110.3
117.6
119.2
120.2
120.7
123.1
125.4
133.0
142.4
146.9
Omy325UoGb Omy_9:11.2
Ssa0035b Omy_25:142.4
OMM1819
OMM3142
BX881655 TRU_scaffold57:851567:855084
OMM5175
OMM1193
OMM1658
OMM3140
OMM5137 DRE_17:56209005:56233051,OLA_22:15958173:15964855,TNI_10:2714524:2716558,TRU_scaffold23:449992:451647
OMM5277
OMM1441
OMM1565
OMM1532
Ogo2a
OMM1301
OMM3076_ID2B Omy_19:39.3, DRE_17:32070261:32071386,OLA_22:10931254:10931467,TNI_10:5993444:5993548,TRU_scaffold88:920416:920601
OMM1593
OMM3144
OMM1389
OMM5065
OMM1463
OMM1796a Omy_14:64.3
SSA105NVH
OMM1797
BX308436 TNI_18:8005213:8011001
OMM1548
OMM5077 DRE_14:64070546:64080381,TNI_unrandom:35254166:35262940,TNI_unrandom35,254,142:35,257,09,TRU_scaffold163:8091:10309
OMM1696
OMYRGT7TUF
OMM1529
OMM1054
OMM1511
OMM1382
OMM1074
OMM5138 DRE_14:86461396:86499441,OLA_10:15955442:15963400,TNI_1:2490505:2496100,TRU_scaffold97:979494:984927
OMY1552UW
Ssa0035a Omy_25:3.8
OMM1182

Omy26

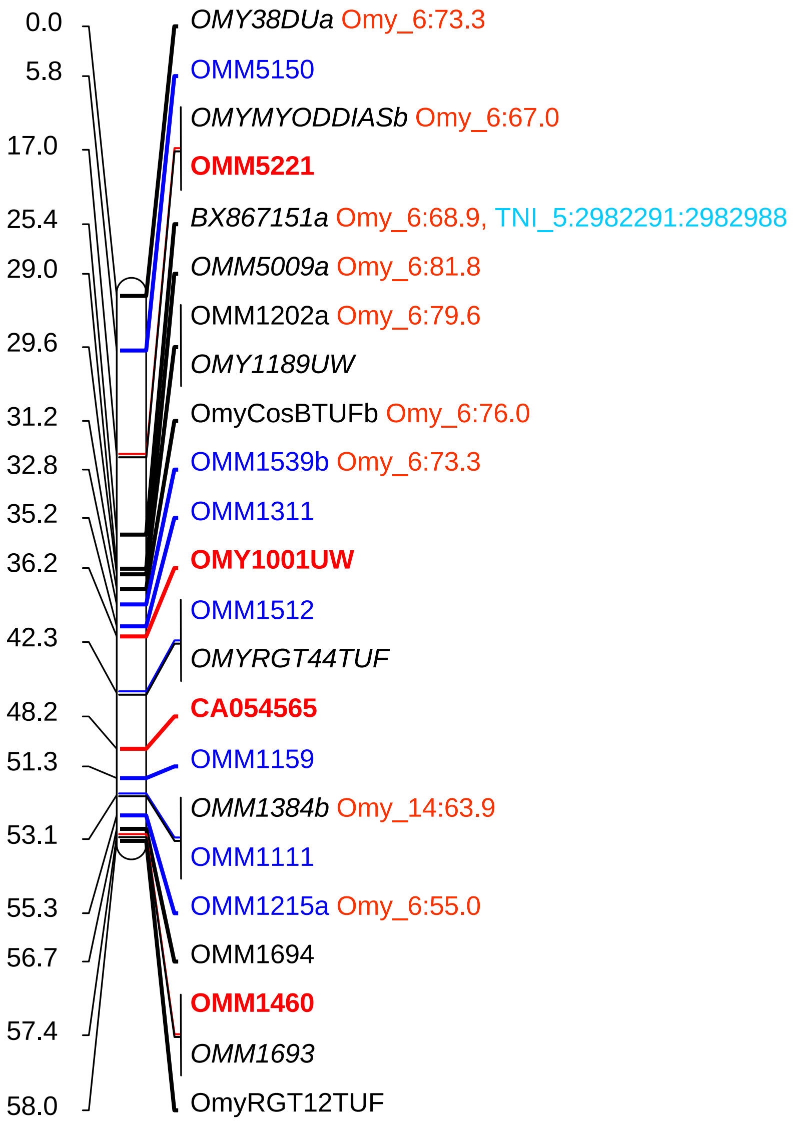
0.0
5.8
17.0
25.4
29.0
29.6
31.2
32.8
35.2
36.2
42.3
48.2
51.3
53.1
55.3
56.7
57.4
58.0
OMY38DUa Omy_6:73.3
OMM5150
OMYMYODDIASb Omy_6:67.0
OMM5221
BX867151a Omy_6:68.9, TNI_5:2982291:2982988
OMM5009a Omy_6:81.8
OMM1202a Omy_6:79.6
OMY1189UW
OmyCosBTUFb Omy_6:76.0
OMM1539b Omy_6:73.3
OMM1311
OMY1001UW
OMM1512
OMYRGT44TUF
CA054565
OMM1159
OMM1384b Omy_14:63.9
OMM1111
OMM1215a Omy_6:55.0
OMM1694
OMM1460
OMM1693
OmyRGT12TUF

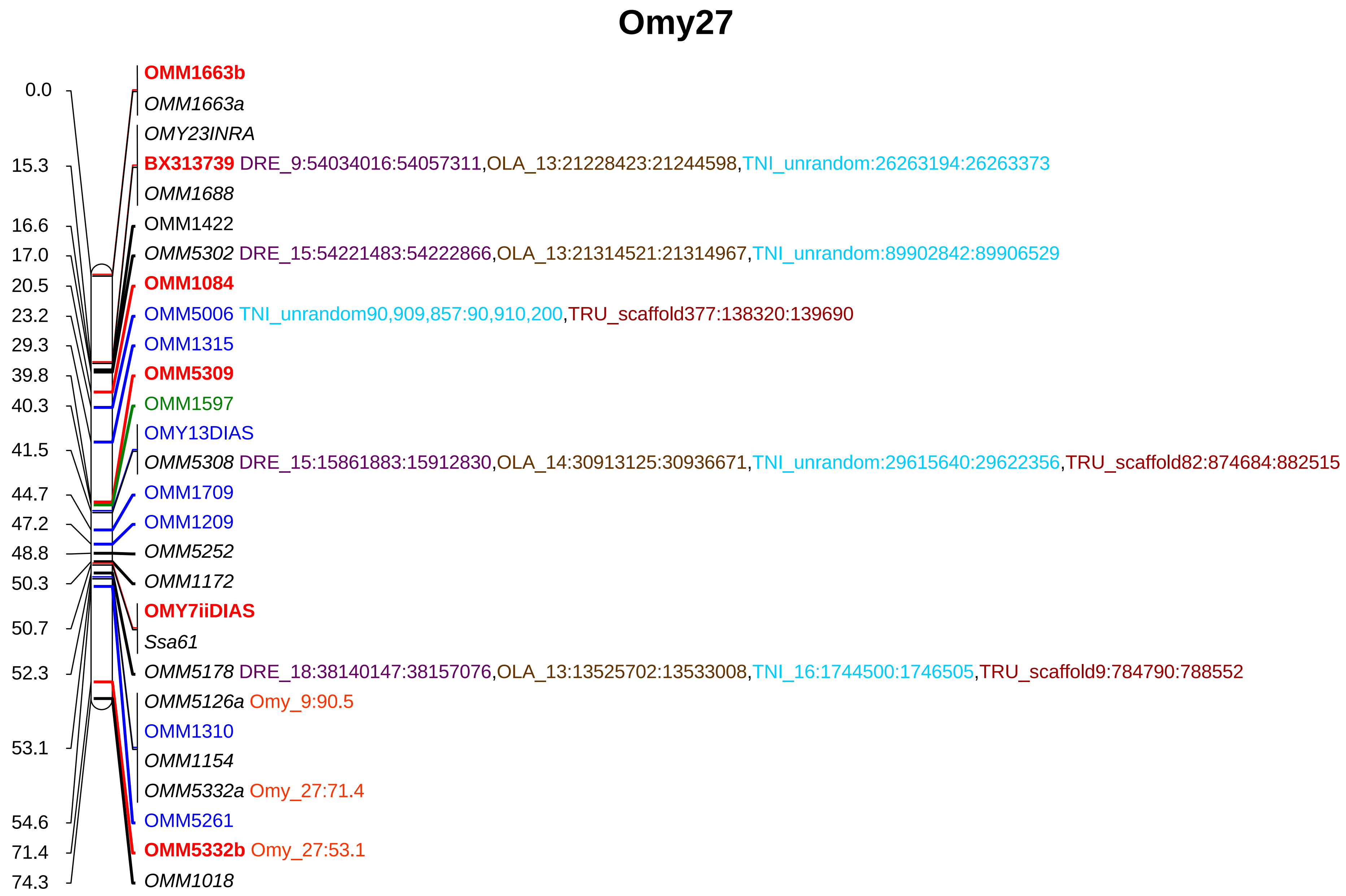
Omy27
0.0
15.3
16.6
17.0
20.5
23.2
29.3
39.8
40.3
41.5
44.7
47.2
48.8
50.3
50.7
52.3
53.1
54.6
71.4
74.3
OMM1663b
OMM1663a
OMY23INRA
BX313739 DRE_9:54034016:54057311,OLA_13:21228423:21244598,TNI_unrandom:26263194:26263373
OMM1688
OMM1422
OMM5302 DRE_15:54221483:54222866,OLA_13:21314521:21314967,TNI_unrandom:89902842:89906529
OMM1084
OMM5006 TNI_unrandom90,909,857:90,910,200,TRU_scaffold377:138320:139690
OMM1315
OMM5309
OMM1597
OMY13DIAS
OMM5308 DRE_15:15861883:15912830,OLA_14:30913125:30936671,TNI_unrandom:29615640:29622356,TRU_scaffold82:874684:882515
OMM1709
OMM1209
OMM5252
OMM1172
OMY7iiDIAS
Ssa61
OMM5178 DRE_18:38140147:38157076,OLA_13:13525702:13533008,TNI_16:1744500:1746505,TRU_scaffold9:784790:788552
OMM5126a Omy_9:90.5
OMM1310
OMM1154
OMM5332a Omy_27:71.4
OMM5261
OMM5332b Omy_27:53.1
OMM1018

Omy28

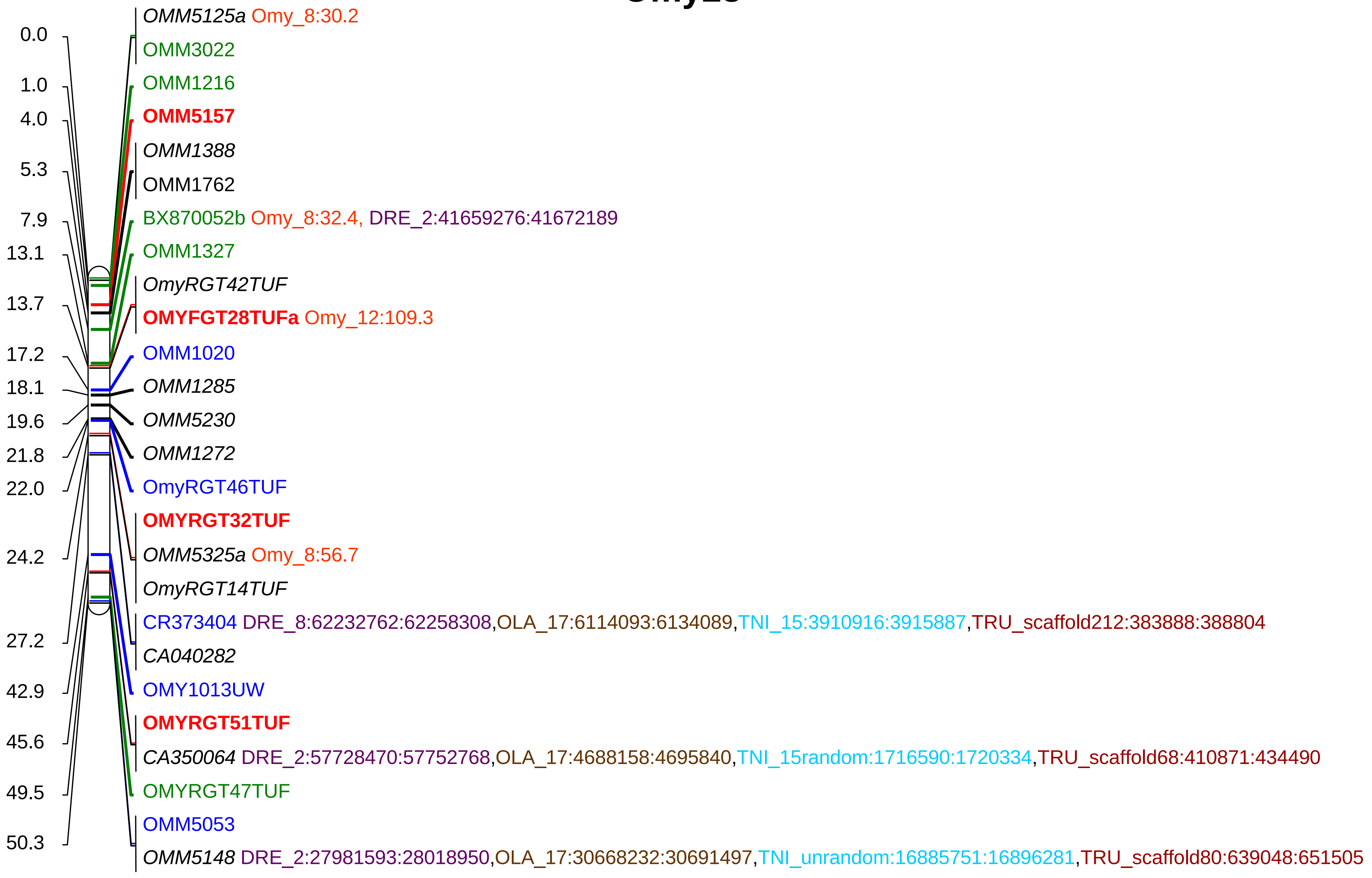
0.0
1.0
4.0
5.3
7.9
13.1
13.7
17.2
18.1
19.6
21.8
22.0
24.2
27.2
42.9
45.6
49.5
50.3
OMM5125a Omy_8:30.2
OMM3022
OMM1216
OMM5157
OMM1388
OMM1762
BX870052b Omy_8:32.4, DRE_2:41659276:41672189
OMM1327
OmyRGT42TUF
OMYFGT28TUFa Omy_12:109.3
OMM1020
OMM1285
OMM5230
OMM1272
OmyRGT46TUF
OMYRGT32TUF
OMM5325a Omy_8:56.7
OmyRGT14TUF
CR373404 DRE_8:62232762:62258308,OLA_17:6114093:6134089,TNI_15:3910916:3915887,TRU_scaffold212:383888:388804
CA040282
OMY1013UW
OMYRGT51TUF
CA350064 DRE_2:57728470:57752768,OLA_17:4688158:4695840,TNI_15random:1716590:1720334,TRU_scaffold68:410871:434490
OMYRGT47TUF
OMM5053
OMM5148 DRE_2:27981593:28018950,OLA_17:30668232:30691497,TNI_unrandom:16885751:16896281,TRU_scaffold80:639048:651505

OmySex

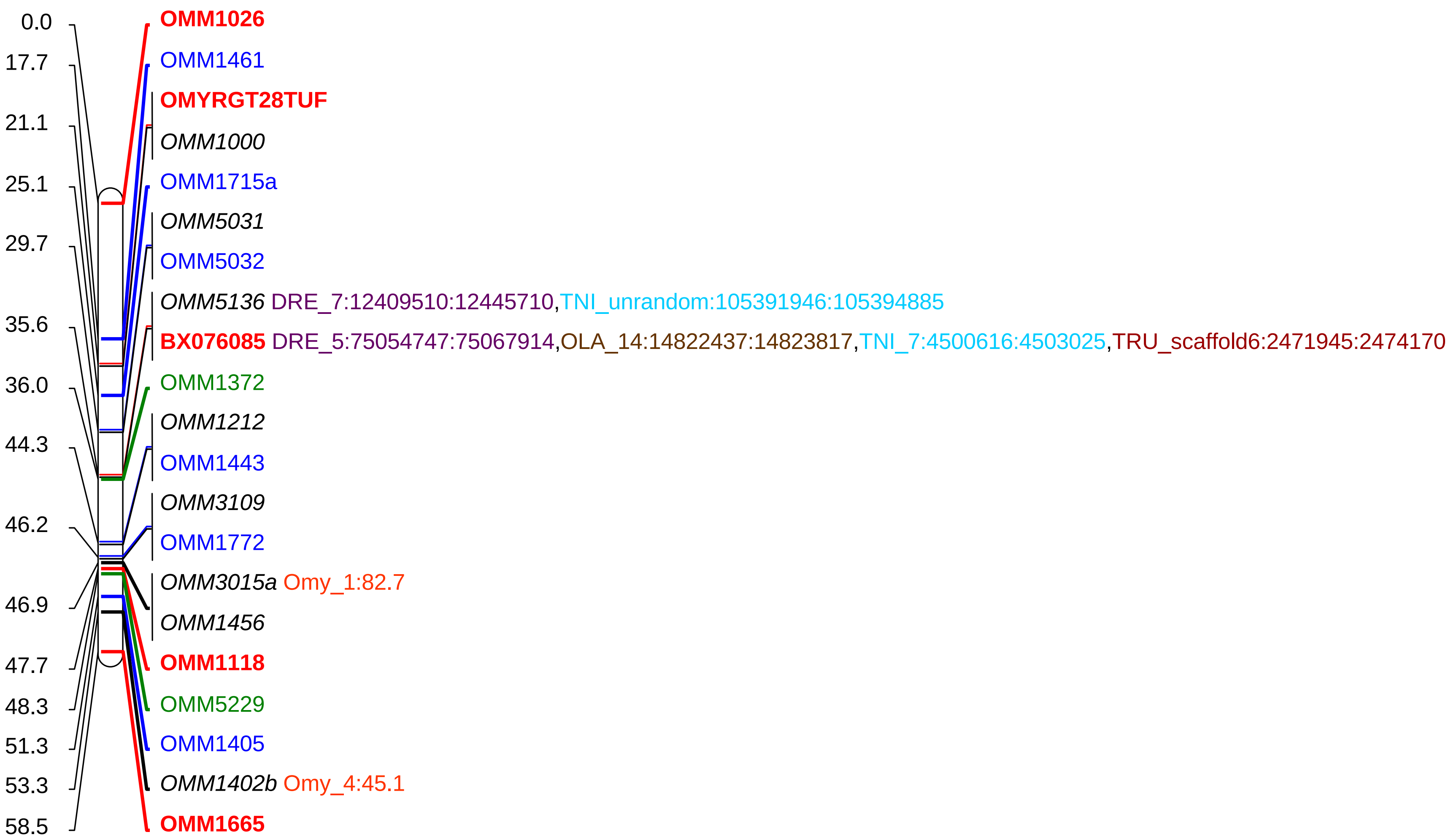
0.0
17.7
21.1
25.1
29.7
35.6
36.0
44.3
46.2
46.9
47.7
48.3
51.3
53.3
58.5
OMM1026
OMM1461
OMYRGT28TUF
OMM1000
OMM1715a
OMM5031
OMM5032
OMM5136 DRE_7:12409510:12445710,TNI_unrandom:105391946:105394885
BX076085 DRE_5:75054747:75067914,OLA_14:14822437:14823817,TNI_7:4500616:4503025,TRU_scaffold6:2471945:2474170
OMM1372
OMM1212
OMM1443
OMM3109
OMM1772
OMM3015a Omy_1:82.7
OMM1456
OMM1118
OMM5229
OMM1405
OMM1402b Omy_4:45.1
OMM1665
