# Supplementary material for: A second generation genetic map for rainbow trout (Oncorhynchus mykiss)
Source: BMC Genet. 2008 Nov 19;9:74. doi: 10.1186/1471-2156-9-74 (PMC2605456; doi:10.1186/1471-2156-9-74)

NCCCWA Genetic Map

OMY1

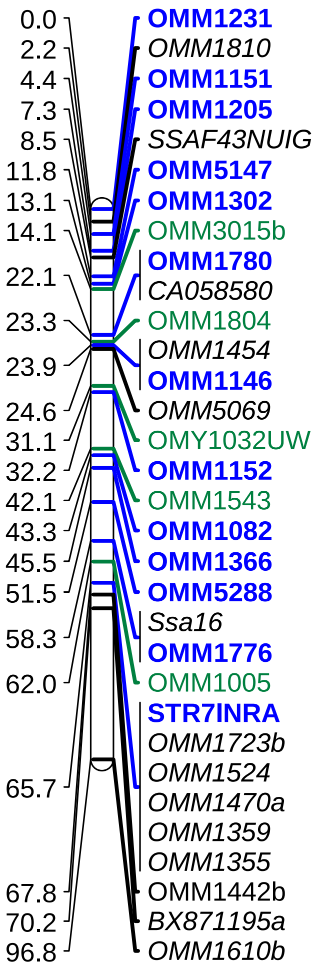

NCCCWA Genetic Map

OMY2

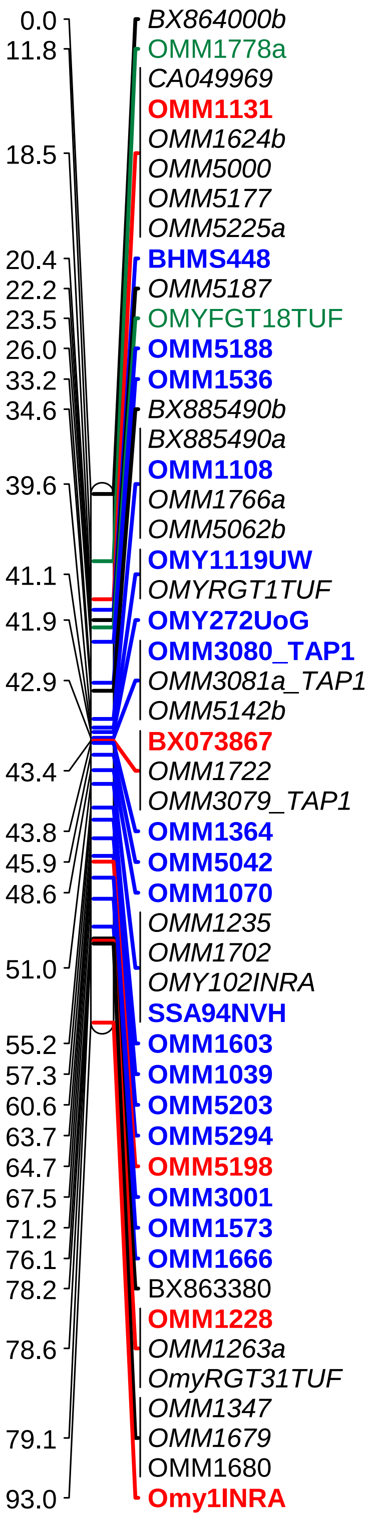

NCCCWA Genetic Map

OMY3

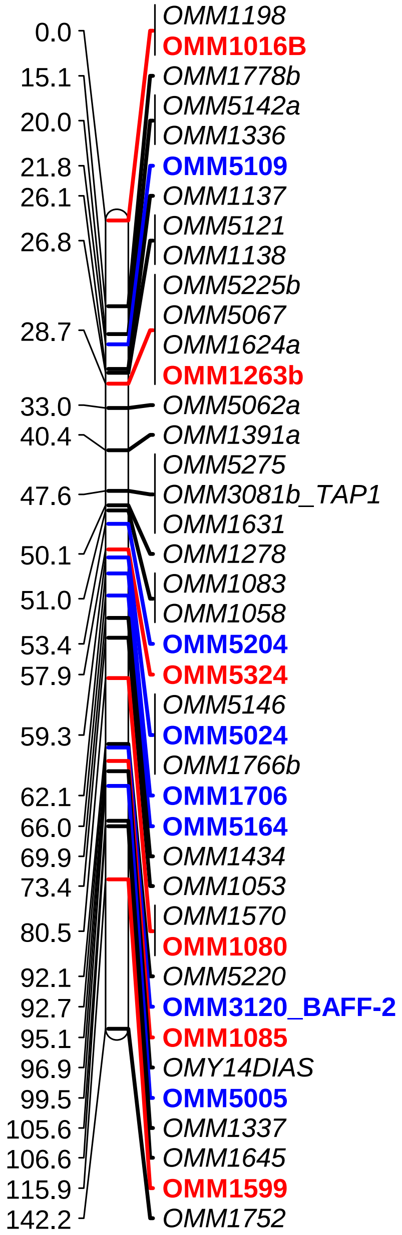

NCCCWA Genetic Map

OMY4

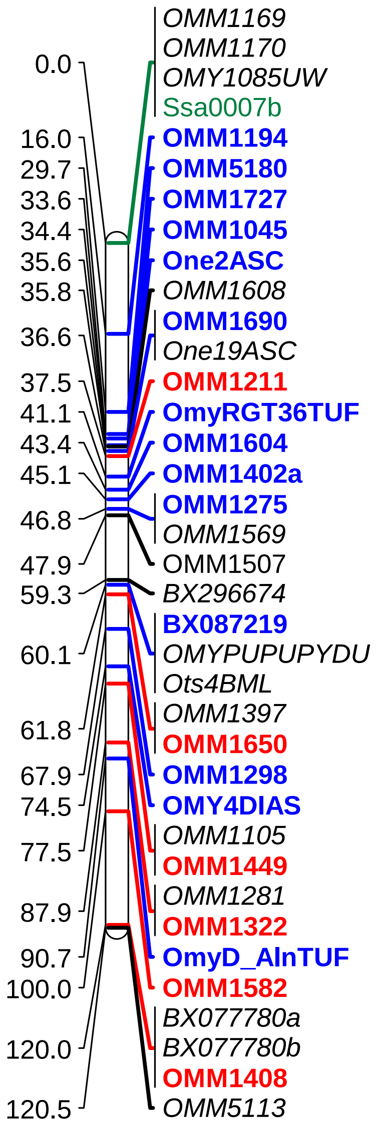

NCCCWA Genetic Map

OMY5

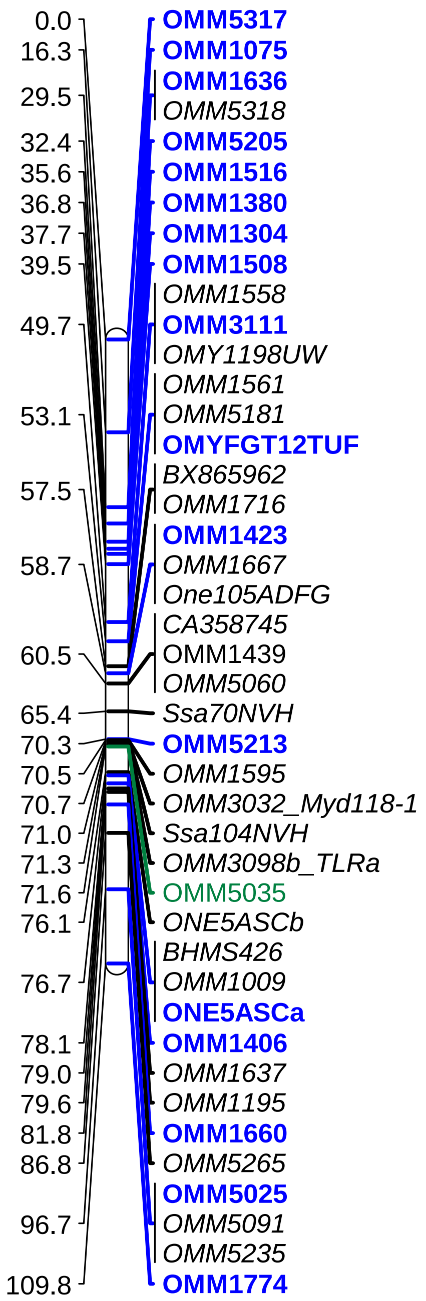

NCCCWA Genetic Map

OMY6

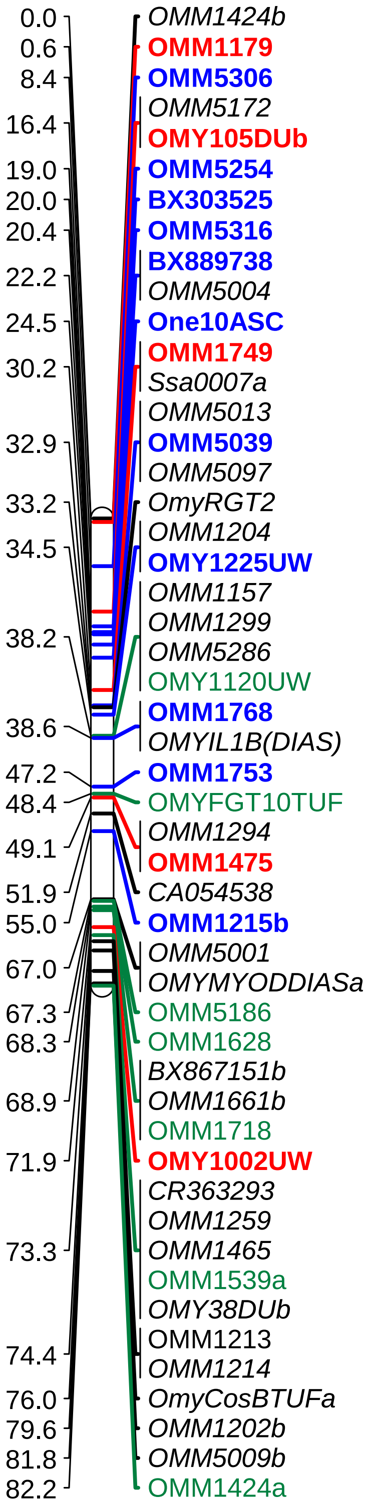

NCCCWA Genetic Map

OMY7

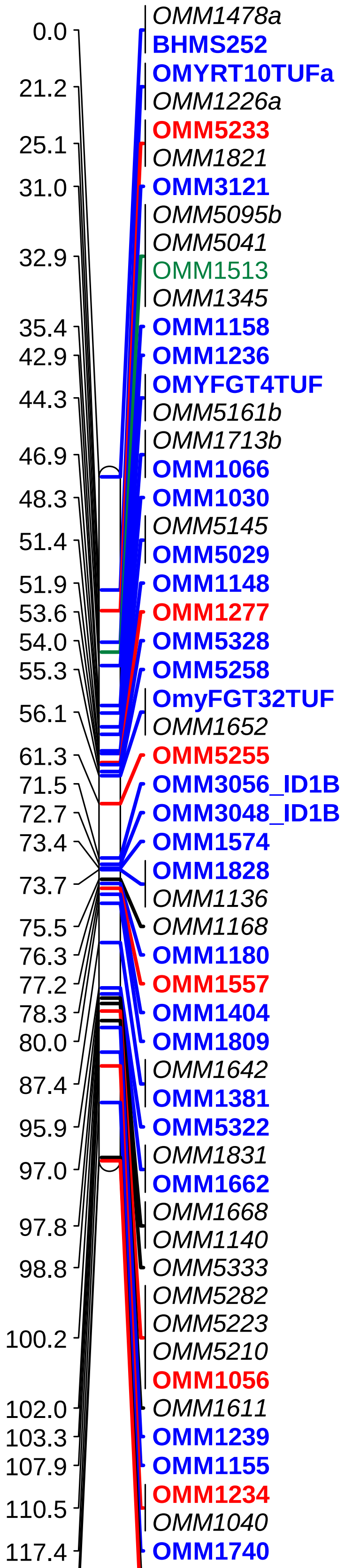

NCCCWA Genetic Map

OMY8

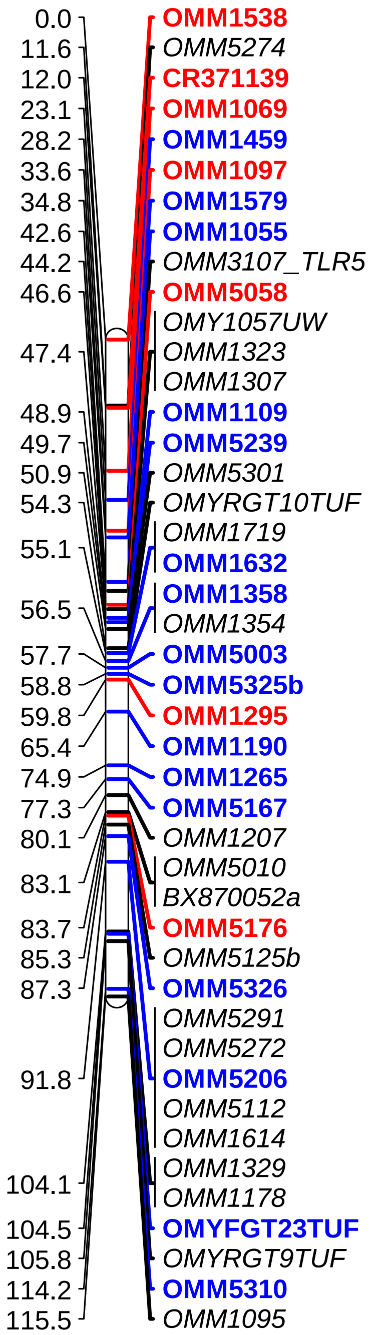

NCCCWA Genetic Map

OMY9

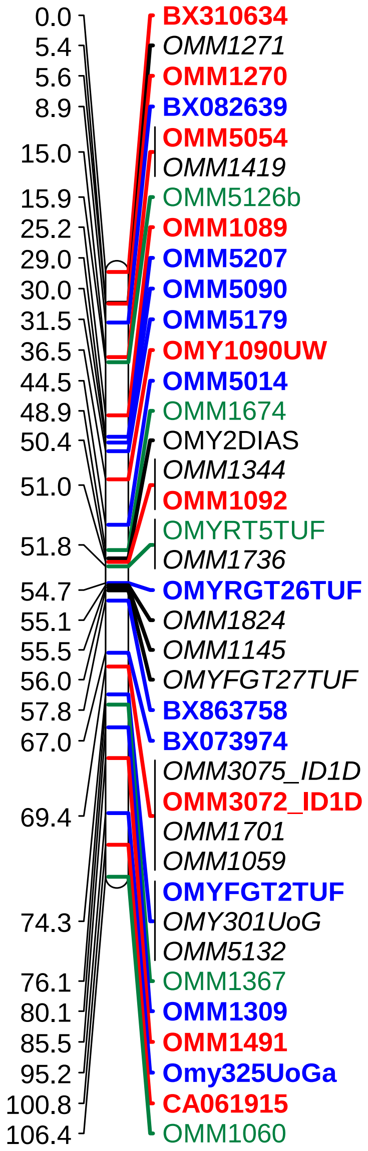

NCCCWA Genetic Map

OMY10

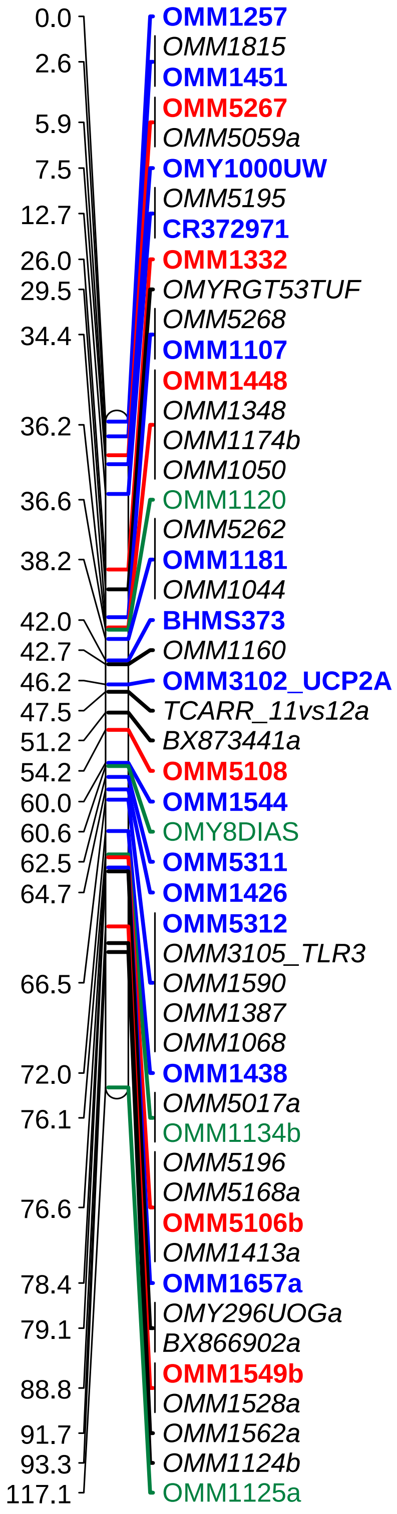

NCCCWA Genetic Map

OMY11

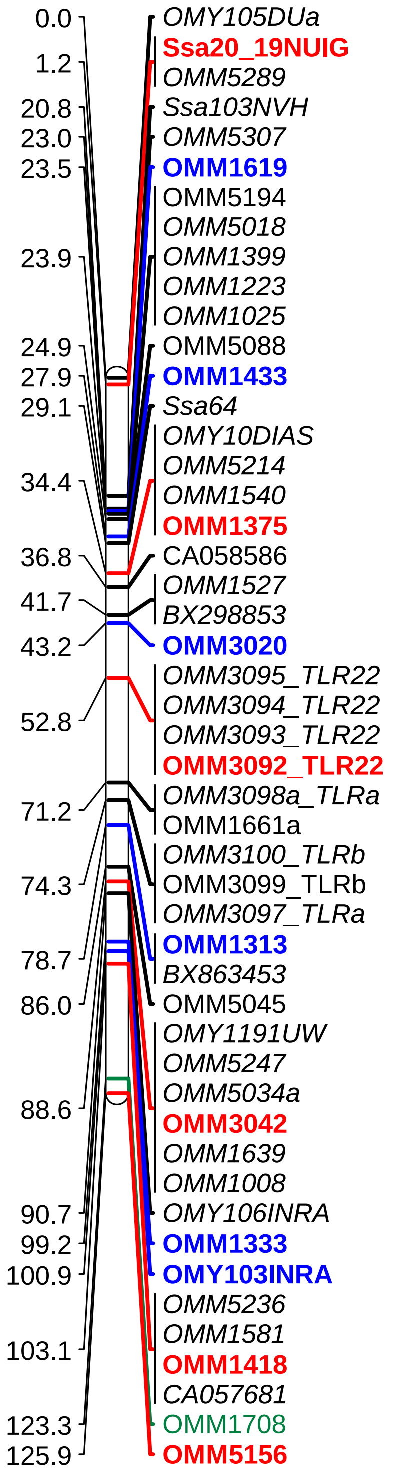

NCCCWA Genetic Map

OMY12

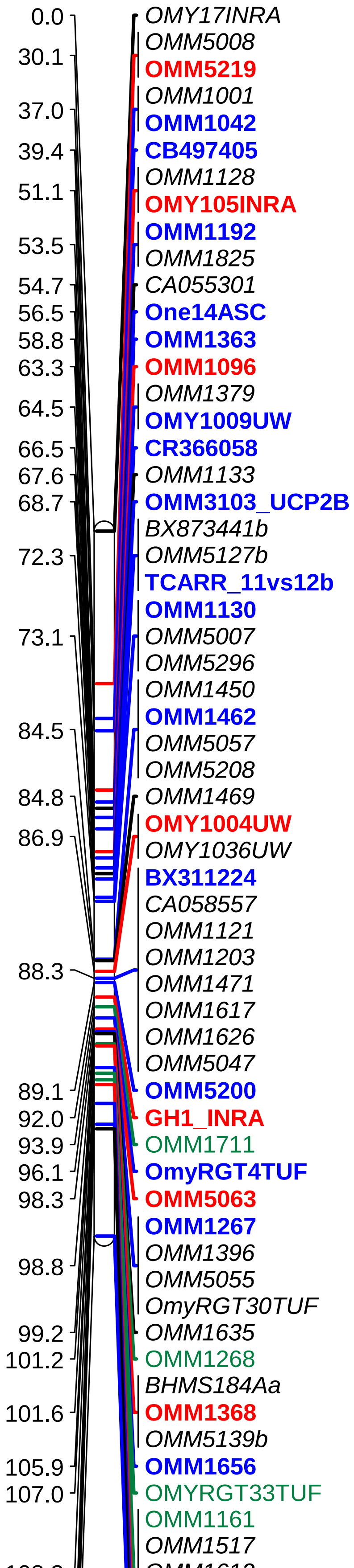

NCCCWA Genetic Map

OMY13

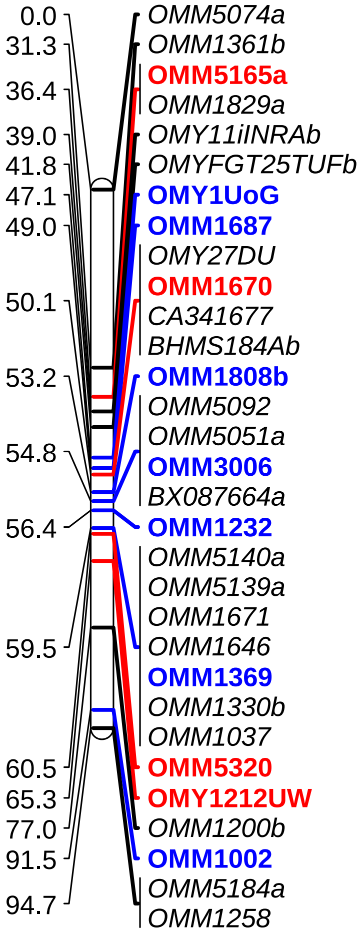

NCCCWA Genetic Map

OMY14

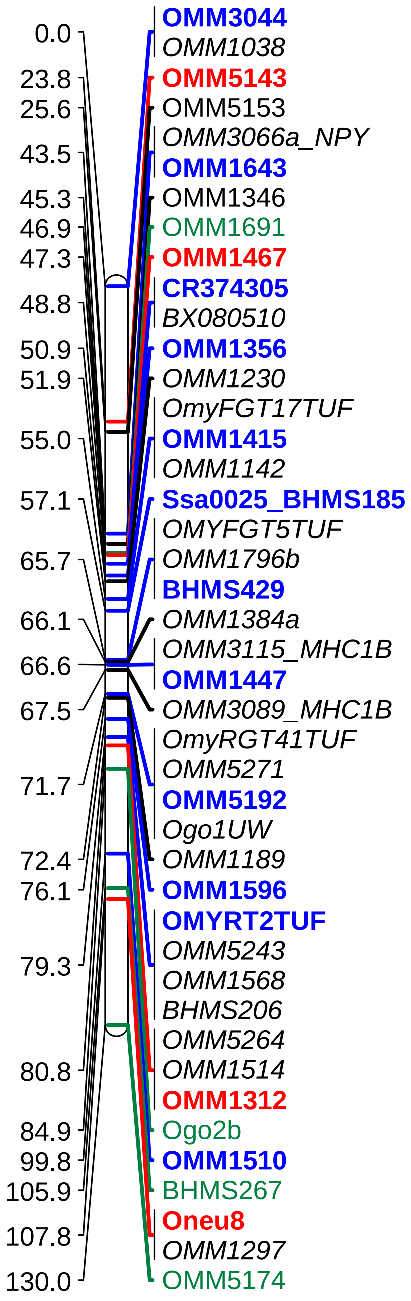

NCCCWA Genetic Map

OMY15

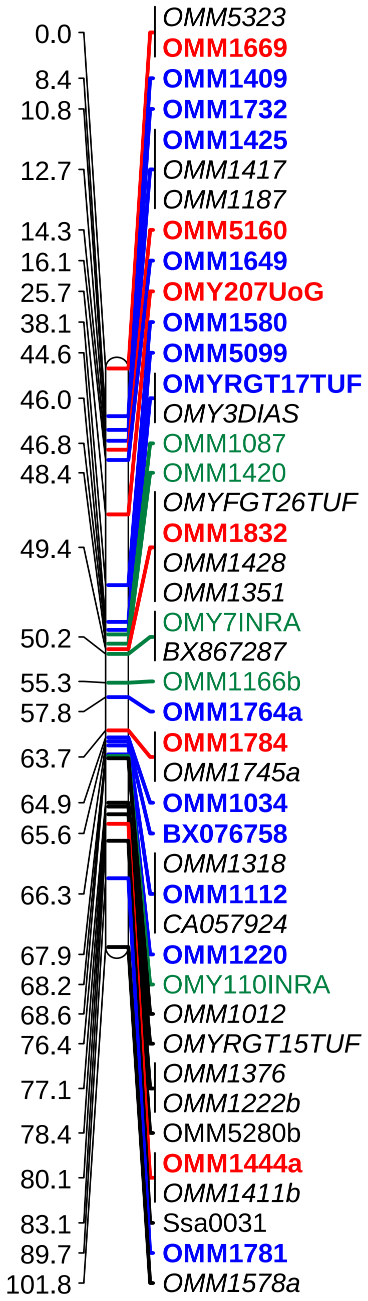

NCCCWA Genetic Map

OMY16

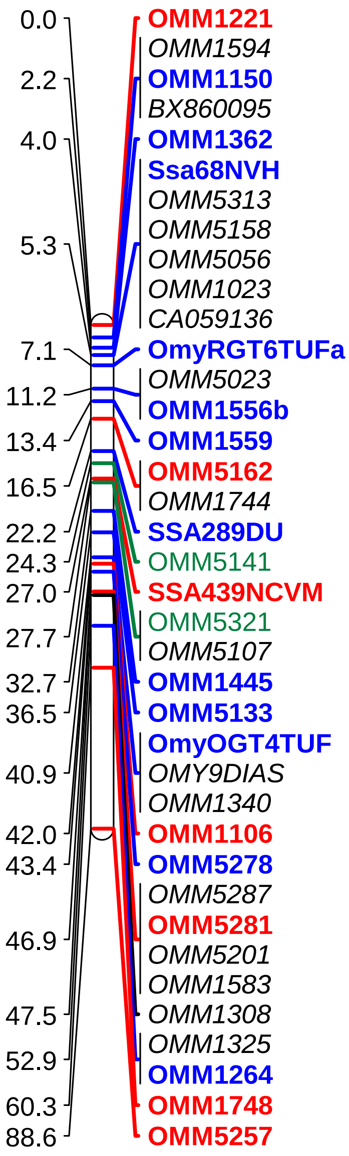

NCCCWA Genetic Map

OMY17

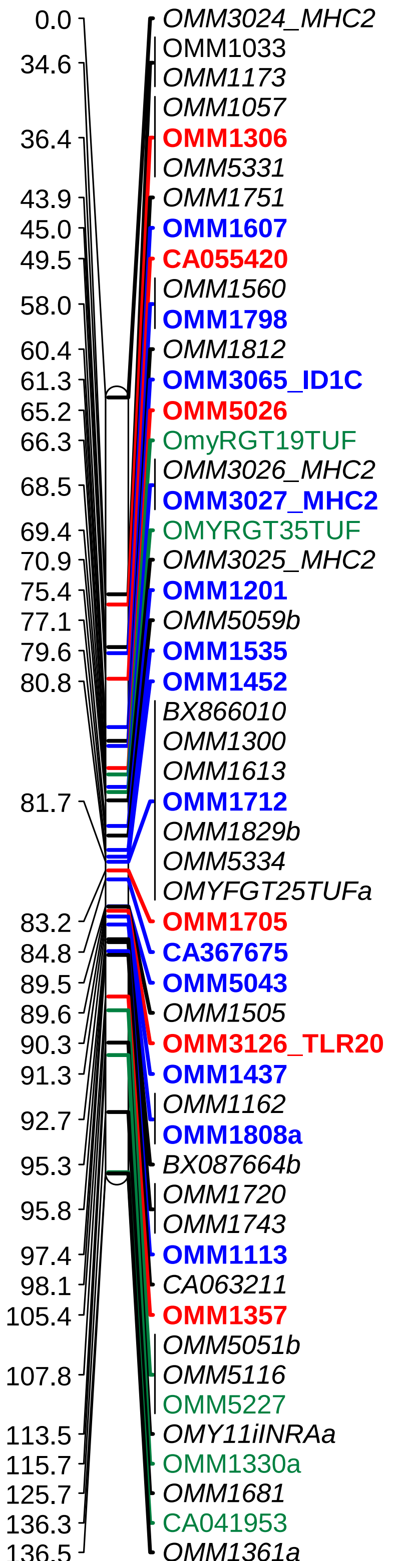

NCCCWA Genetic Map

OMY18

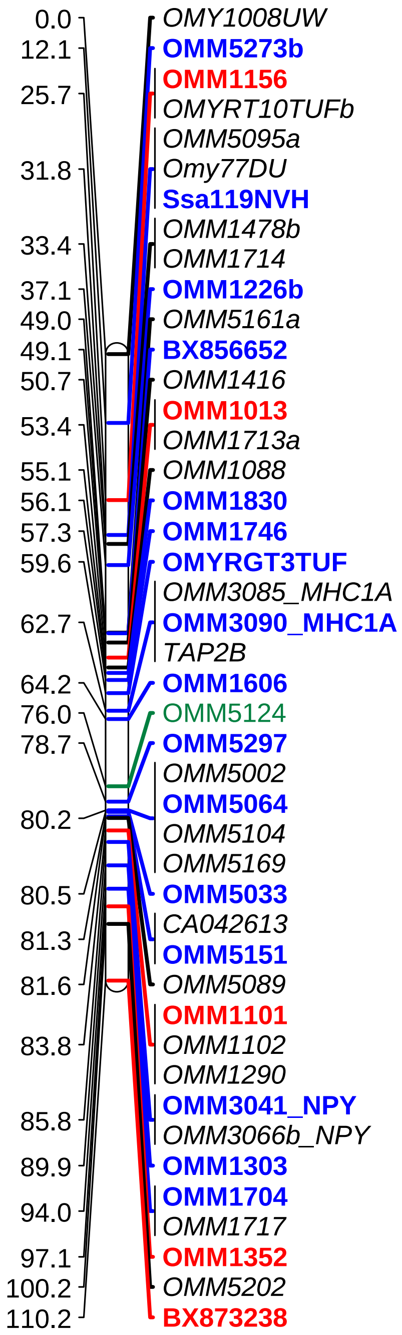

NCCCWA Genetic Map

OMY19

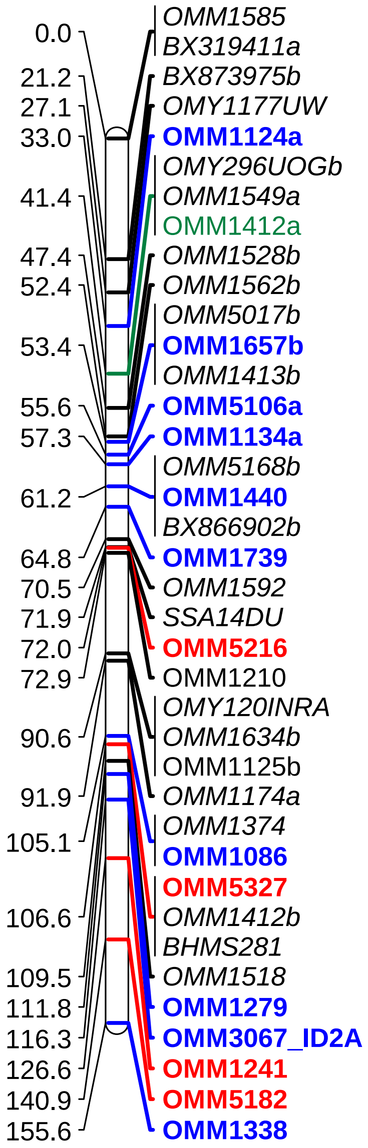

NCCCWA Genetic Map

OMY20

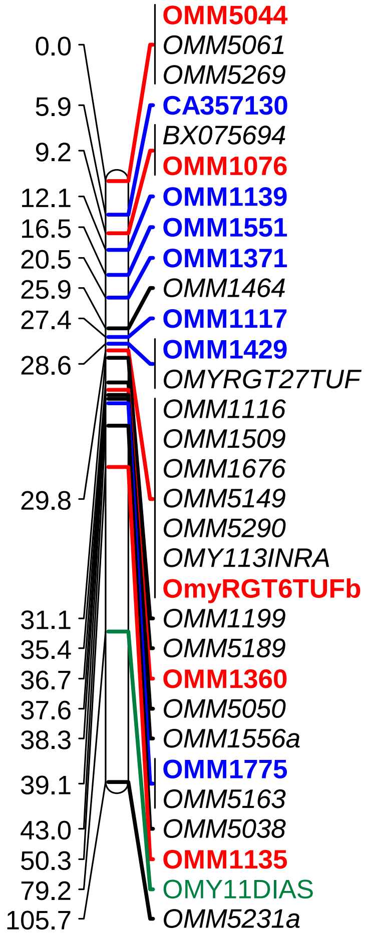

NCCCWA Genetic Map

OMY21

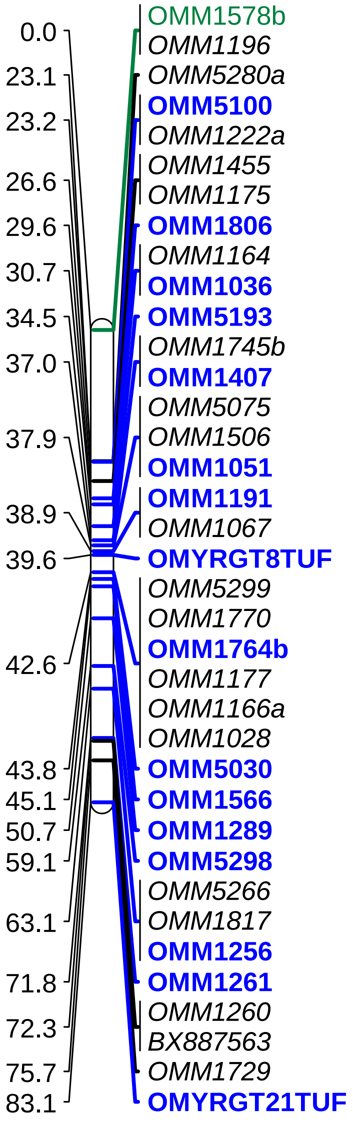

NCCCWA Genetic Map

OMY22

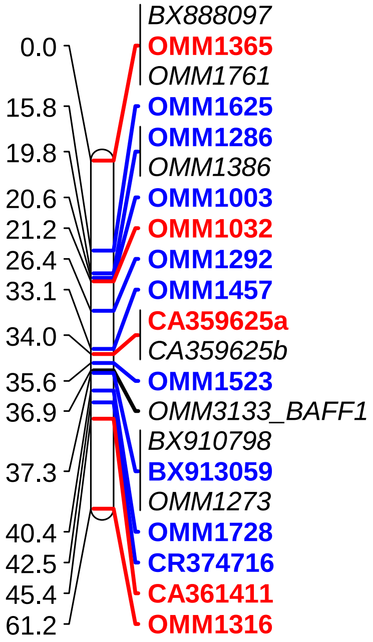

NCCCWA Genetic Map

OMY23

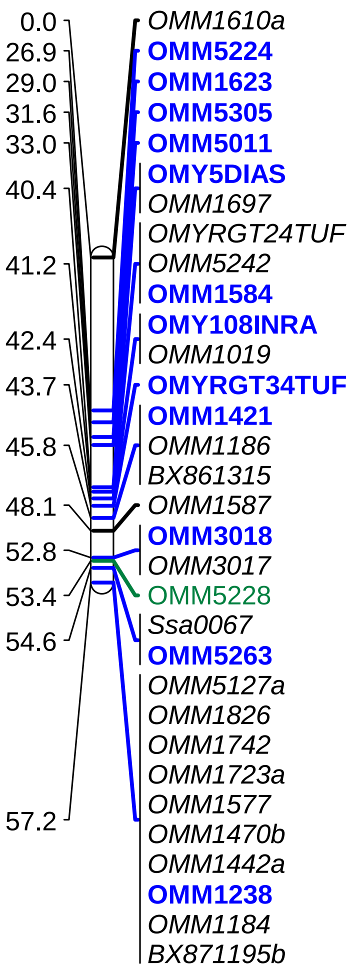

NCCCWA Genetic Map

OMY24

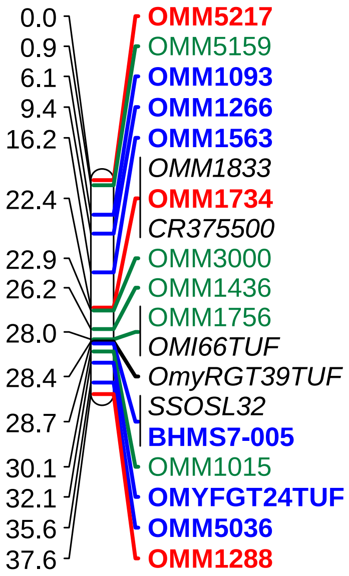

NCCCWA Genetic Map

OMY25

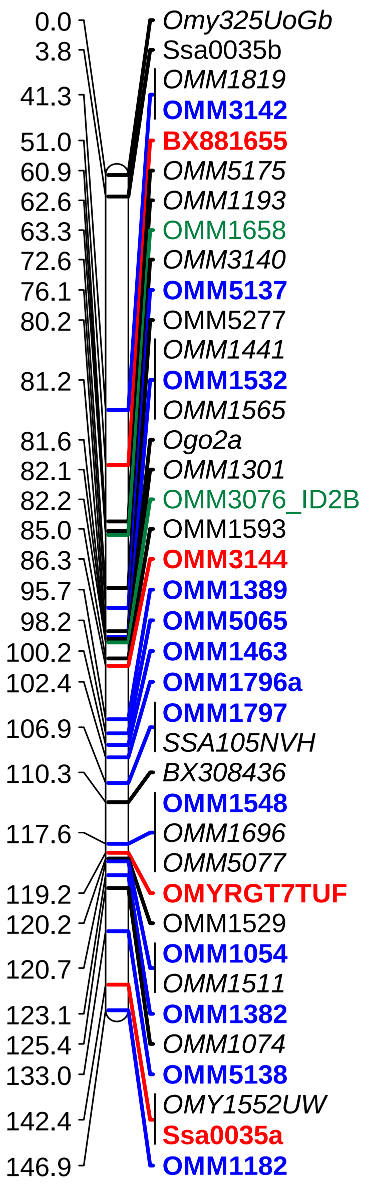

NCCCWA Genetic Map

OMY26

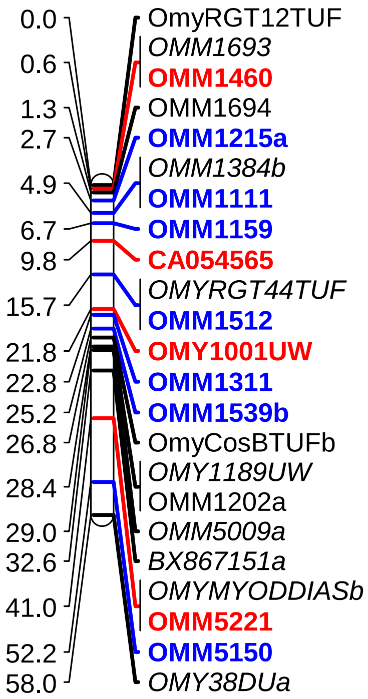

NCCCWA Genetic Map

OMY27

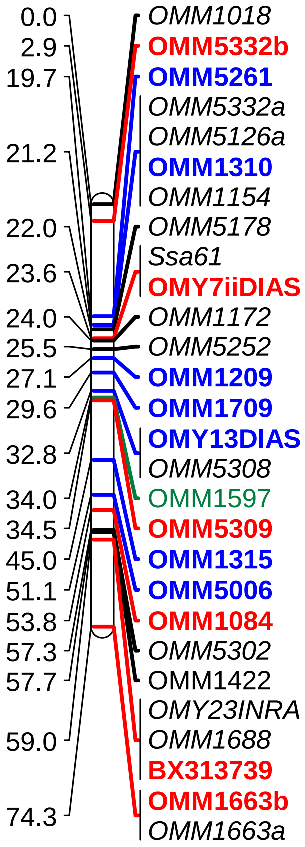

NCCCWA Genetic Map

OMY28

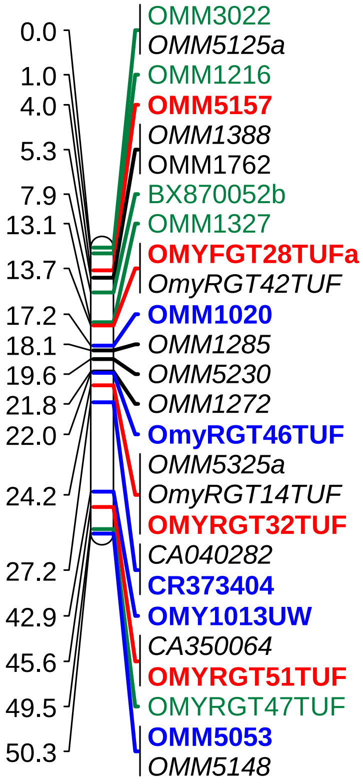

NCCCWA Genetic Map

OMYSex

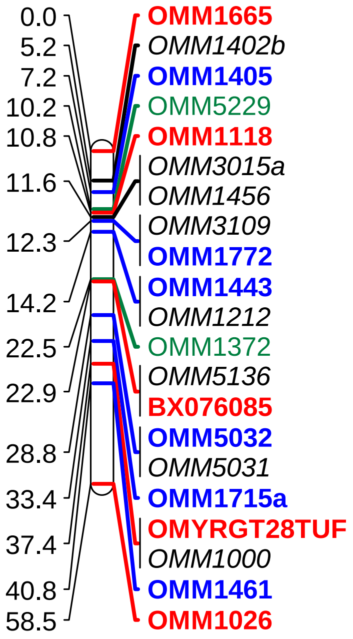

Supplement: Additional file 3 — Genetic Map. This Adobe PDF file includes figures representing the 29 linkage groups/chromosomes of the NCCCWA rainbow trout genetic map. Recombination distances are presented as total Kosambi cM for each map on the left, marker names are on the right. Loci names in red bold font are ordered at LOD 4.0, loci in blue font at LOD 3.0, loci in green font at LOD 2.0, loci in black font at LOD 1.0 and loci in black italic at LOD 0.0. [file 1471-2156-9-74-S3.pdf]
