# Supplementary material for: A second generation genetic map for rainbow trout (Oncorhynchus mykiss)
Source: BMC Genet. 2008 Nov 19;9:74. doi: 10.1186/1471-2156-9-74 (PMC2605456; doi:10.1186/1471-2156-9-74)
Supplement: Additional file 4 — Sex Recombination Ratios. This Adobe PDF file includes figures representing the 29 linkage groups/chromosomes of the NCCCWA rainbow trout genetic map and figures representing the differences in recombination rate between the sexes along each chromosome. The map length is presented in terms of Kosambi cM, the pairwise recombination fractions (theta) between each marker pair along the length of the chromosome map is presented. [file 1471-2156-9-74-S4.pdf]

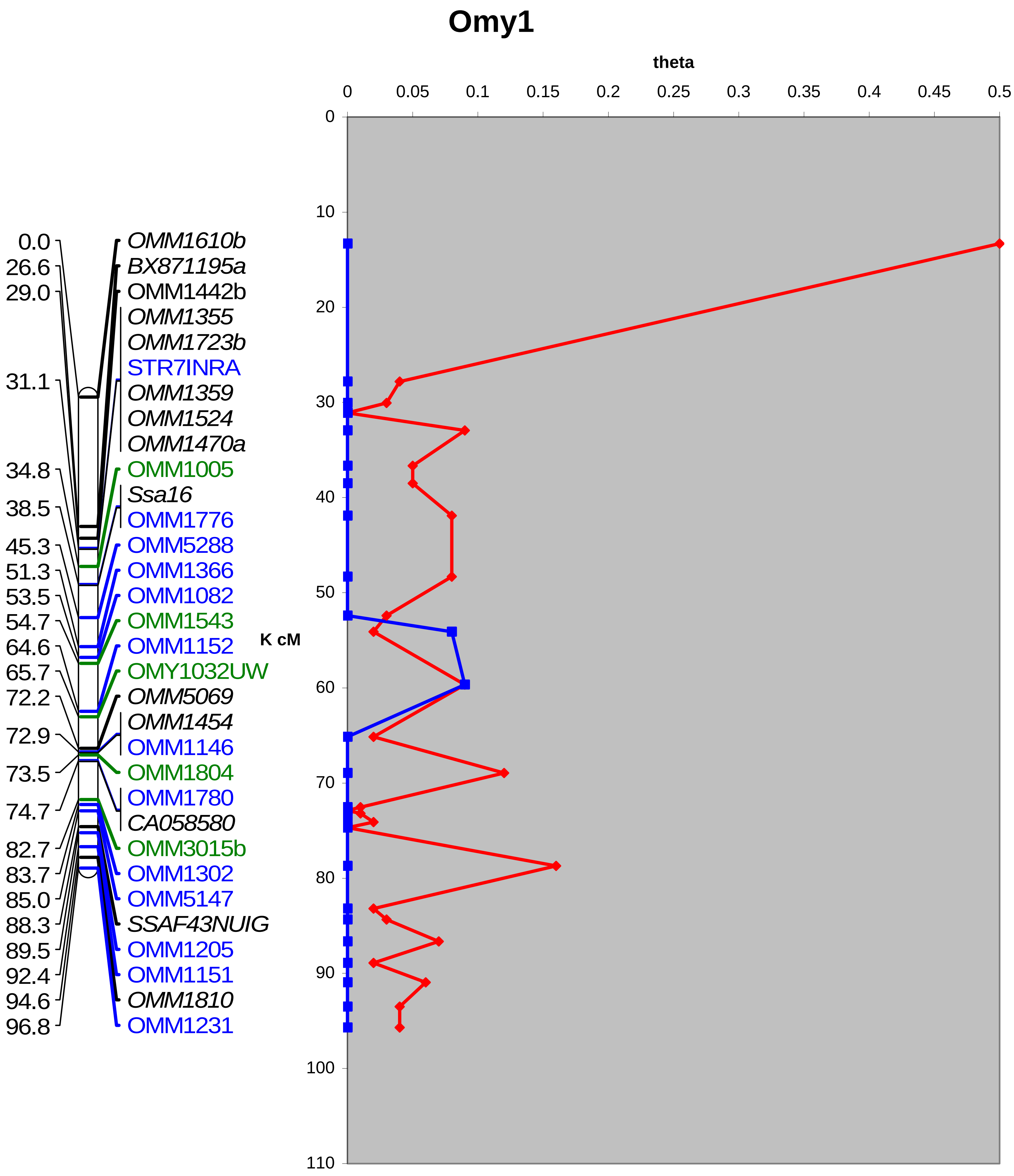
Omy1
theta
0
0.05
0.1
0.15
0.2
0.25
0.3
0.35
0.4
0.45
0.5
0
10
20
30
40
50
60
70
80
90
100
110
K cM
0.0
26.6
29.0
31.1
34.8
38.5
45.3
51.3
53.5
54.7
64.6
65.7
72.2
72.9
73.5
74.7
82.7
83.7
85.0
88.3
89.5
92.4
94.6
96.8
OMM1610b
BX871195a
OMM1442b
OMM1355
OMM1723b
STR7INRA
OMM1359
OMM1524
OMM1470a
OMM1005
Ssa16
OMM1776
OMM5288
OMM1366
OMM1082
OMM1543
OMM1152
OMY1032UW
OMM5069
OMM1454
OMM1146
OMM1804
OMM1780
CA058580
OMM3015b
OMM1302
OMM5147
SSAF43NUIG
OMM1205
OMM1151
OMM1810
OMM1231

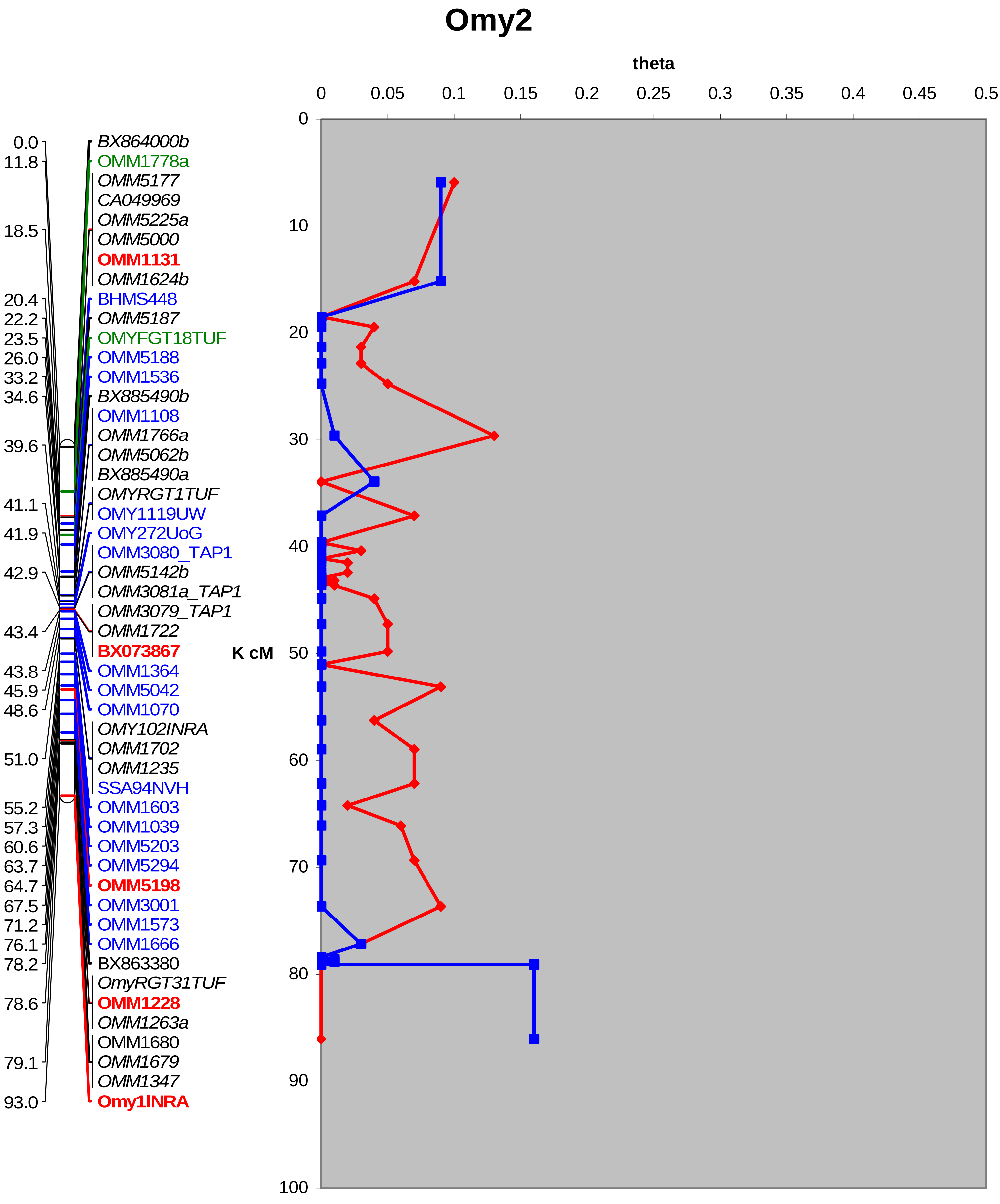
Omy2
theta
0
0.05
0.1
0.15
0.2
0.25
0.3
0.35
0.4
0.45
0.5
K cM
0
10
20
30
40
50
60
70
80
90
100
0.0
11.8
18.5
20.4
22.2
23.5
26.0
33.2
34.6
39.6
41.1
41.9
42.9
43.4
43.8
45.9
48.6
51.0
55.2
57.3
60.6
63.7
64.7
67.5
71.2
76.1
78.2
78.6
79.1
93.0
BX864000b
OMM1778a
OMM5177
CA049969
OMM5225a
OMM5000
OMM1131
OMM1624b
BHMS448
OMM5187
OMYFGT18TUF
OMM5188
OMM1536
BX885490b
OMM1108
OMM1766a
OMM5062b
BX885490a
OMYRGT1TUF
OMY1119UW
OMY272UoG
OMM3080_TAP1
OMM5142b
OMM3081a_TAP1
OMM3079_TAP1
OMM1722
BX073867
OMM1364
OMM5042
OMM1070
OMY102INRA
OMM1702
OMM1235
SSA94NVH
OMM1603
OMM1039
OMM5203
OMM5294
OMM5198
OMM3001
OMM1573
OMM1666
BX863380
OmyRGT31TUF
OMM1228
OMM1263a
OMM1680
OMM1679
OMM1347
Omy1INRA

Omy3

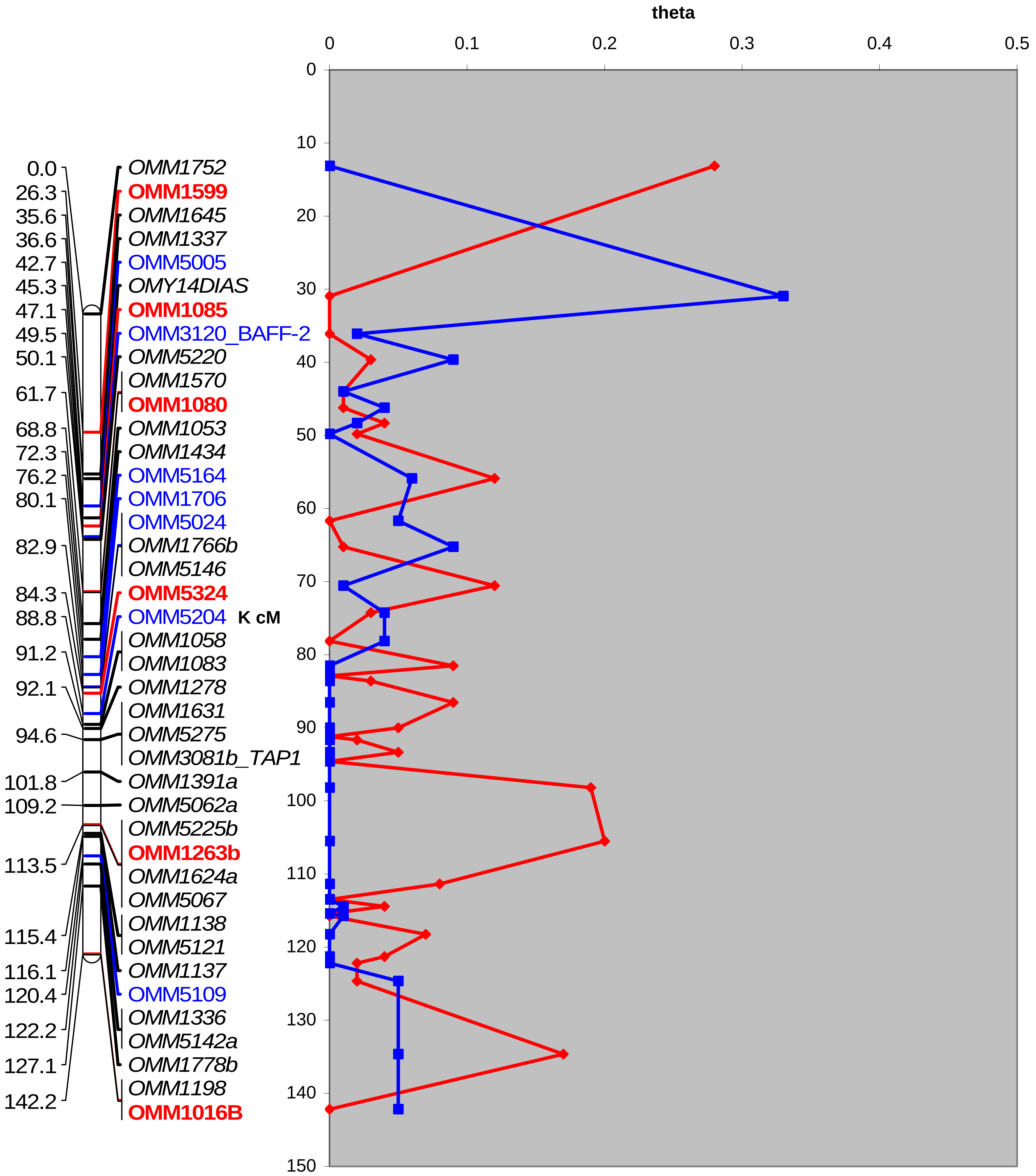

theta
0
0.1
0.2
0.3
0.4
0.5
0
10
20
30
40
50
60
70
80
90
100
110
120
130
140
150
K cM
0.0
26.3
35.6
36.6
42.7
45.3
47.1
49.5
50.1
61.7
68.8
72.3
76.2
80.1
82.9
84.3
88.8
91.2
92.1
94.6
101.8
109.2
113.5
115.4
116.1
120.4
122.2
127.1
142.2
OMM1752
OMM1599
OMM1645
OMM1337
OMM5005
OMY14DIAS
OMM1085
OMM3120_BAFF-2
OMM5220
OMM1570
OMM1080
OMM1053
OMM1434
OMM5164
OMM1706
OMM5024
OMM1766b
OMM5146
OMM5324
OMM5204
OMM1058
OMM1083
OMM1278
OMM1631
OMM5275
OMM3081b_TAP1
OMM1391a
OMM5062a
OMM5225b
OMM1263b
OMM1624a
OMM5067
OMM1138
OMM5121
OMM1137
OMM5109
OMM1336
OMM5142a
OMM1778b
OMM1198
OMM1016B

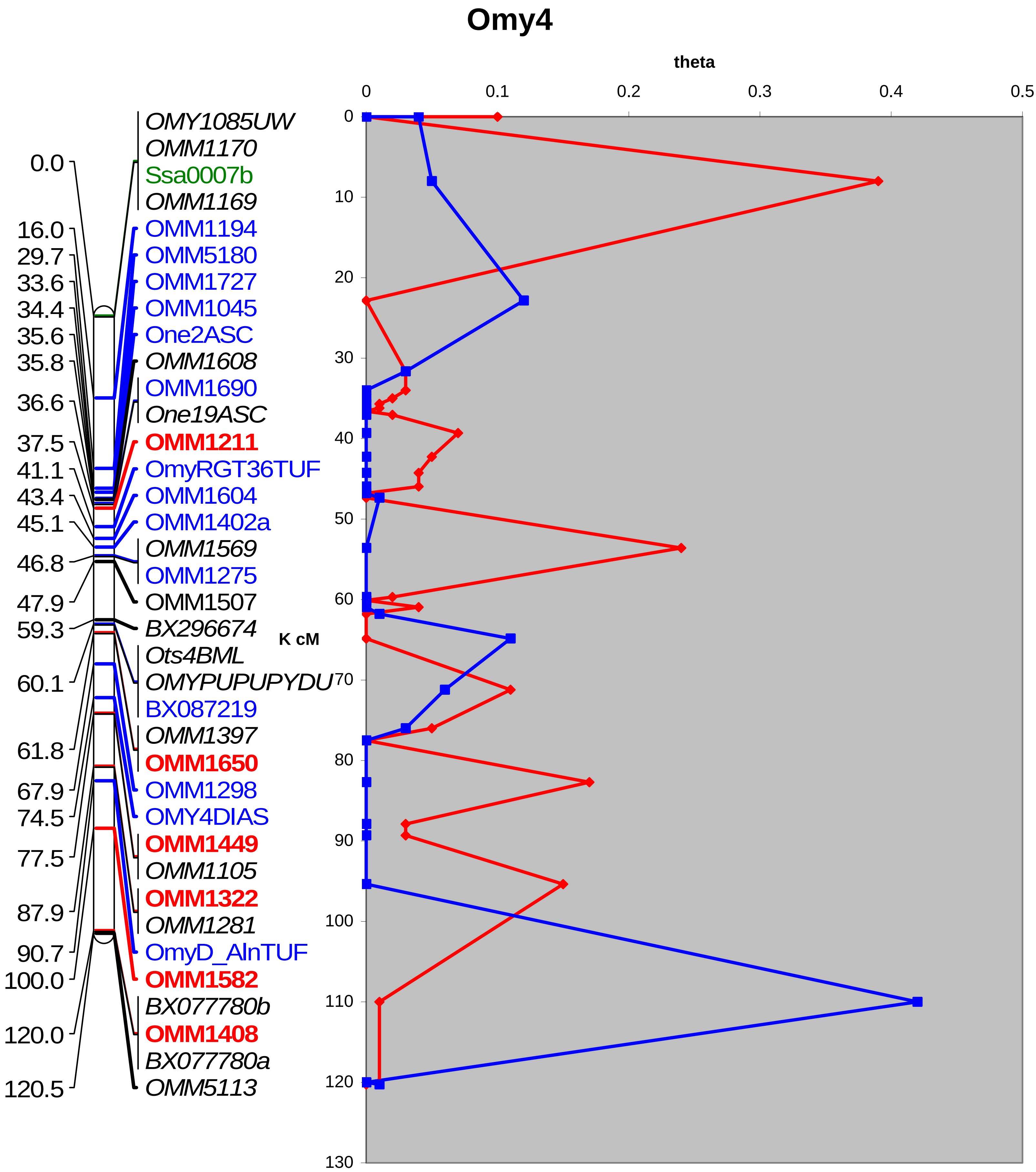

Omy4
theta
0
0.1
0.2
0.3
0.4
0.5
K cM
0
10
20
30
40
50
60
70
80
90
100
110
120
130
0.0
16.0
29.7
33.6
34.4
35.6
35.8
36.6
37.5
41.1
43.4
45.1
46.8
47.9
59.3
60.1
61.8
67.9
74.5
77.5
87.9
90.7
100.0
120.0
120.5
OMY1085UW
OMM1170
Ssa0007b
OMM1169
OMM1194
OMM5180
OMM1727
OMM1045
One2ASC
OMM1608
OMM1690
One19ASC
OMM1211
OmyRGT36TUF
OMM1604
OMM1402a
OMM1569
OMM1275
OMM1507
BX296674
Ots4BML
OMYPUPUPYDU
BX087219
OMM1397
OMM1650
OMM1298
OMY4DIAS
OMM1449
OMM1105
OMM1322
OMM1281
OmyD_AlnTUF
OMM1582
BX077780b
OMM1408
BX077780a
OMM5113

Omy5

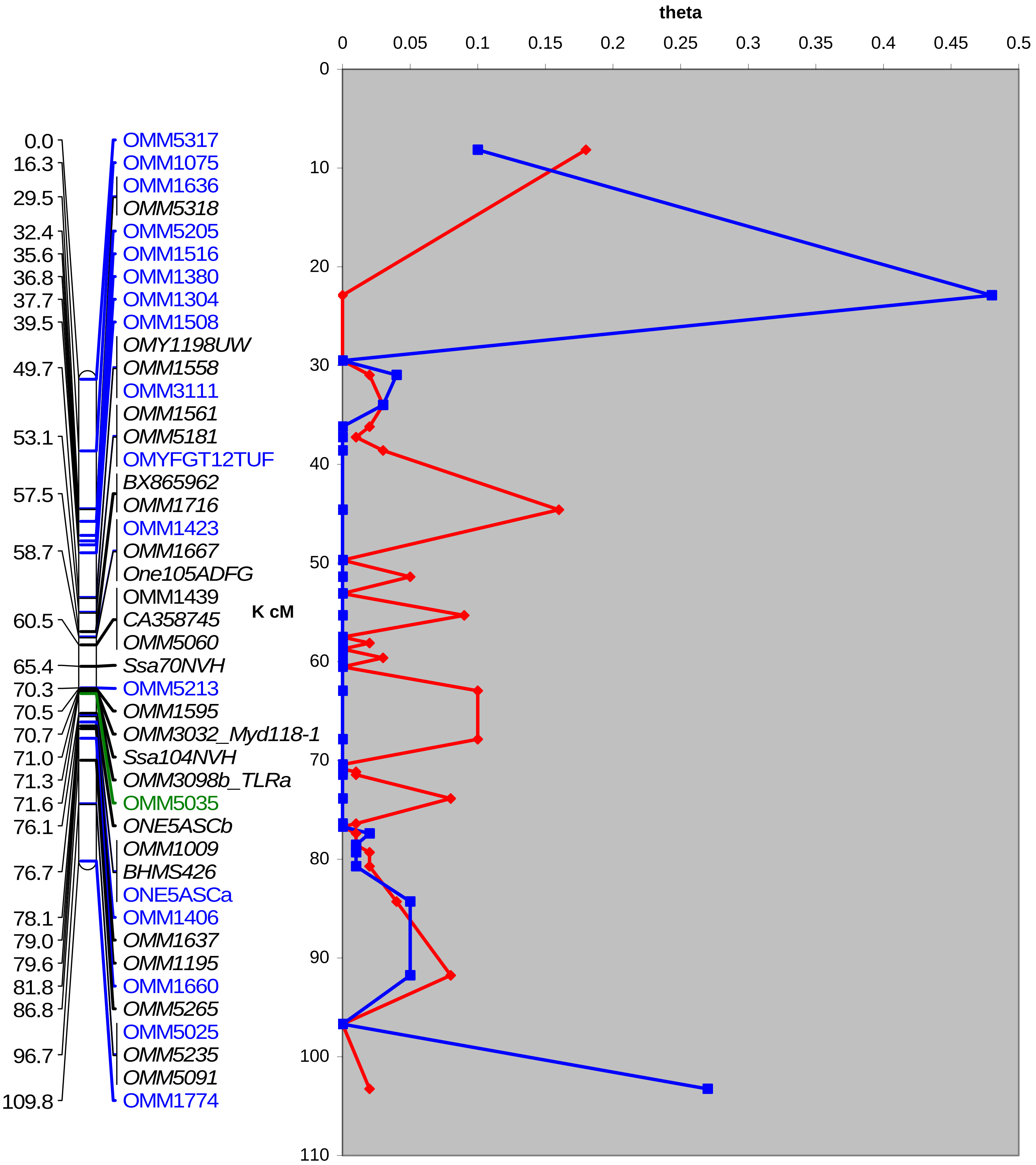

theta
0
0.05
0.1
0.15
0.2
0.25
0.3
0.35
0.4
0.45
0.5
0
10
20
30
40
50
60
70
80
90
100
110
K cM
0.0
16.3
29.5
32.4
35.6
36.8
37.7
39.5
49.7
53.1
57.5
58.7
60.5
65.4
70.3
70.5
70.7
71.0
71.3
71.6
76.1
76.7
78.1
79.0
79.6
81.8
86.8
96.7
109.8
OMM5317
OMM1075
OMM1636
OMM5318
OMM5205
OMM1516
OMM1380
OMM1304
OMM1508
OMY1198UW
OMM1558
OMM3111
OMM1561
OMM5181
OMYFGT12TUF
BX865962
OMM1716
OMM1423
OMM1667
One105ADFG
OMM1439
CA358745
OMM5060
Ssa70NVH
OMM5213
OMM1595
OMM3032_Myd118-1
Ssa104NVH
OMM3098b_TLRa
OMM5035
ONE5ASCb
OMM1009
BHMS426
ONE5ASCa
OMM1406
OMM1637
OMM1195
OMM1660
OMM5265
OMM5025
OMM5235
OMM5091
OMM1774

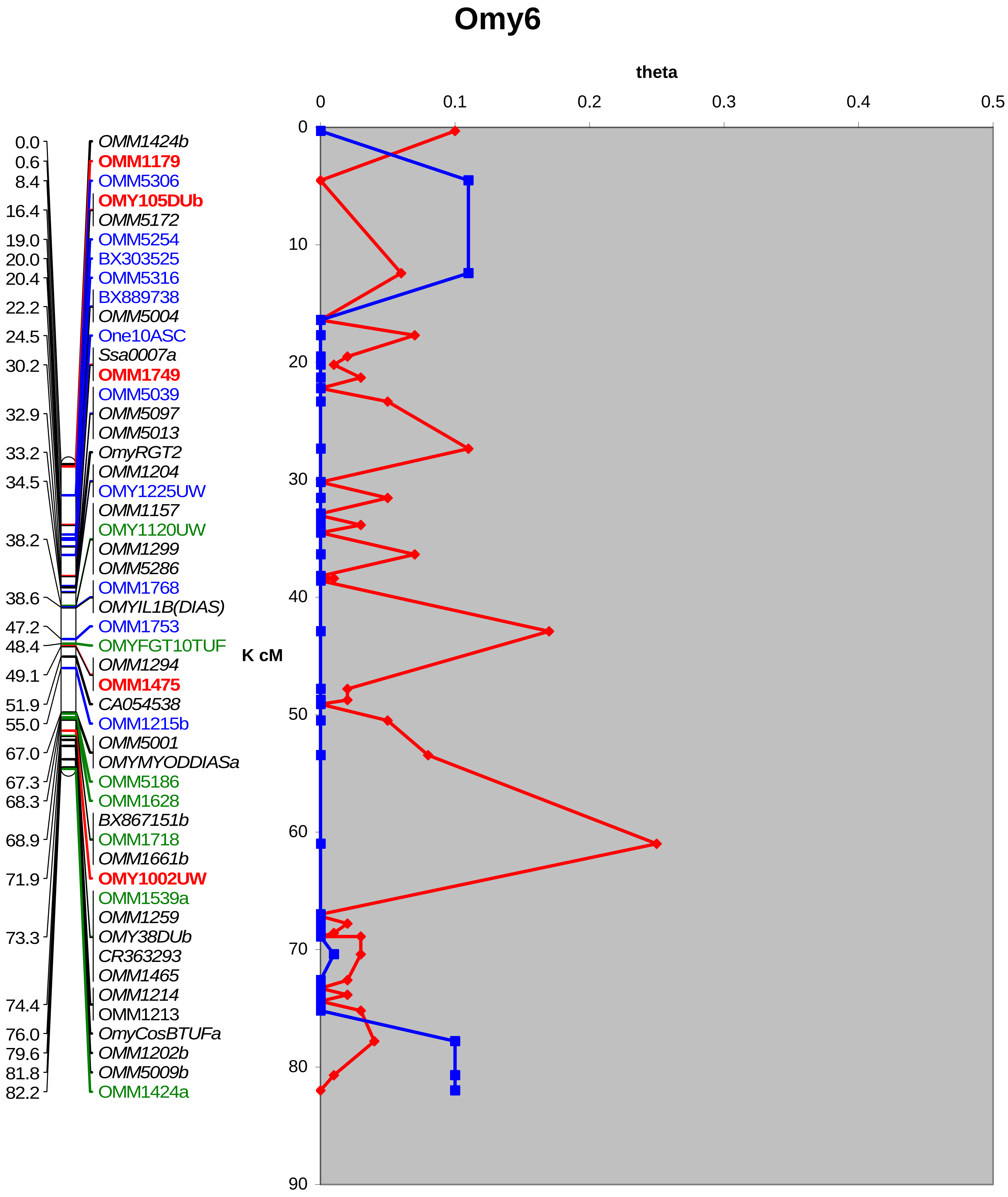
Omy6
theta
0
0.1
0.2
0.3
0.4
0.5
K cM
0
10
20
30
40
50
60
70
80
90
0.0
0.6
8.4
16.4
19.0
20.0
20.4
22.2
24.5
30.2
32.9
33.2
34.5
38.2
38.6
47.2
48.4
49.1
51.9
55.0
67.0
67.3
68.3
68.9
71.9
73.3
74.4
76.0
79.6
81.8
82.2
OMM1424b
OMM1179
OMM5306
OMY105DUb
OMM5172
OMM5254
BX303525
OMM5316
BX889738
OMM5004
One10ASC
Ssa0007a
OMM1749
OMM5039
OMM5097
OMM5013
OmyRGT2
OMM1204
OMY1225UW
OMM1157
OMY1120UW
OMM1299
OMM5286
OMM1768
OMYIL1B(DIAS)
OMM1753
OMYFGT10TUF
OMM1294
OMM1475
CA054538
OMM1215b
OMM5001
OMYMYODDIASa
OMM5186
OMM1628
BX867151b
OMM1718
OMM1661b
OMY1002UW
OMM1539a
OMM1259
OMY38DUb
CR363293
OMM1465
OMM1214
OMM1213
OmyCosBTUFa
OMM1202b
OMM5009b
OMM1424a

Omy7

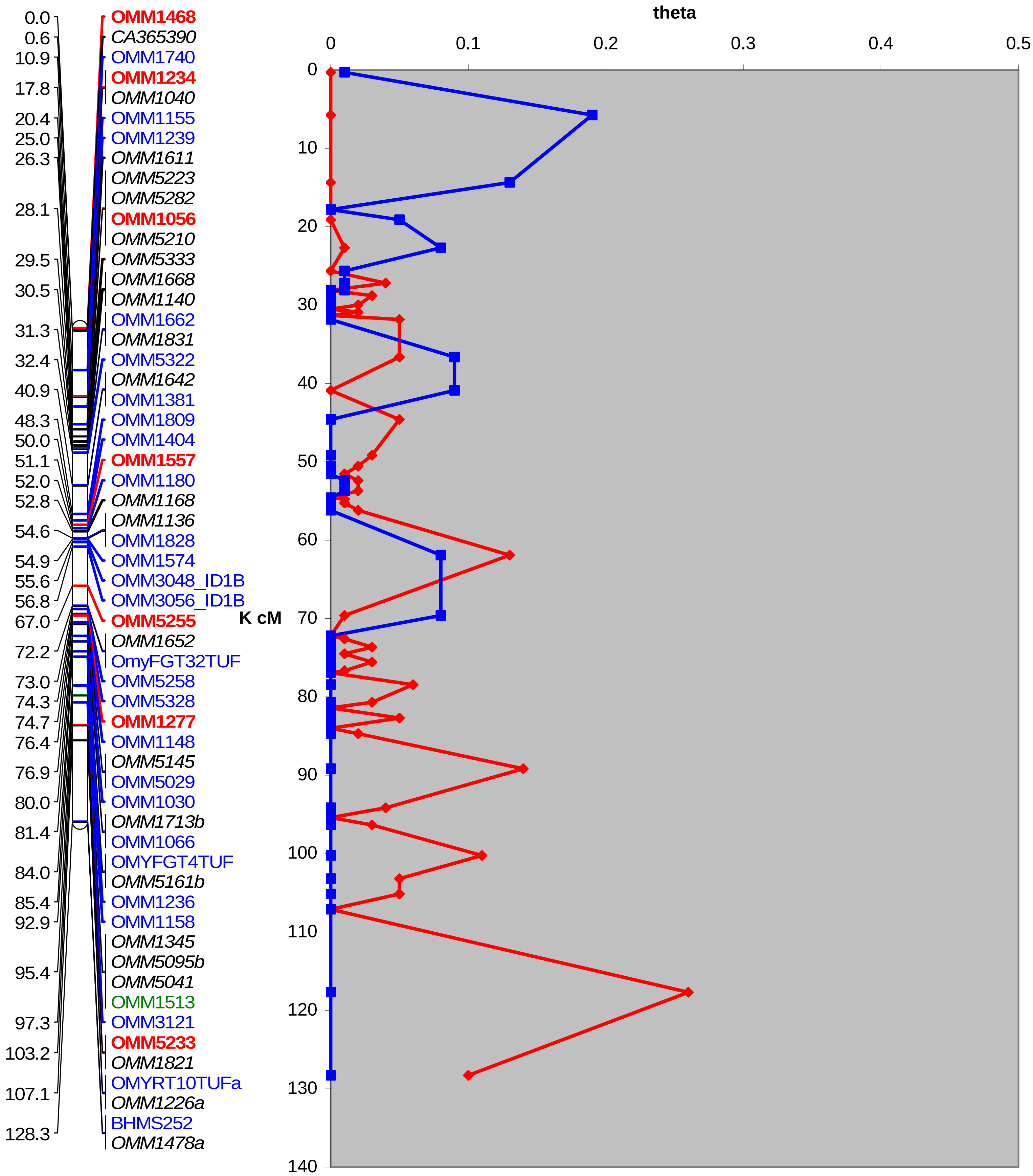
theta
K cM
0.0 OMM1468
0.6 CA365390
10.9 OMM1740
17.8 OMM1234
OMM1040
20.4 OMM1155
25.0 OMM1239
26.3 OMM1611
OMM5223
OMM5282
28.1 OMM1056
OMM5210
29.5 OMM5333
30.5 OMM1668
OMM1140
31.3 OMM1662
OMM1831
32.4 OMM5322
OMM1642
40.9 OMM1381
48.3 OMM1809
50.0 OMM1404
51.1 OMM1557
52.0 OMM1180
52.8 OMM1168
OMM1136
54.6 OMM1828
54.9 OMM1574
55.6 OMM3048_ID1B
56.8 OMM3056_ID1B
67.0 OMM5255
OMM1652
72.2 OmyFGT32TUF
73.0 OMM5258
74.3 OMM5328
74.7 OMM1277
76.4 OMM1148
OMM5145
76.9 OMM5029
80.0 OMM1030
OMM1713b
81.4 OMM1066
OMYFGT4TUF
84.0 OMM5161b
85.4 OMM1236
92.9 OMM1158
OMM1345
95.4 OMM5095b
OMM5041
OMM1513
97.3 OMM3121
OMM5233
103.2 OMM1821
OMYRT10TUFa
107.1 OMM1226a
BHMS252
128.3 OMM1478a
0 0.1 0.2 0.3 0.4 0.5
0 10 20 30 40 50 60 70 80 90 100 110 120 130 140

Omy8

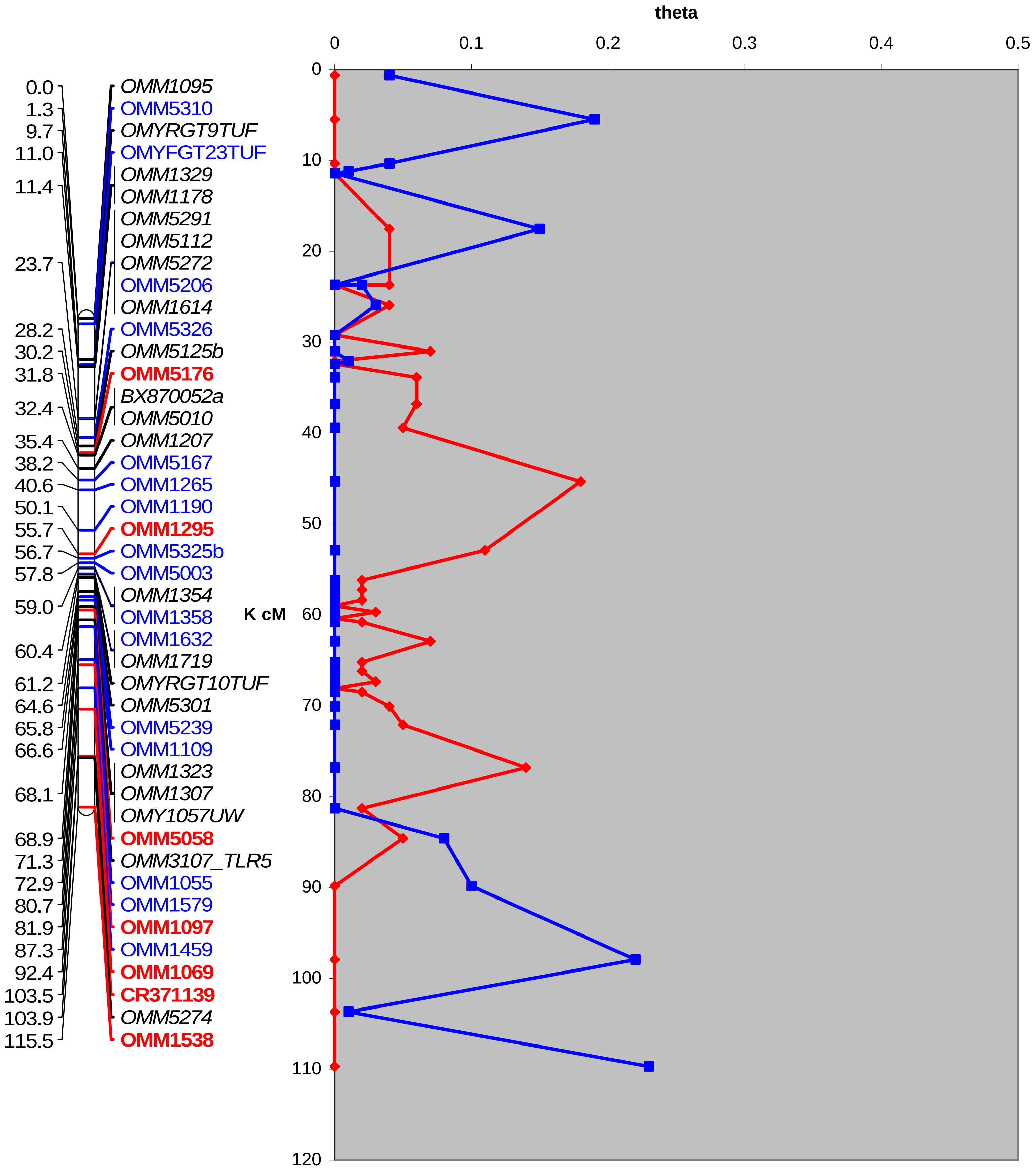

0.0
1.3
9.7
11.0
11.4
23.7
28.2
30.2
31.8
32.4
35.4
38.2
40.6
50.1
55.7
56.7
57.8
59.0
60.4
61.2
64.6
65.8
66.6
68.1
68.9
71.3
72.9
80.7
81.9
87.3
92.4
103.5
103.9
115.5
OMM1095
OMM5310
OMYRGT9TUF
OMYFGT23TUF
OMM1329
OMM1178
OMM5291
OMM5112
OMM5272
OMM5206
OMM1614
OMM5326
OMM5125b
OMM5176
BX870052a
OMM5010
OMM1207
OMM5167
OMM1265
OMM1190
OMM1295
OMM5325b
OMM5003
OMM1354
OMM1358
OMM1632
OMM1719
OMYRGT10TUF
OMM5301
OMM5239
OMM1109
OMM1323
OMM1307
OMY1057UW
OMM5058
OMM3107_TLR5
OMM1055
OMM1579
OMM1097
OMM1459
OMM1069
CR371139
OMM5274
OMM1538
K cM
theta
0
0.1
0.2
0.3
0.4
0.5
0
10
20
30
40
50
60
70
80
90
100
110
120

Omy9

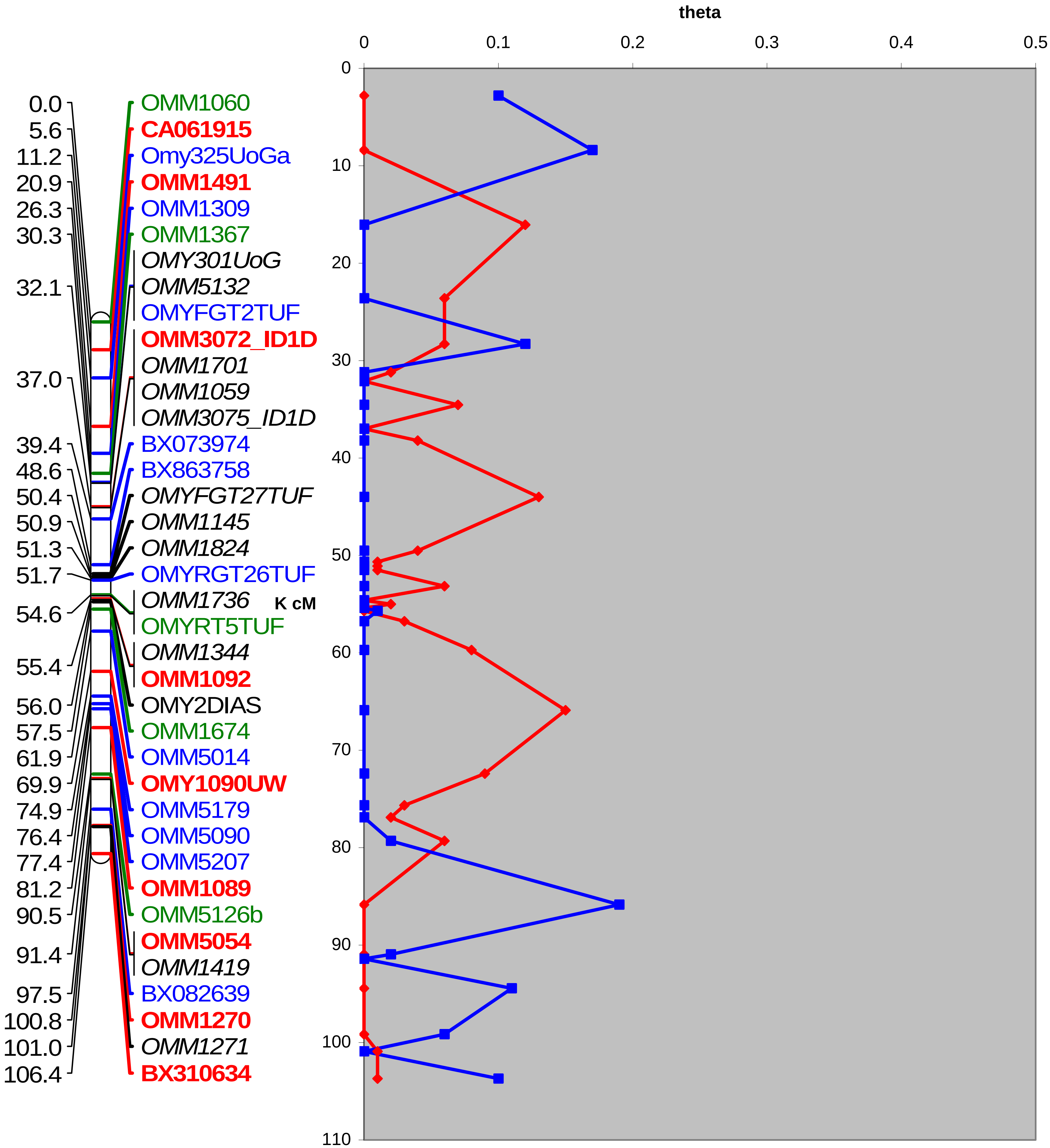

0.0 OMM1060
5.6 CA061915
11.2 Omy325UoGa
20.9 OMM1491
26.3 OMM1309
30.3 OMM1367
OMY301UoG
32.1 OMM5132
OMYFGT2TUF
OMM3072_ID1D
OMM1701
37.0 OMM1059
OMM3075_ID1D
39.4 BX073974
48.6 BX863758
50.4 OMYFGT27TUF
50.9 OMM1145
51.3 OMM1824
51.7 OMYRGT26TUF
OMM1736
54.6 OMYRT5TUF
OMM1344
55.4 OMM1092
56.0 OMY2DIAS
57.5 OMM1674
61.9 OMM5014
69.9 OMY1090UW
74.9 OMM5179
76.4 OMM5090
77.4 OMM5207
81.2 OMM1089
90.5 OMM5126b
OMM5054
91.4 OMM1419
97.5 BX082639
100.8 OMM1270
101.0 OMM1271
106.4 BX310634
K cM
theta
0 0.1 0.2 0.3 0.4 0.5
0 10 20 30 40 50 60 70 80 90 100 110

Omy10

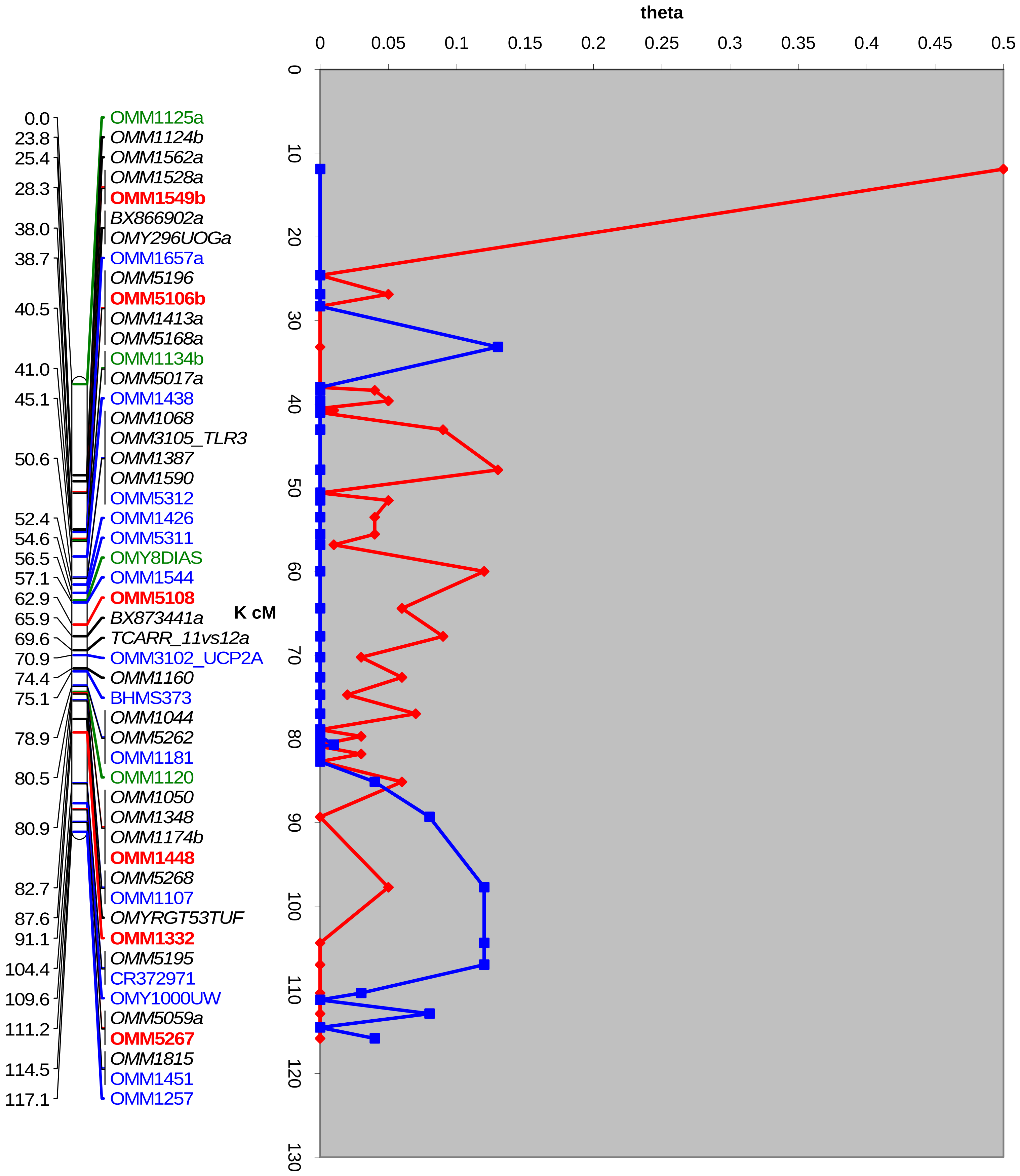

0.0
23.8
25.4
28.3
38.0
38.7
40.5
41.0
45.1
50.6
52.4
54.6
56.5
57.1
62.9
65.9
69.6
70.9
74.4
75.1
78.9
80.5
80.9
82.7
87.6
91.1
104.4
109.6
111.2
114.5
117.1
OMM1125a
OMM1124b
OMM1562a
OMM1528a
OMM1549b
BX866902a
OMY296UOGa
OMM1657a
OMM5196
OMM5106b
OMM1413a
OMM5168a
OMM1134b
OMM5017a
OMM1438
OMM1068
OMM3105_TLR3
OMM1387
OMM1590
OMM5312
OMM1426
OMM5311
OMY8DIAS
OMM1544
OMM5108
BX873441a
TCARR_11vs12a
OMM3102_UCP2A
OMM1160
BHMS373
OMM1044
OMM5262
OMM1181
OMM1120
OMM1050
OMM1348
OMM1174b
OMM1448
OMM5268
OMM1107
OMYRGT53TUF
OMM1332
OMM5195
CR372971
OMY1000UW
OMM5059a
OMM5267
OMM1815
OMM1451
OMM1257
K cM
theta
0
0.05
0.1
0.15
0.2
0.25
0.3
0.35
0.4
0.45
0.5
0
10
20
30
40
50
60
70
80
90
100
110
120
130

Omy11

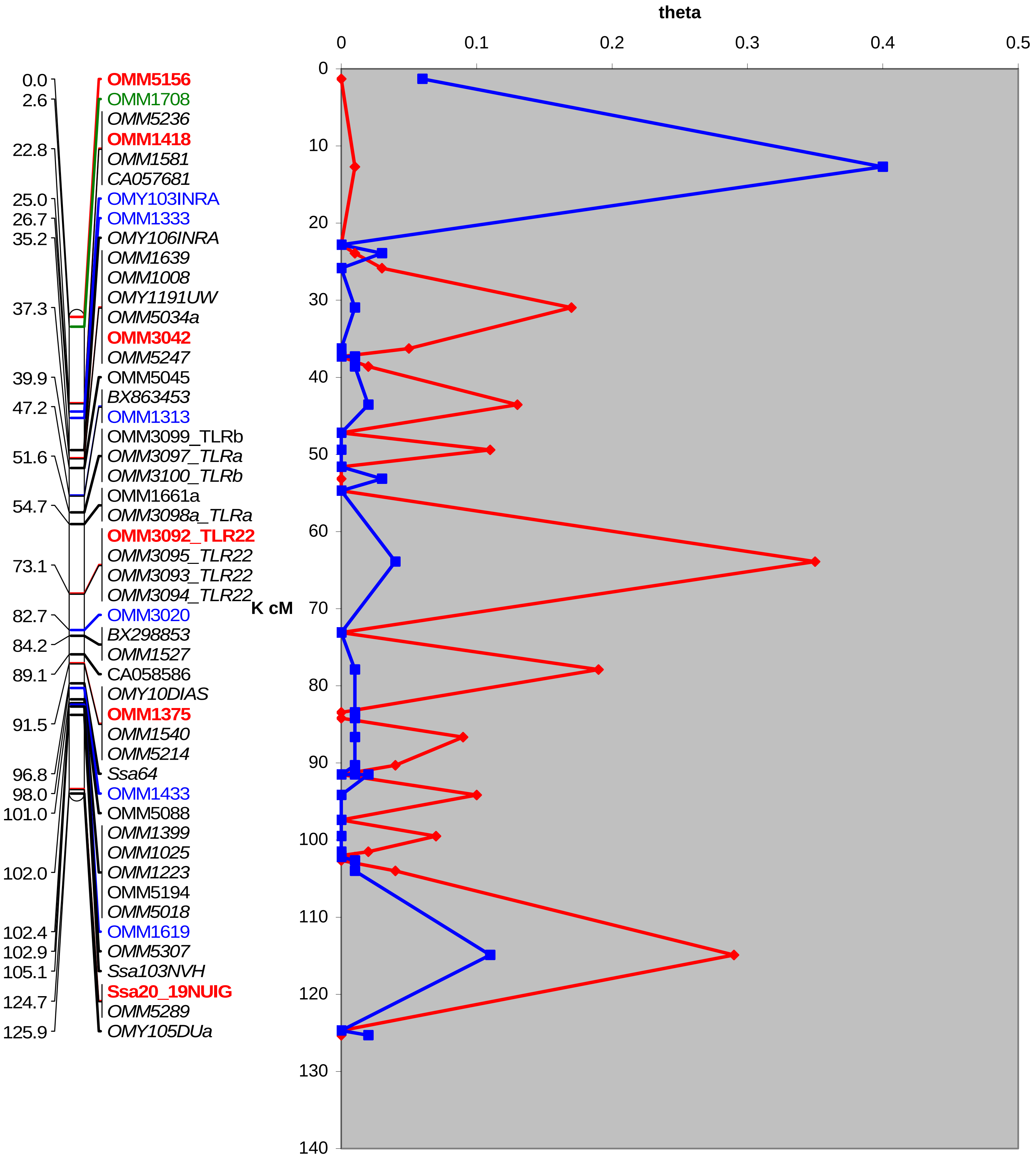

0.0
2.6
22.8
25.0
26.7
35.2
37.3
39.9
47.2
51.6
54.7
73.1
82.7
84.2
89.1
91.5
96.8
98.0
101.0
102.0
102.4
102.9
105.1
124.7
125.9
OMM5156
OMM1708
OMM5236
OMM1418
OMM1581
CA057681
OMY103INRA
OMM1333
OMY106INRA
OMM1639
OMM1008
OMY1191UW
OMM5034a
OMM3042
OMM5247
OMM5045
BX863453
OMM1313
OMM3099_TLRb
OMM3097_TLRa
OMM3100_TLRb
OMM1661a
OMM3098a_TLRa
OMM3092_TLR22
OMM3095_TLR22
OMM3093_TLR22
OMM3094_TLR22
OMM3020
BX298853
OMM1527
CA058586
OMY10DIAS
OMM1375
OMM1540
OMM5214
Ssa64
OMM1433
OMM5088
OMM1399
OMM1025
OMM1223
OMM5194
OMM5018
OMM1619
OMM5307
Ssa103NVH
Ssa20_19NUIG
OMM5289
OMY105DUa
K cM
theta
0
0.1
0.2
0.3
0.4
0.5
0
10
20
30
40
50
60
70
80
90
100
110
120
130
140

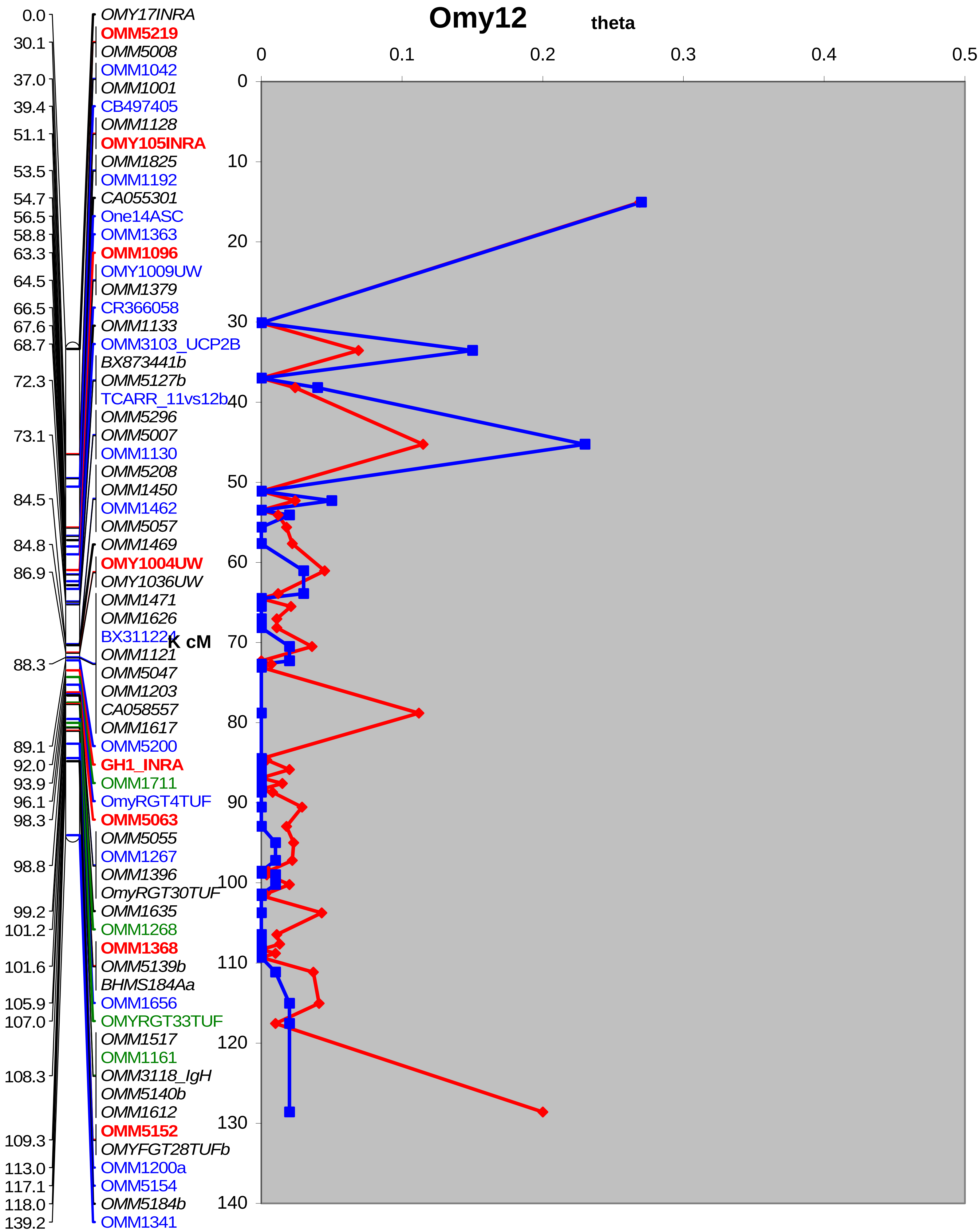
Omy12
theta
0
0.1
0.2
0.3
0.4
0.5
0
10
20
30
40
50
60
70
80
90
100
110
120
130
140
K cM
0.0
30.1
37.0
39.4
51.1
53.5
54.7
56.5
58.8
63.3
64.5
66.5
67.6
68.7
72.3
73.1
84.5
84.8
86.9
88.3
89.1
92.0
93.9
96.1
98.3
98.8
99.2
101.2
101.6
105.9
107.0
108.3
109.3
113.0
117.1
118.0
139.2
OMY17INRA
OMM5219
OMM5008
OMM1042
OMM1001
CB497405
OMM1128
OMY105INRA
OMM1825
OMM1192
CA055301
One14ASC
OMM1363
OMM1096
OMY1009UW
OMM1379
CR366058
OMM1133
OMM3103_UCP2B
BX873441b
OMM5127b
TCARR_11vs12b
OMM5296
OMM5007
OMM1130
OMM5208
OMM1450
OMM1462
OMM5057
OMM1469
OMY1004UW
OMY1036UW
OMM1471
OMM1626
BX311224
OMM1121
OMM5047
OMM1203
CA058557
OMM1617
OMM5200
GH1_INRA
OMM1711
OmyRGT4TUF
OMM5063
OMM5055
OMM1267
OMM1396
OmyRGT30TUF
OMM1635
OMM1268
OMM1368
OMM5139b
BHMS184Aa
OMM1656
OMYRGT33TUF
OMM1517
OMM1161
OMM3118_IgH
OMM5140b
OMM1612
OMM5152
OMYFGT28TUFb
OMM1200a
OMM5154
OMM5184b
OMM1341

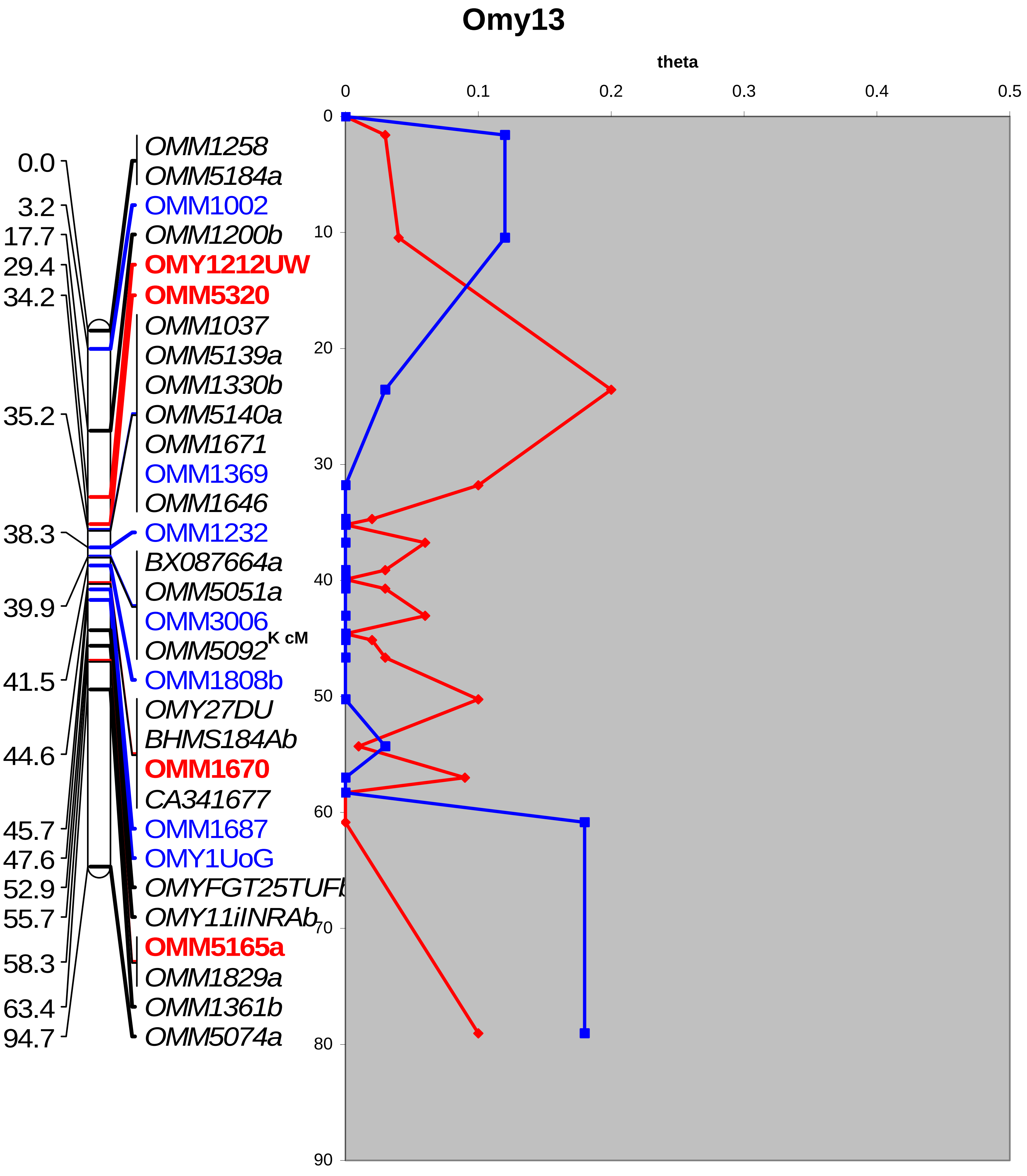

Omy13
theta
0
0.1
0.2
0.3
0.4
0.5
0
10
20
30
40
50
60
70
80
90
K cM
0.0
3.2
17.7
29.4
34.2
35.2
38.3
39.9
41.5
44.6
45.7
47.6
52.9
55.7
58.3
63.4
94.7
OMM1258
OMM5184a
OMM1002
OMM1200b
OMY1212UW
OMM5320
OMM1037
OMM5139a
OMM1330b
OMM5140a
OMM1671
OMM1369
OMM1646
OMM1232
BX087664a
OMM5051a
OMM3006
OMM5092
OMM1808b
OMY27DU
BHMS184Ab
OMM1670
CA341677
OMM1687
OMY1UoG
OMYFGT25TUFI
OMY11iINRAb
OMM5165a
OMM1829a
OMM1361b
OMM5074a

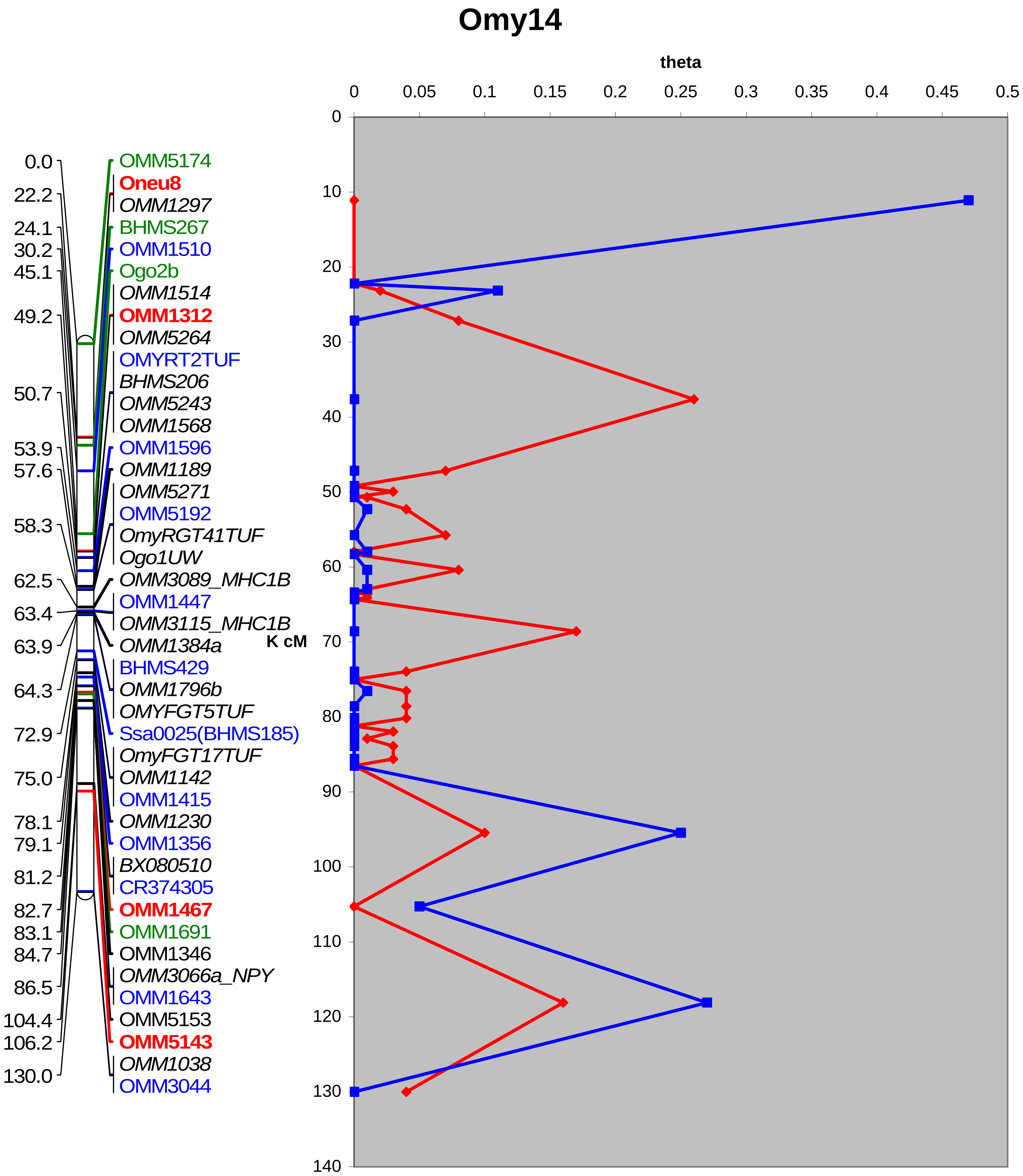
Omy14
theta
0
0.05
0.1
0.15
0.2
0.25
0.3
0.35
0.4
0.45
0.5
0
10
20
30
40
50
60
70
80
90
100
110
120
130
140
K cM
0.0
22.2
24.1
30.2
45.1
49.2
50.7
53.9
57.6
58.3
62.5
63.4
63.9
64.3
72.9
75.0
78.1
79.1
81.2
82.7
83.1
84.7
86.5
104.4
106.2
130.0
OMM5174
Oneu8
OMM1297
BHMS267
OMM1510
Ogo2b
OMM1514
OMM1312
OMM5264
OMYRT2TUF
BHMS206
OMM5243
OMM1568
OMM1596
OMM1189
OMM5271
OMM5192
OmyRGT41TUF
Ogo1UW
OMM3089_MHC1B
OMM1447
OMM3115_MHC1B
OMM1384a
BHMS429
OMM1796b
OMYFGT5TUF
Ssa0025(BHMS185)
OmyFGT17TUF
OMM1142
OMM1415
OMM1230
OMM1356
BX080510
CR374305
OMM1467
OMM1691
OMM1346
OMM3066a_NPY
OMM1643
OMM5153
OMM5143
OMM1038
OMM3044

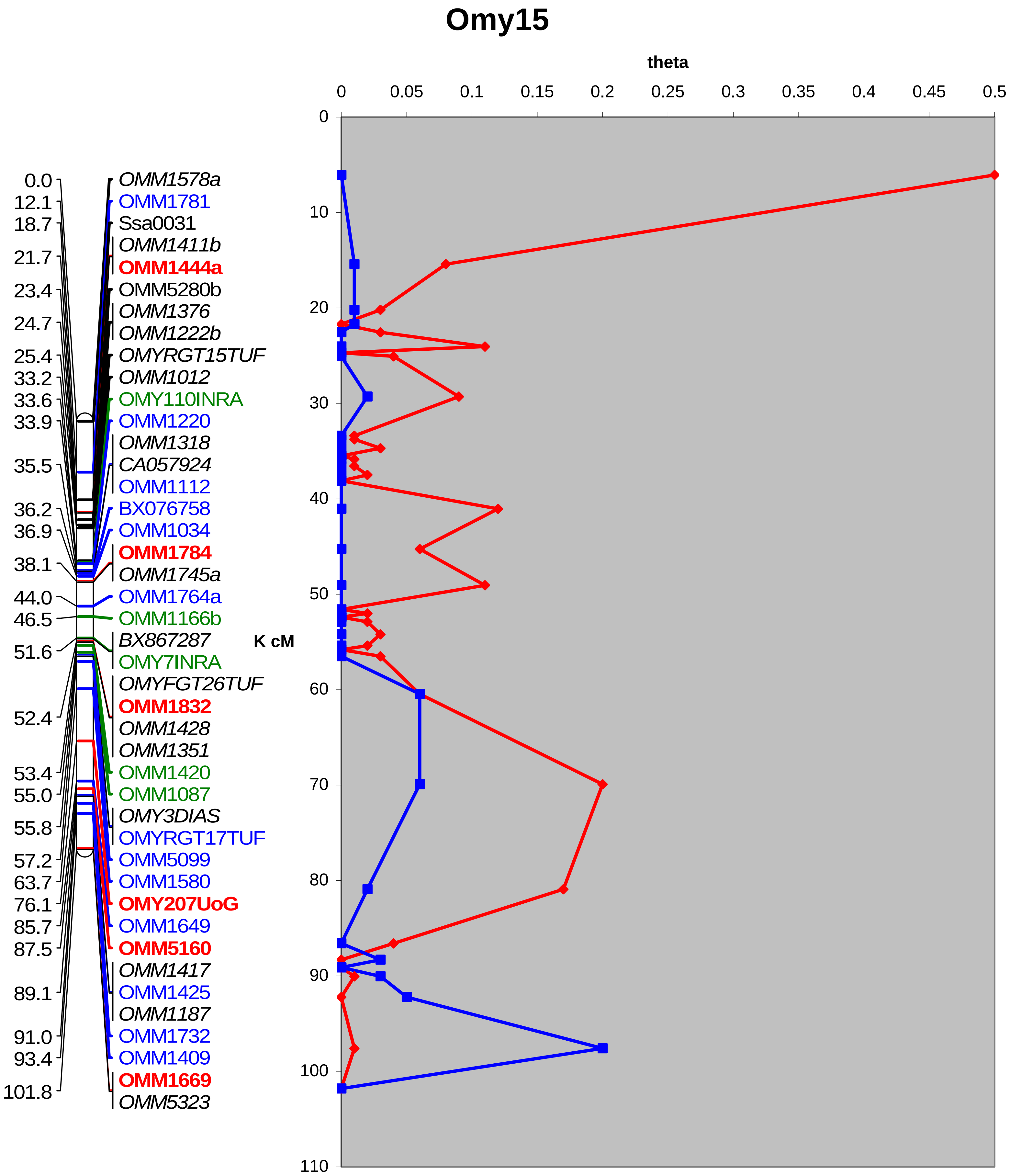
Omy15
theta
0
0.05
0.1
0.15
0.2
0.25
0.3
0.35
0.4
0.45
0.5
0
10
20
30
40
50
60
70
80
90
100
110
K cM
0.0 OMM1578a
12.1 OMM1781
18.7 Ssa0031
21.7 OMM1411b
OMM1444a
23.4 OMM5280b
24.7 OMM1376
OMM1222b
25.4 OMYRGT15TUF
33.2 OMM1012
33.6 OMY110INRA
33.9 OMM1220
35.5 OMM1318
CA057924
OMM1112
36.2 BX076758
36.9 OMM1034
38.1 OMM1784
OMM1745a
44.0 OMM1764a
46.5 OMM1166b
51.6 BX867287
OMY7INRA
52.4 OMYFGT26TUF
OMM1832
OMM1428
OMM1351
53.4 OMM1420
55.0 OMM1087
55.8 OMY3DIAS
OMYRGT17TUF
57.2 OMM5099
63.7 OMM1580
76.1 OMY207UoG
85.7 OMM1649
87.5 OMM5160
89.1 OMM1417
OMM1425
OMM1187
91.0 OMM1732
93.4 OMM1409
101.8 OMM1669
OMM5323

Omy16

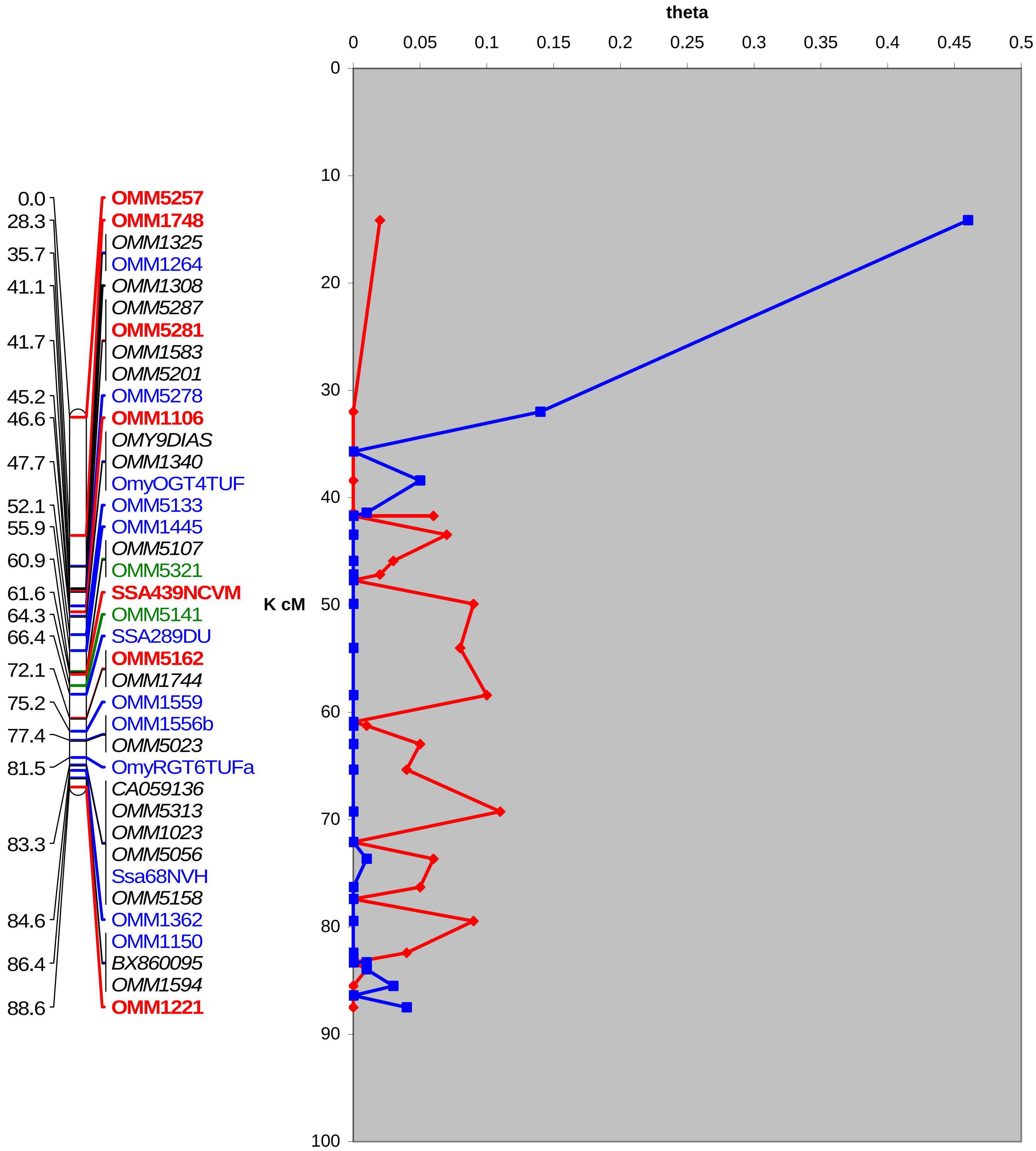

theta
K cM
0.0
28.3
35.7
41.1
41.7
45.2
46.6
47.7
52.1
55.9
60.9
61.6
64.3
66.4
72.1
75.2
77.4
81.5
83.3
84.6
86.4
88.6
OMM5257
OMM1748
OMM1325
OMM1264
OMM1308
OMM5287
OMM5281
OMM1583
OMM5201
OMM5278
OMM1106
OMY9DIAS
OMM1340
OmyOGT4TUF
OMM5133
OMM1445
OMM5107
OMM5321
SSA439NCVM
OMM5141
SSA289DU
OMM5162
OMM1744
OMM1559
OMM1556b
OMM5023
OmyRGT6TUFa
CA059136
OMM5313
OMM1023
OMM5056
Ssa68NVH
OMM5158
OMM1362
OMM1150
BX860095
OMM1594
OMM1221

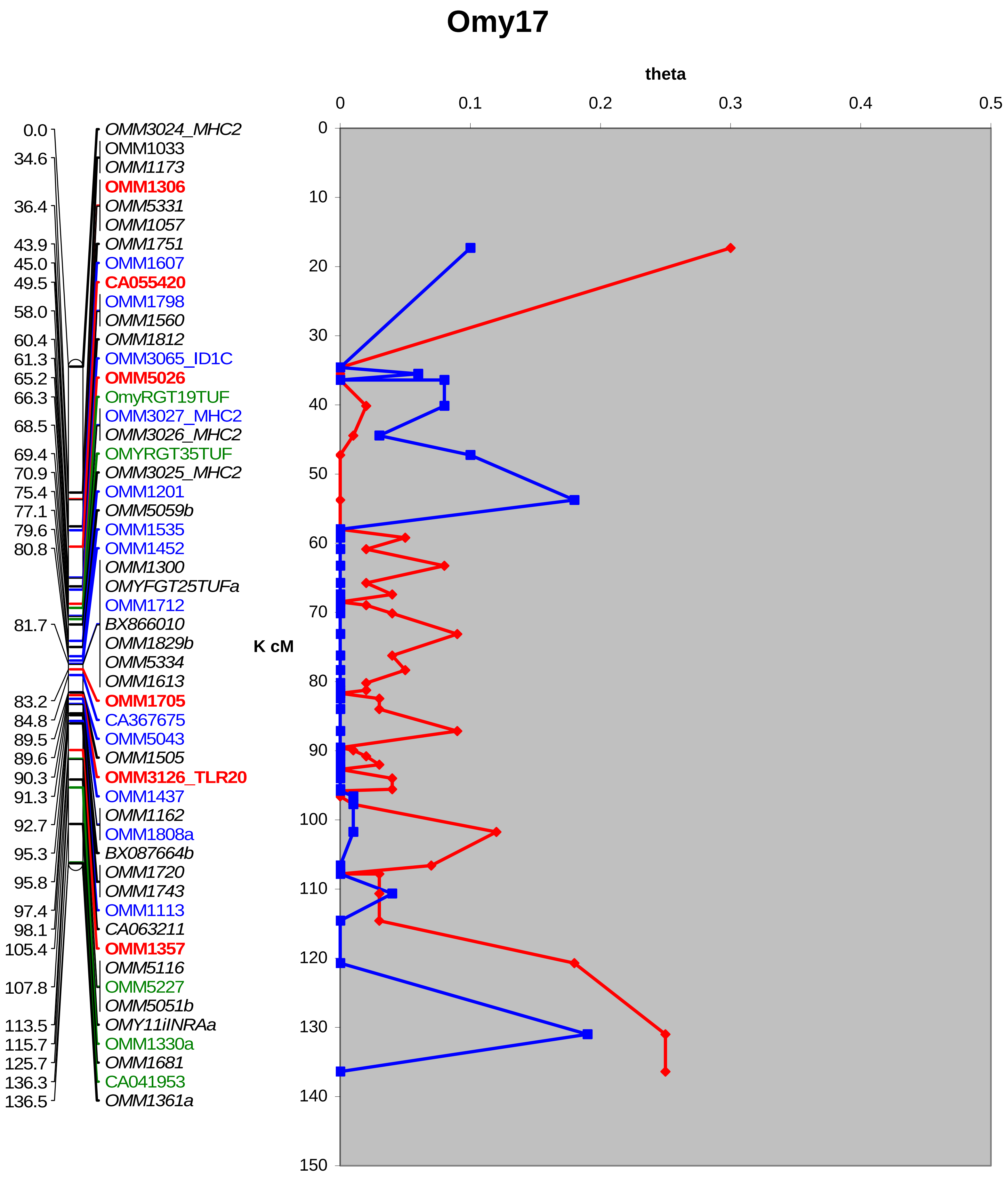
Omy17
theta
0
0.1
0.2
0.3
0.4
0.5
K cM
0.0
34.6
36.4
43.9
45.0
49.5
58.0
60.4
61.3
65.2
66.3
68.5
69.4
70.9
75.4
77.1
79.6
80.8
81.7
83.2
84.8
89.5
89.6
90.3
91.3
92.7
95.3
95.8
97.4
98.1
105.4
107.8
113.5
115.7
125.7
136.3
136.5
OMM3024_MHC2
OMM1033
OMM1173
OMM1306
OMM5331
OMM1057
OMM1751
OMM1607
CA055420
OMM1798
OMM1560
OMM1812
OMM3065_ID1C
OMM5026
OmyRGT19TUF
OMM3027_MHC2
OMM3026_MHC2
OMYRGT35TUF
OMM3025_MHC2
OMM1201
OMM5059b
OMM1535
OMM1452
OMM1300
OMYFGT25TUFa
OMM1712
BX866010
OMM1829b
OMM5334
OMM1613
OMM1705
CA367675
OMM5043
OMM1505
OMM3126_TLR20
OMM1437
OMM1162
OMM1808a
BX087664b
OMM1720
OMM1743
OMM1113
CA063211
OMM1357
OMM5116
OMM5227
OMM5051b
OMY11iINRAa
OMM1330a
OMM1681
CA041953
OMM1361a
0
10
20
30
40
50
60
70
80
90
100
110
120
130
140
150

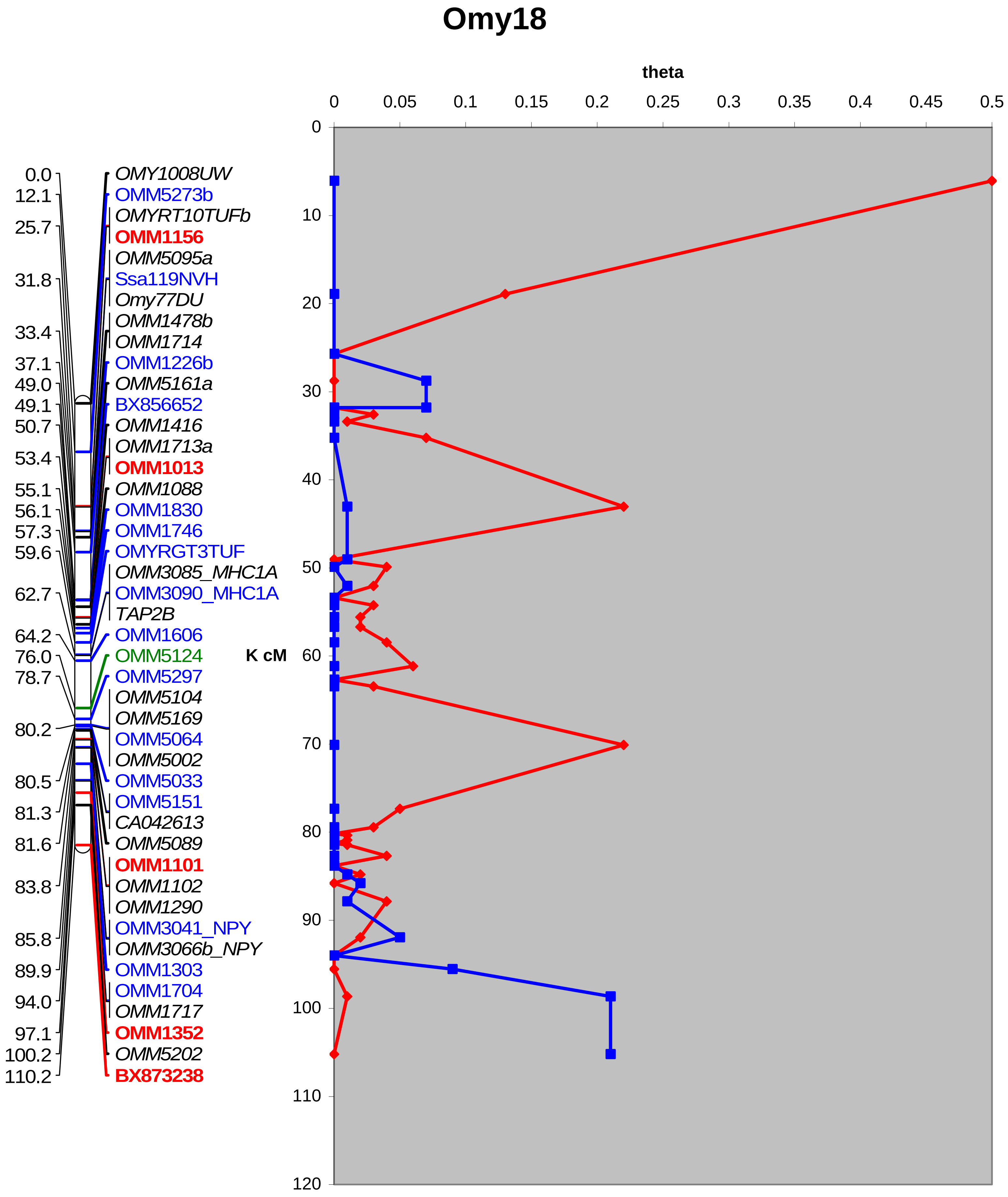

Omy18
theta
0
0.05
0.1
0.15
0.2
0.25
0.3
0.35
0.4
0.45
0.5
K cM
0.0 OMY1008UW
12.1 OMM5273b
OMYRT10TUFb
25.7 OMM1156
OMM5095a
31.8 Ssa119NVH
Omy77DU
OMM1478b
33.4 OMM1714
37.1 OMM1226b
49.0 OMM5161a
49.1 BX856652
50.7 OMM1416
OMM1713a
53.4 OMM1013
55.1 OMM1088
56.1 OMM1830
57.3 OMM1746
59.6 OMYRGT3TUF
OMM3085_MHC1A
62.7 OMM3090_MHC1A
TAP2B
64.2 OMM1606
76.0 OMM5124
78.7 OMM5297
OMM5104
OMM5169
80.2 OMM5064
OMM5002
80.5 OMM5033
OMM5151
81.3 CA042613
81.6 OMM5089
OMM1101
83.8 OMM1102
OMM1290
OMM3041_NPY
85.8 OMM3066b_NPY
89.9 OMM1303
OMM1704
94.0 OMM1717
97.1 OMM1352
100.2 OMM5202
110.2 BX873238
0
10
20
30
40
50
60
70
80
90
100
110
120

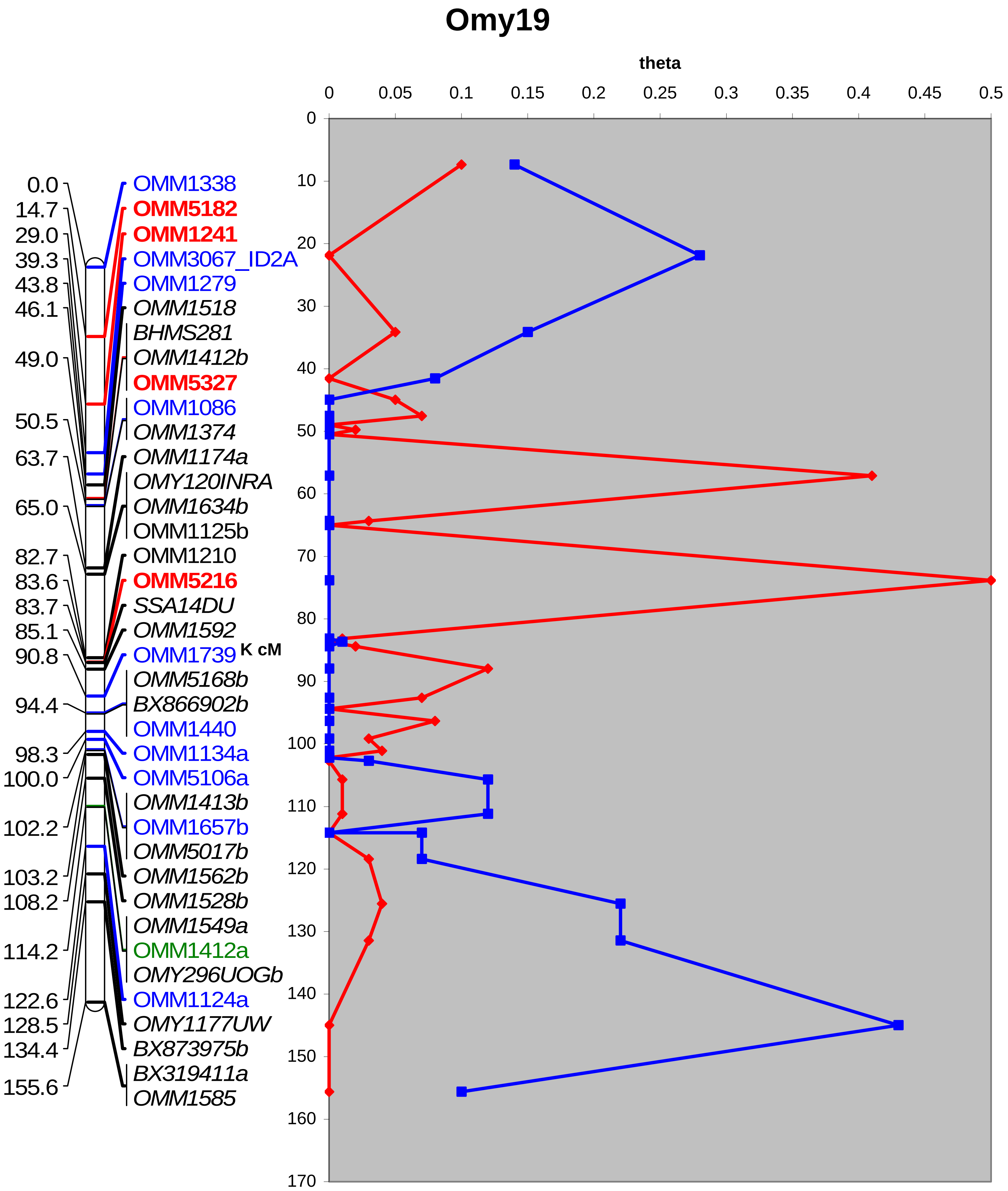
Omy19
theta
0
0.05
0.1
0.15
0.2
0.25
0.3
0.35
0.4
0.45
0.5
0
10
20
30
40
50
60
70
80
90
100
110
120
130
140
150
160
170
K cM
0.0
14.7
29.0
39.3
43.8
46.1
49.0
50.5
63.7
65.0
82.7
83.6
83.7
85.1
90.8
94.4
98.3
100.0
102.2
103.2
108.2
114.2
122.6
128.5
134.4
155.6
OMM1338
OMM5182
OMM1241
OMM3067_ID2A
OMM1279
OMM1518
BHMS281
OMM1412b
OMM5327
OMM1086
OMM1374
OMM1174a
OMY120INRA
OMM1634b
OMM1125b
OMM1210
OMM5216
SSA14DU
OMM1592
OMM1739
OMM5168b
BX866902b
OMM1440
OMM1134a
OMM5106a
OMM1413b
OMM1657b
OMM5017b
OMM1562b
OMM1528b
OMM1549a
OMM1412a
OMY296UOGb
OMM1124a
OMY1177UW
BX873975b
BX319411a
OMM1585

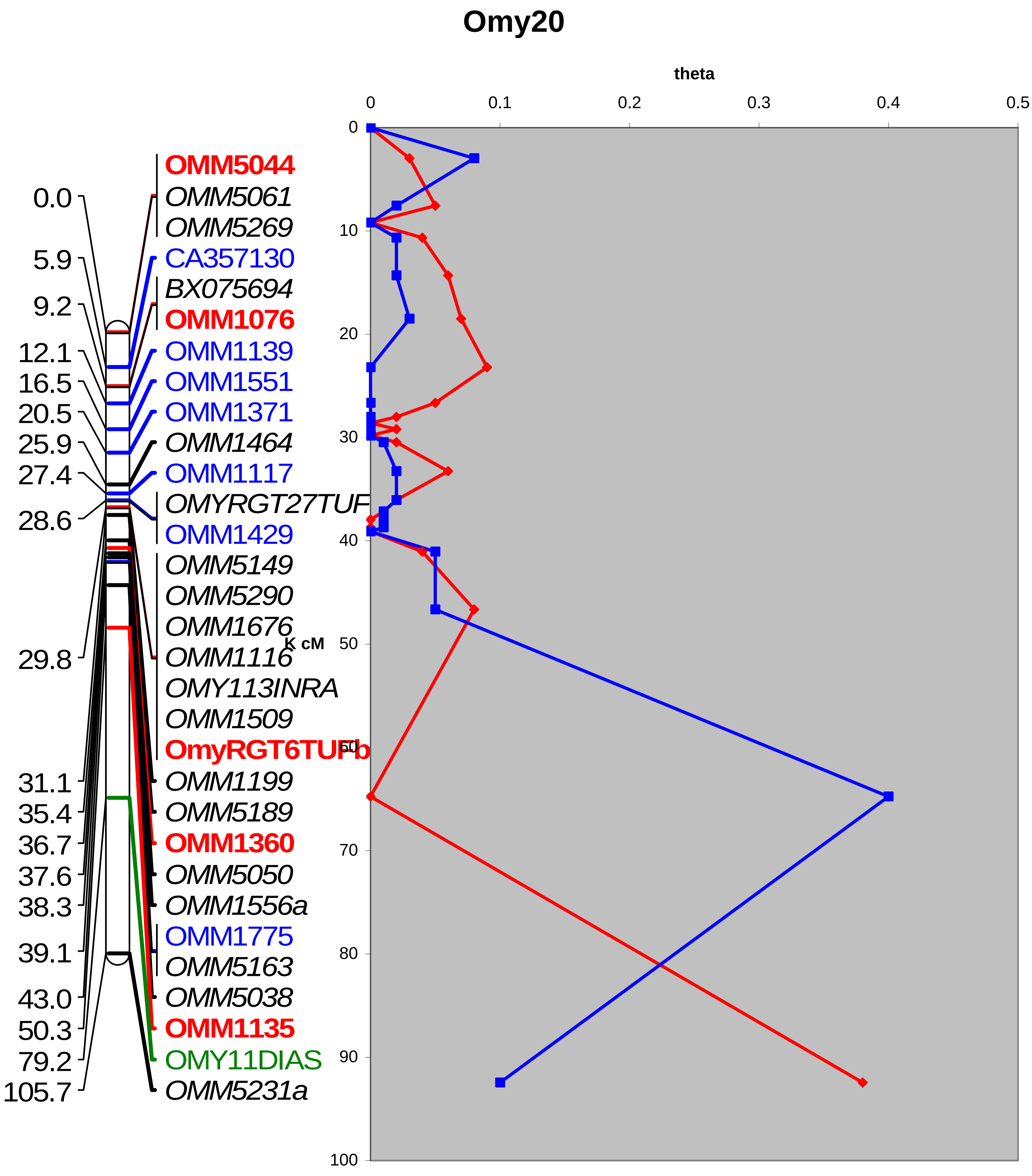

Omy20
theta
0
0.1
0.2
0.3
0.4
0.5
0
10
20
30
40
50
60
70
80
90
100
K cM
0.0
5.9
9.2
12.1
16.5
20.5
25.9
27.4
28.6
29.8
31.1
35.4
36.7
37.6
38.3
39.1
43.0
50.3
79.2
105.7
OMM5044
OMM5061
OMM5269
CA357130
BX075694
OMM1076
OMM1139
OMM1551
OMM1371
OMM1464
OMM1117
OMYRGT27TUF
OMM1429
OMM5149
OMM5290
OMM1676
OMM1116
OMY113INRA
OMM1509
OmyRGT6TUFb
OMM1199
OMM5189
OMM1360
OMM5050
OMM1556a
OMM1775
OMM5163
OMM5038
OMM1135
OMY11DIAS
OMM5231a

Omy21

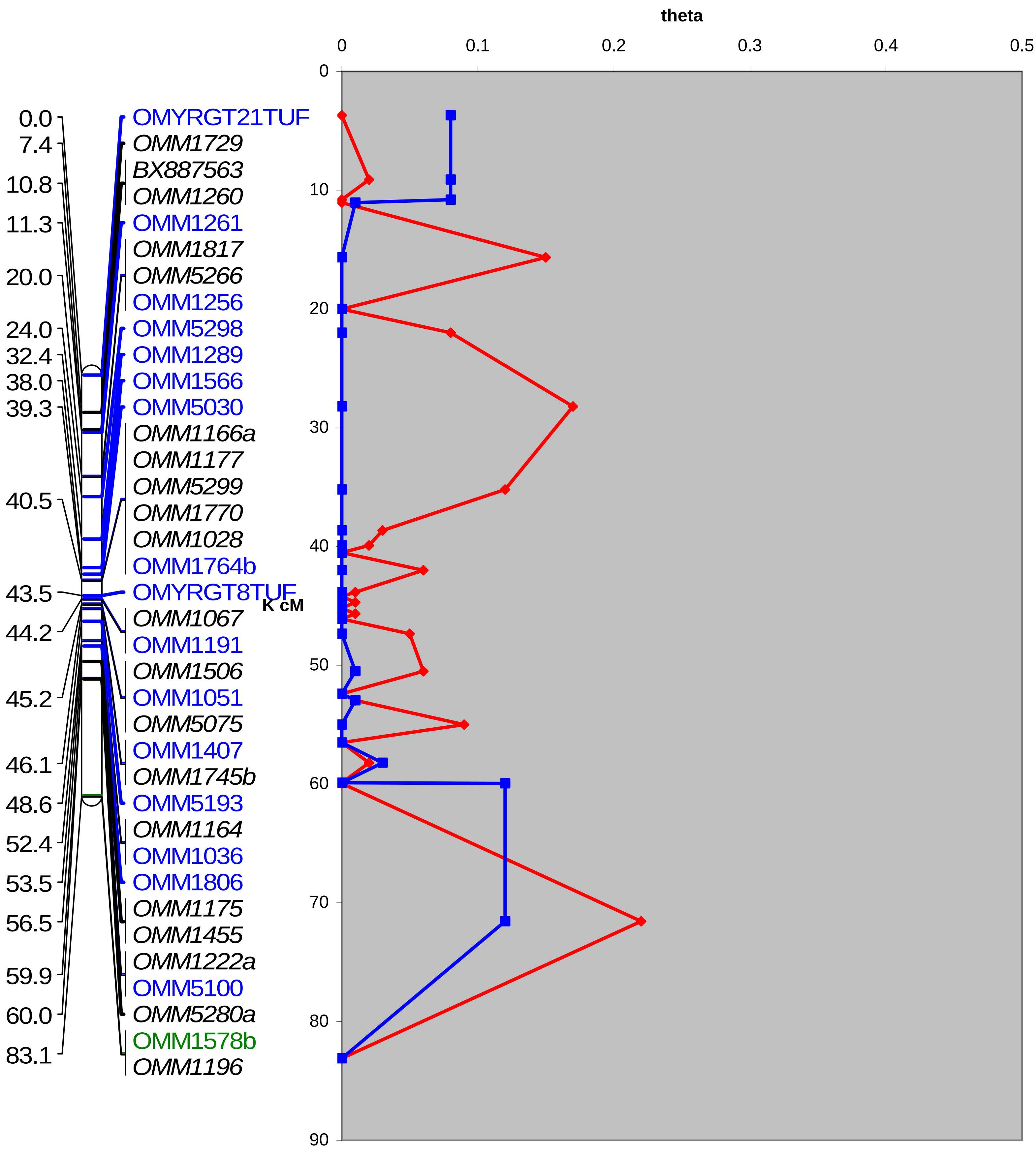
theta
0
0.1
0.2
0.3
0.4
0.5
0
10
20
30
40
50
60
70
80
90
K cM
0.0
7.4
10.8
11.3
20.0
24.0
32.4
38.0
39.3
40.5
43.5
44.2
45.2
46.1
48.6
52.4
53.5
56.5
59.9
60.0
83.1
OMYRGT21TUF
OMM1729
BX887563
OMM1260
OMM1261
OMM1817
OMM5266
OMM1256
OMM5298
OMM1289
OMM1566
OMM5030
OMM1166a
OMM1177
OMM5299
OMM1770
OMM1028
OMM1764b
OMYRGT8TUF
OMM1067
OMM1191
OMM1506
OMM1051
OMM5075
OMM1407
OMM1745b
OMM5193
OMM1164
OMM1036
OMM1806
OMM1175
OMM1455
OMM1222a
OMM5100
OMM5280a
OMM1578b
OMM1196

Omy22

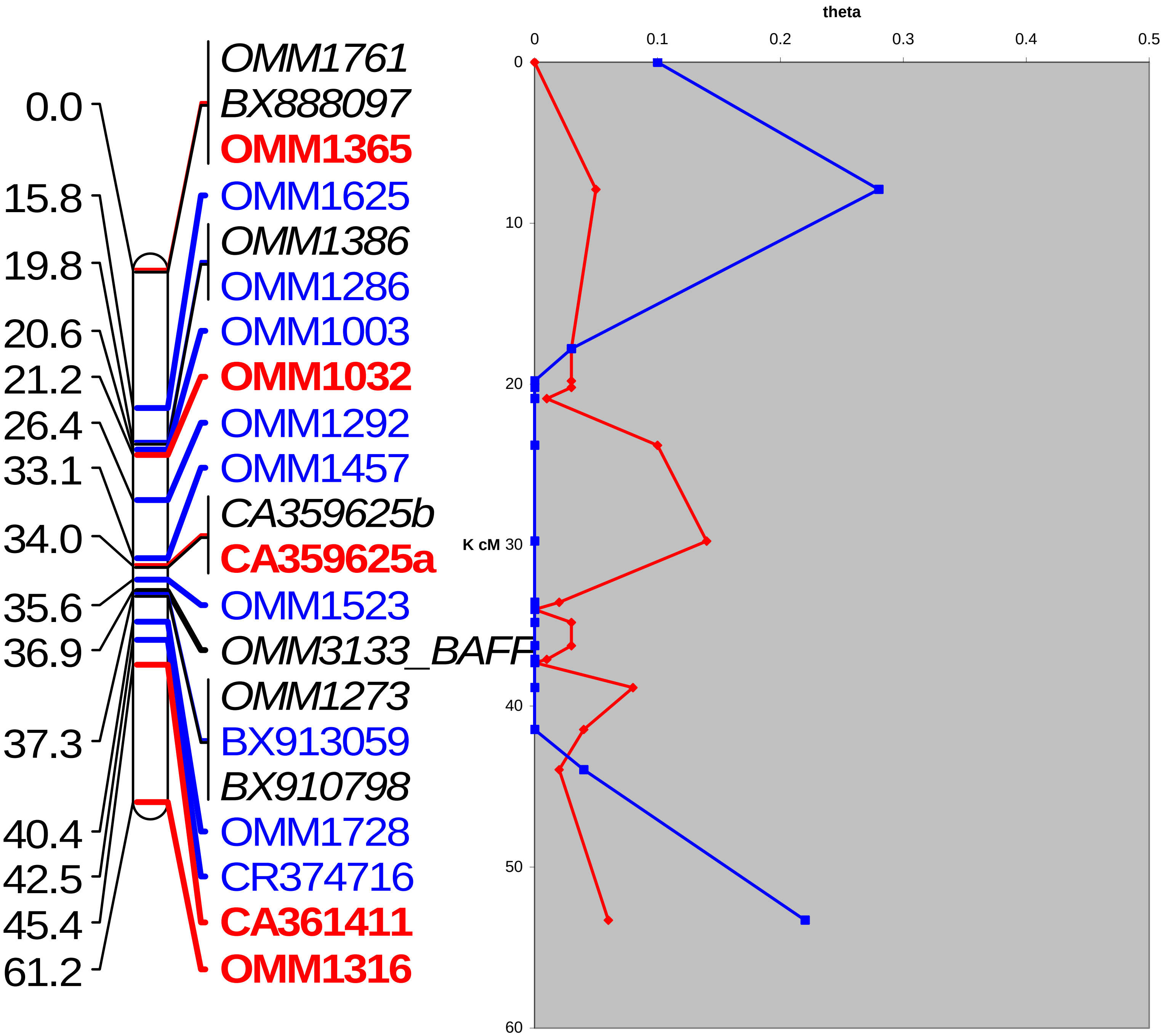
0.0
15.8
19.8
20.6
21.2
26.4
33.1
34.0
35.6
36.9
37.3
40.4
42.5
45.4
61.2
OMM1761
BX888097
OMM1365
OMM1625
OMM1386
OMM1286
OMM1003
OMM1032
OMM1292
OMM1457
CA359625b
CA359625a
OMM1523
OMM3133_BAFF
OMM1273
BX913059
BX910798
OMM1728
CR374716
CA361411
OMM1316
theta
0
0.1
0.2
0.3
0.4
0.5
K cM
0
10
20
30
40
50
60

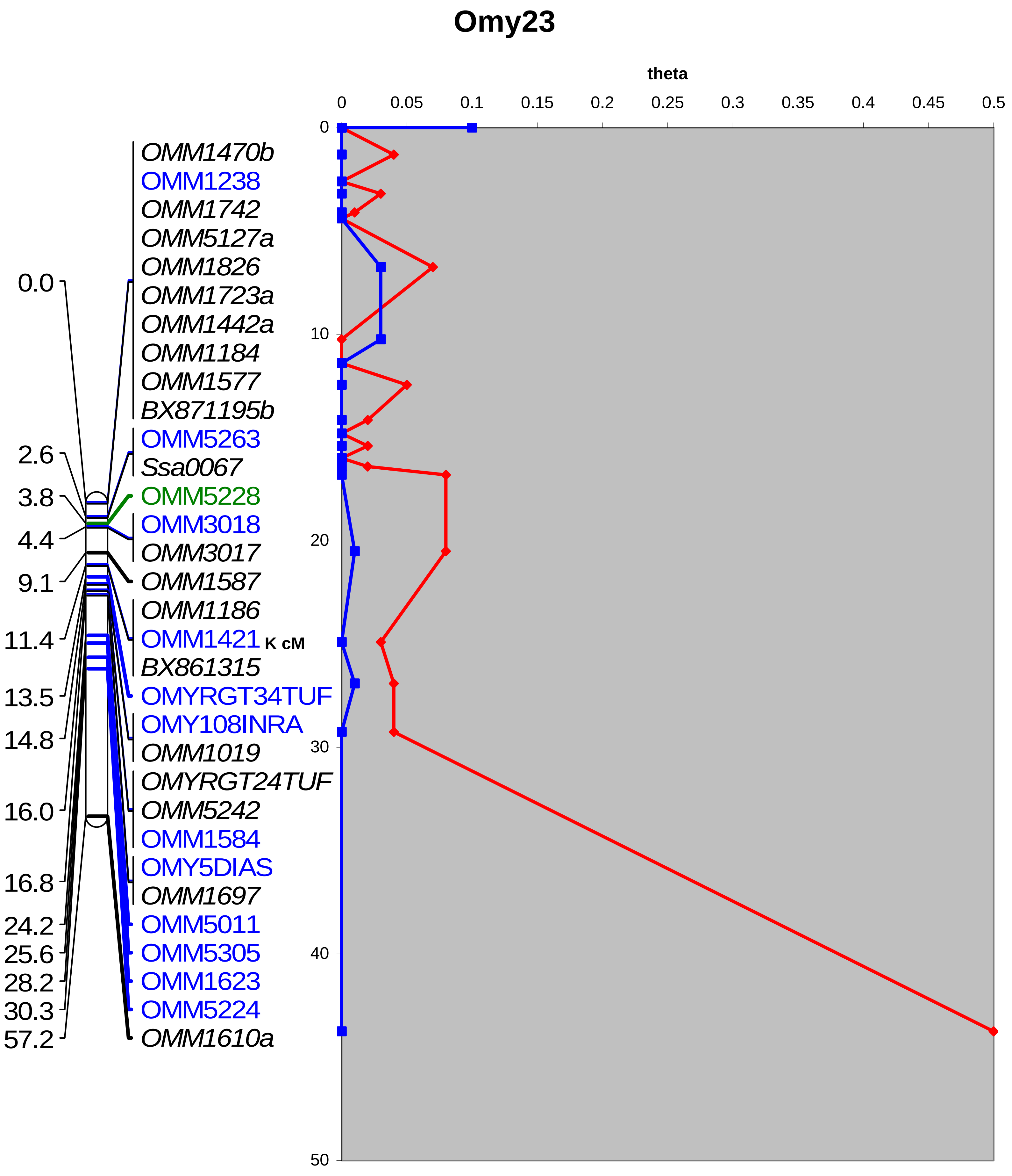

Omy23
theta
0
0.05
0.1
0.15
0.2
0.25
0.3
0.35
0.4
0.45
0.5
0
10
20
30
40
50
0.0
2.6
3.8
4.4
9.1
11.4
13.5
14.8
16.0
16.8
24.2
25.6
28.2
30.3
57.2
OMM1470b
OMM1238
OMM1742
OMM5127a
OMM1826
OMM1723a
OMM1442a
OMM1184
OMM1577
BX871195b
OMM5263
Ssa0067
OMM5228
OMM3018
OMM3017
OMM1587
OMM1186
OMM1421
K cM
BX861315
OMYRGT34TUF
OMY108INRA
OMM1019
OMYRGT24TUF
OMM5242
OMM1584
OMY5DIAS
OMM1697
OMM5011
OMM5305
OMM1623
OMM5224
OMM1610a

Omy24

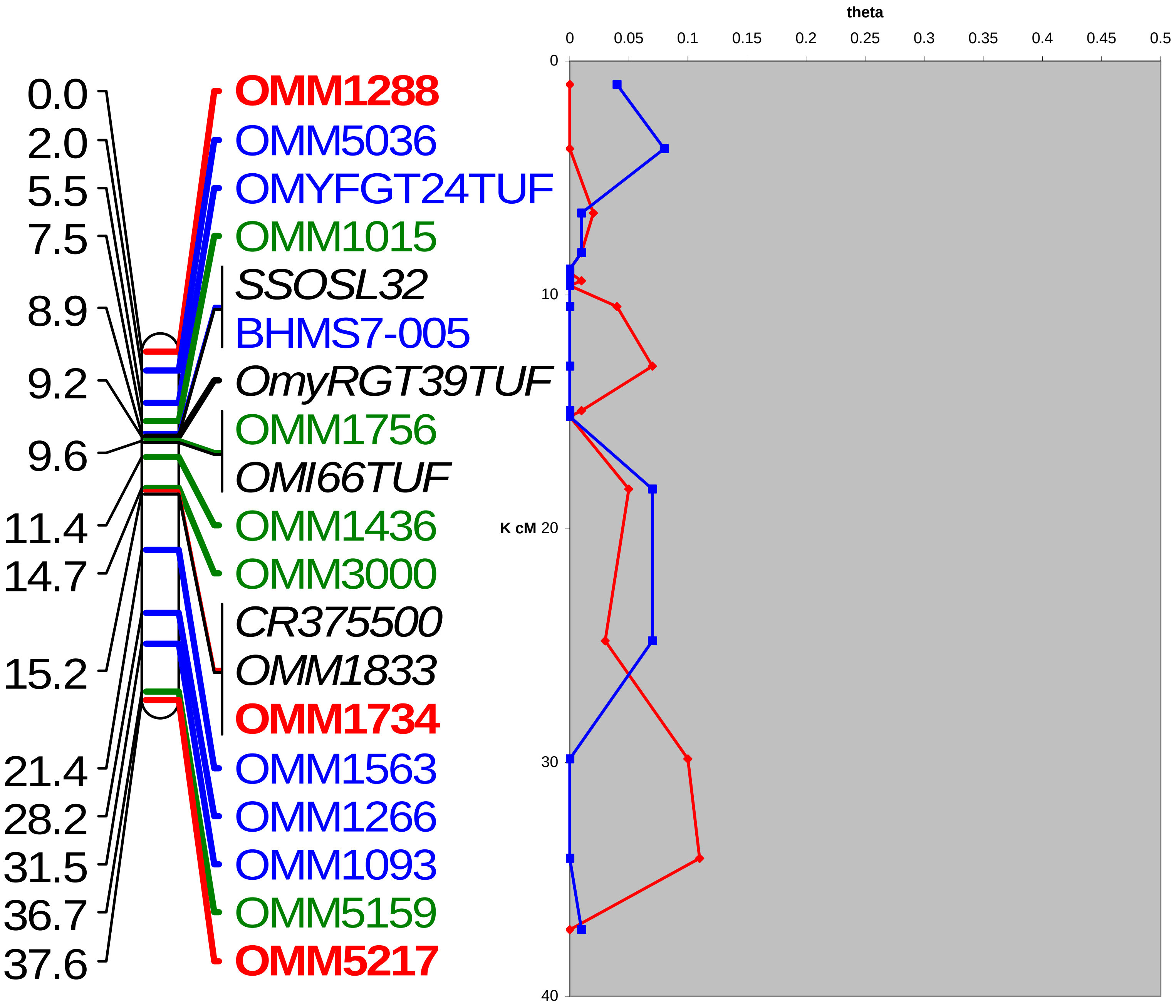

0.0
2.0
5.5
7.5
8.9
9.2
9.6
11.4
14.7
15.2
21.4
28.2
31.5
36.7
37.6
OMM1288
OMM5036
OMYFGT24TUF
OMM1015
SSOSL32
BHMS7-005
OmyRGT39TUF
OMM1756
OMI66TUF
OMM1436
OMM3000
CR375500
OMM1833
OMM1734
OMM1563
OMM1266
OMM1093
OMM5159
OMM5217
theta
0
0.05
0.1
0.15
0.2
0.25
0.3
0.35
0.4
0.45
0.5
0
10
K cM 20
30
40

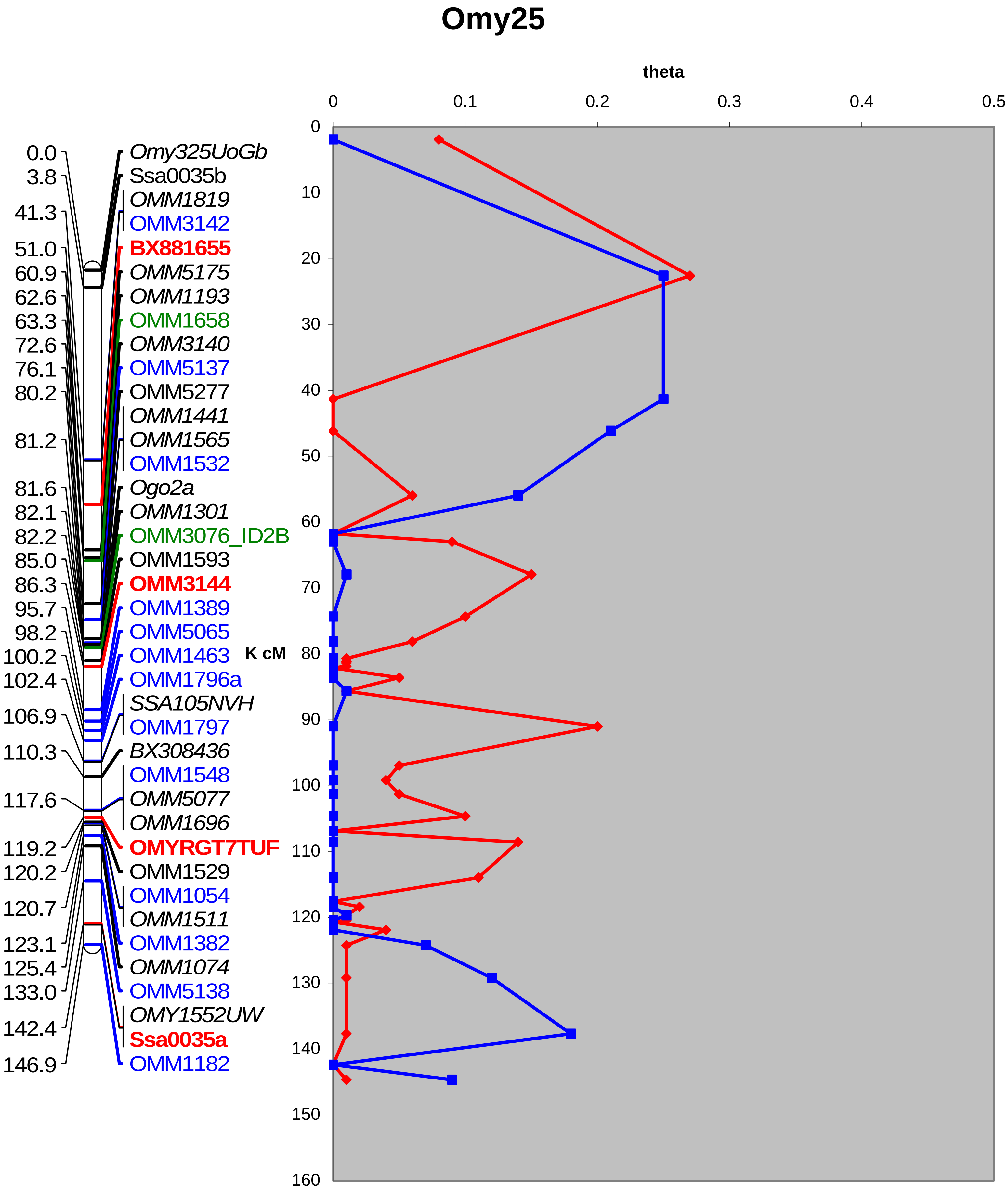
Omy25
theta
0
0.1
0.2
0.3
0.4
0.5
0
10
20
30
40
50
60
70
80
90
100
110
120
130
140
150
160
K cM
0.0 Omy325UoGb
3.8 Ssa0035b
OMM1819
41.3 OMM3142
51.0 BX881655
60.9 OMM5175
62.6 OMM1193
63.3 OMM1658
72.6 OMM3140
76.1 OMM5137
80.2 OMM5277
OMM1441
81.2 OMM1565
OMM1532
81.6 Ogo2a
82.1 OMM1301
82.2 OMM3076_ID2B
85.0 OMM1593
86.3 OMM3144
95.7 OMM1389
98.2 OMM5065
100.2 OMM1463
102.4 OMM1796a
SSA105NVH
106.9 OMM1797
110.3 BX308436
OMM1548
117.6 OMM5077
OMM1696
119.2 OMYRGT7TUF
120.2 OMM1529
OMM1054
120.7 OMM1511
123.1 OMM1382
125.4 OMM1074
133.0 OMM5138
OMY1552UW
142.4 Ssa0035a
146.9 OMM1182

Omy26

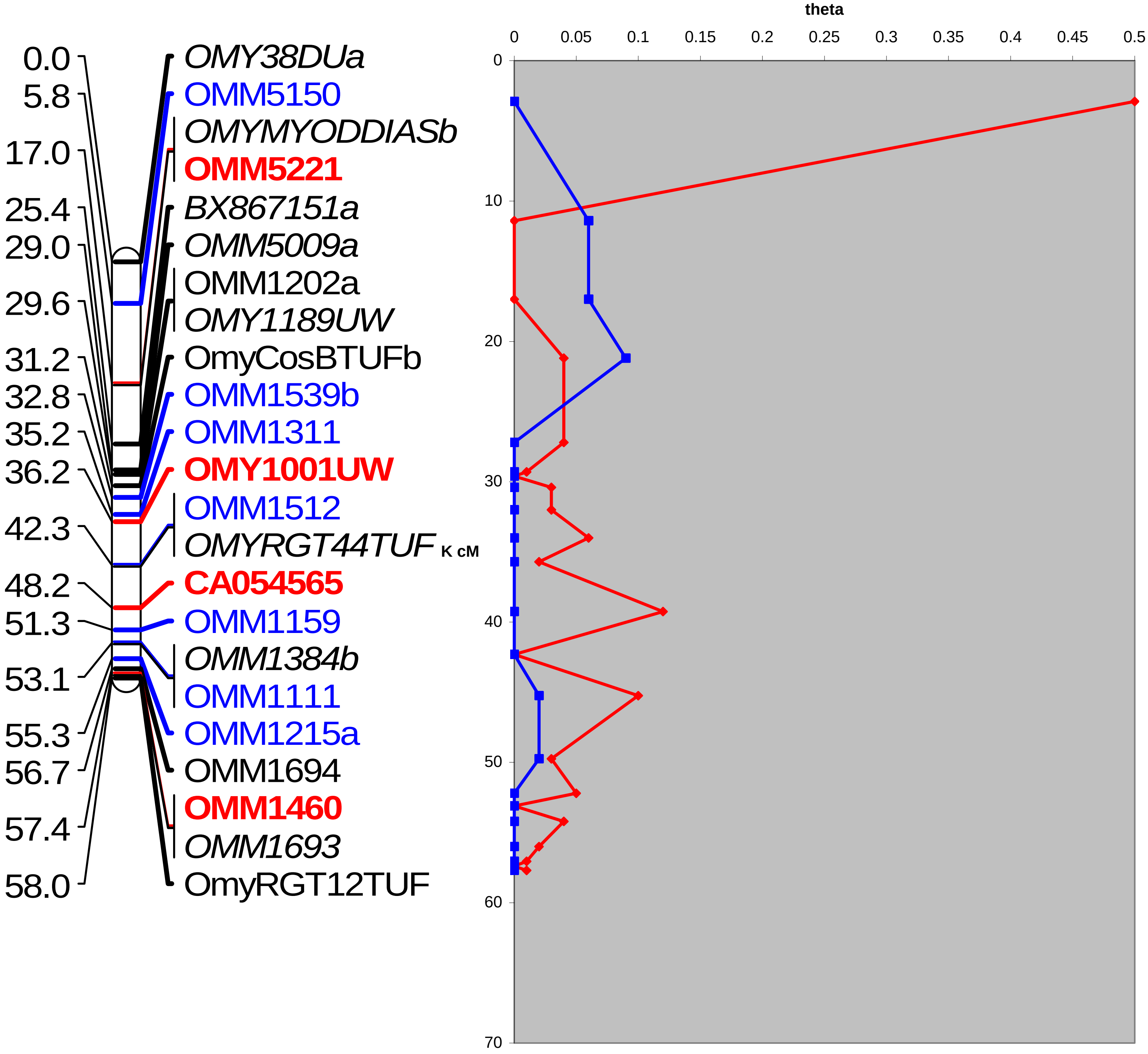

0.0
5.8
17.0
25.4
29.0
29.6
31.2
32.8
35.2
36.2
42.3
48.2
51.3
53.1
55.3
56.7
57.4
58.0
OMY38DUa
OMM5150
OMYMYODDIASb
OMM5221
BX867151a
OMM5009a
OMM1202a
OMY1189UW
OmyCosBTUFb
OMM1539b
OMM1311
OMY1001UW
OMM1512
OMYRGT44TUF
K cM
CA054565
OMM1159
OMM1384b
OMM1111
OMM1215a
OMM1694
OMM1460
OMM1693
OmyRGT12TUF
theta
0
0.05
0.1
0.15
0.2
0.25
0.3
0.35
0.4
0.45
0.5
0
10
20
30
40
50
60
70

Omy27

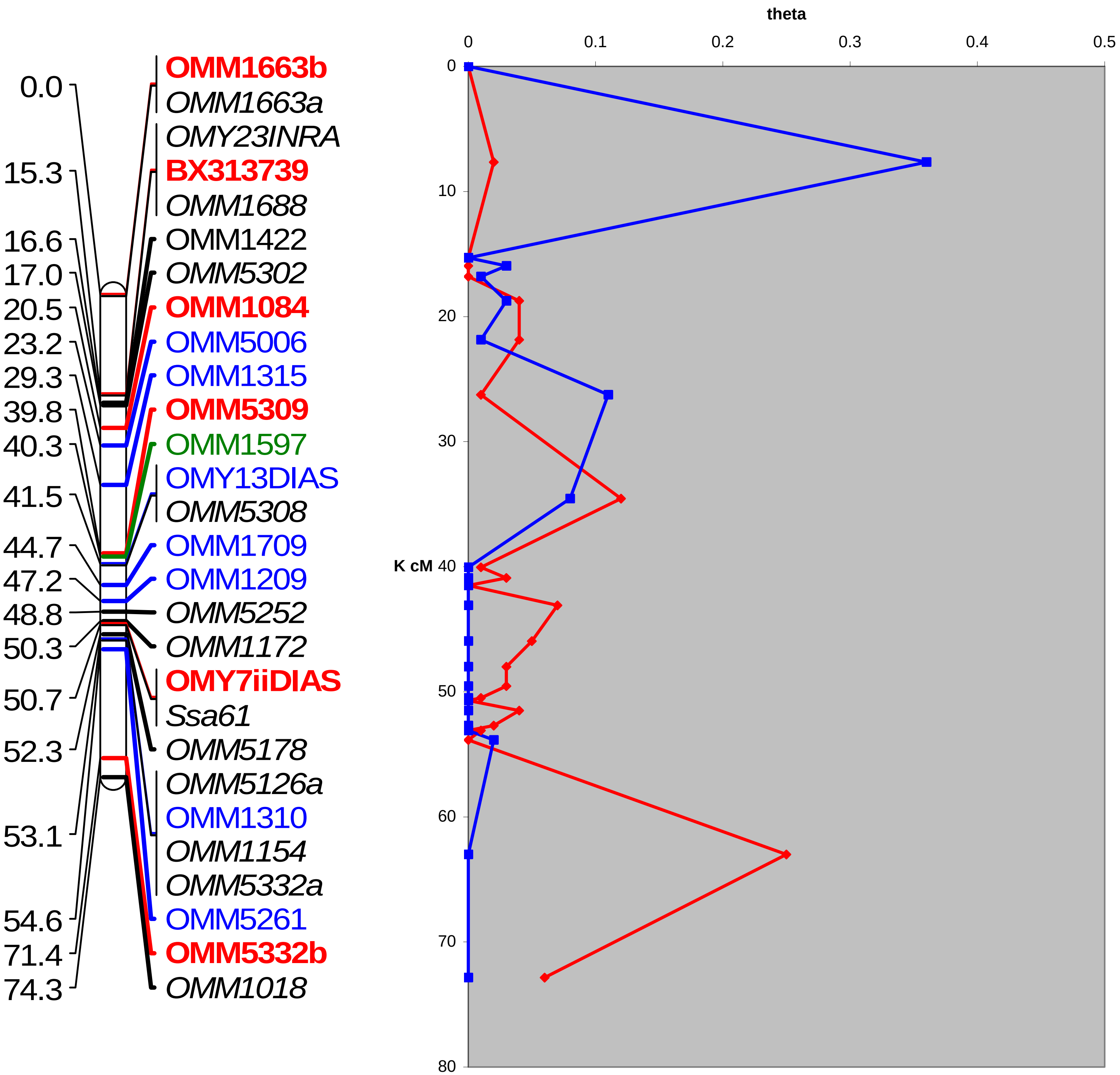

0.0
15.3
16.6
17.0
20.5
23.2
29.3
39.8
40.3
41.5
44.7
47.2
48.8
50.3
50.7
52.3
53.1
54.6
71.4
74.3
OMM1663b
OMM1663a
OMY23INRA
BX313739
OMM1688
OMM1422
OMM5302
OMM1084
OMM5006
OMM1315
OMM5309
OMM1597
OMY13DIAS
OMM5308
OMM1709
OMM1209
OMM5252
OMM1172
OMY7iiDIAS
Ssa61
OMM5178
OMM5126a
OMM1310
OMM1154
OMM5332a
OMM5261
OMM5332b
OMM1018
theta
0
0.1
0.2
0.3
0.4
0.5
K cM
0
10
20
30
40
50
60
70
80

Omy28

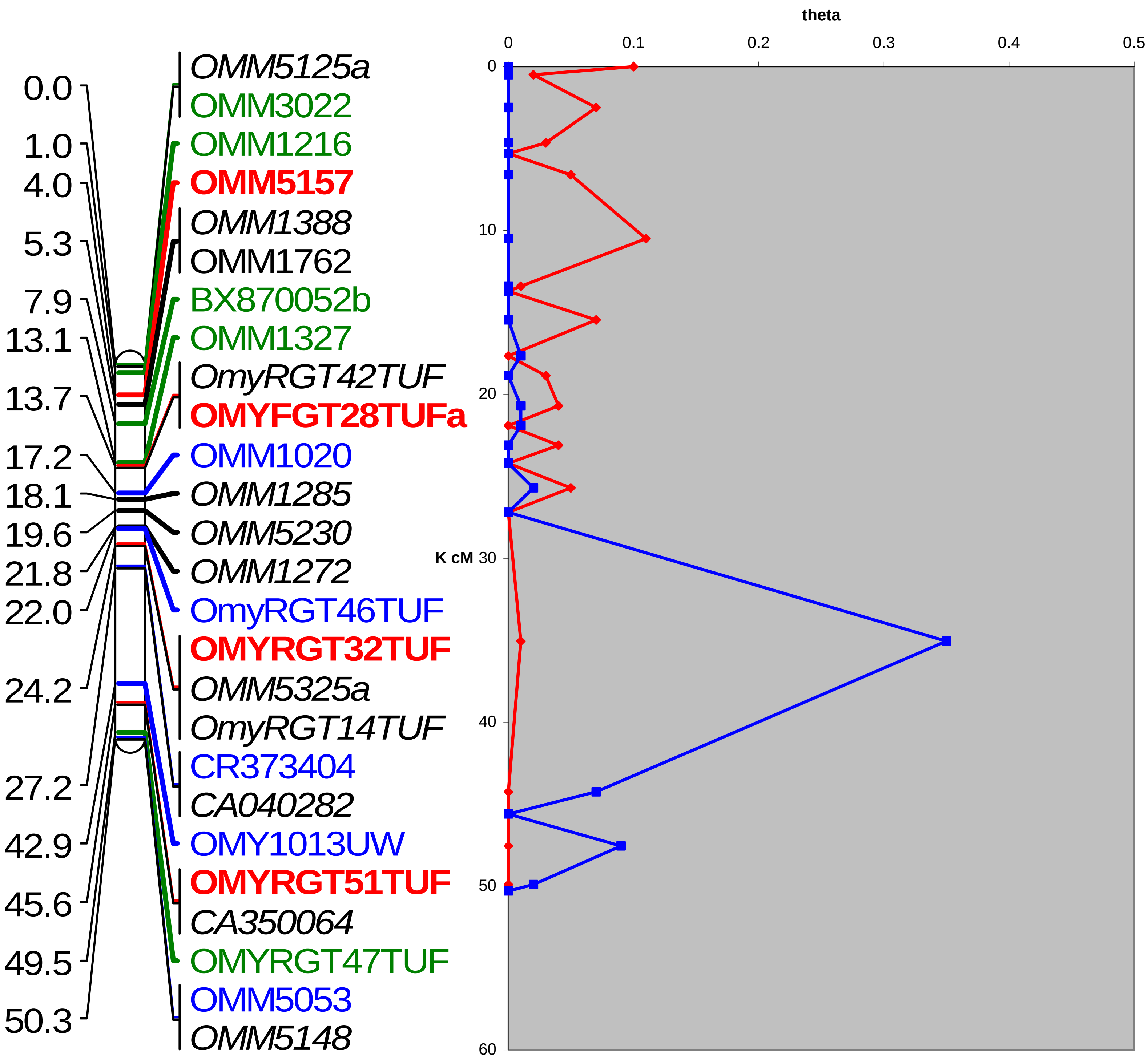

0.0
1.0
4.0
5.3
7.9
13.1
13.7
17.2
18.1
19.6
21.8
22.0
24.2
27.2
42.9
45.6
49.5
50.3
OMM5125a
OMM3022
OMM1216
OMM5157
OMM1388
OMM1762
BX870052b
OMM1327
OmyRGT42TUF
OMYFGT28TUFa
OMM1020
OMM1285
OMM5230
OMM1272
OmyRGT46TUF
OMYRGT32TUF
OMM5325a
OmyRGT14TUF
CR373404
CA040282
OMY1013UW
OMYRGT51TUF
CA350064
OMYRGT47TUF
OMM5053
OMM5148
theta
0
0.1
0.2
0.3
0.4
0.5
K cM
0
10
20
30
40
50
60

OmySex

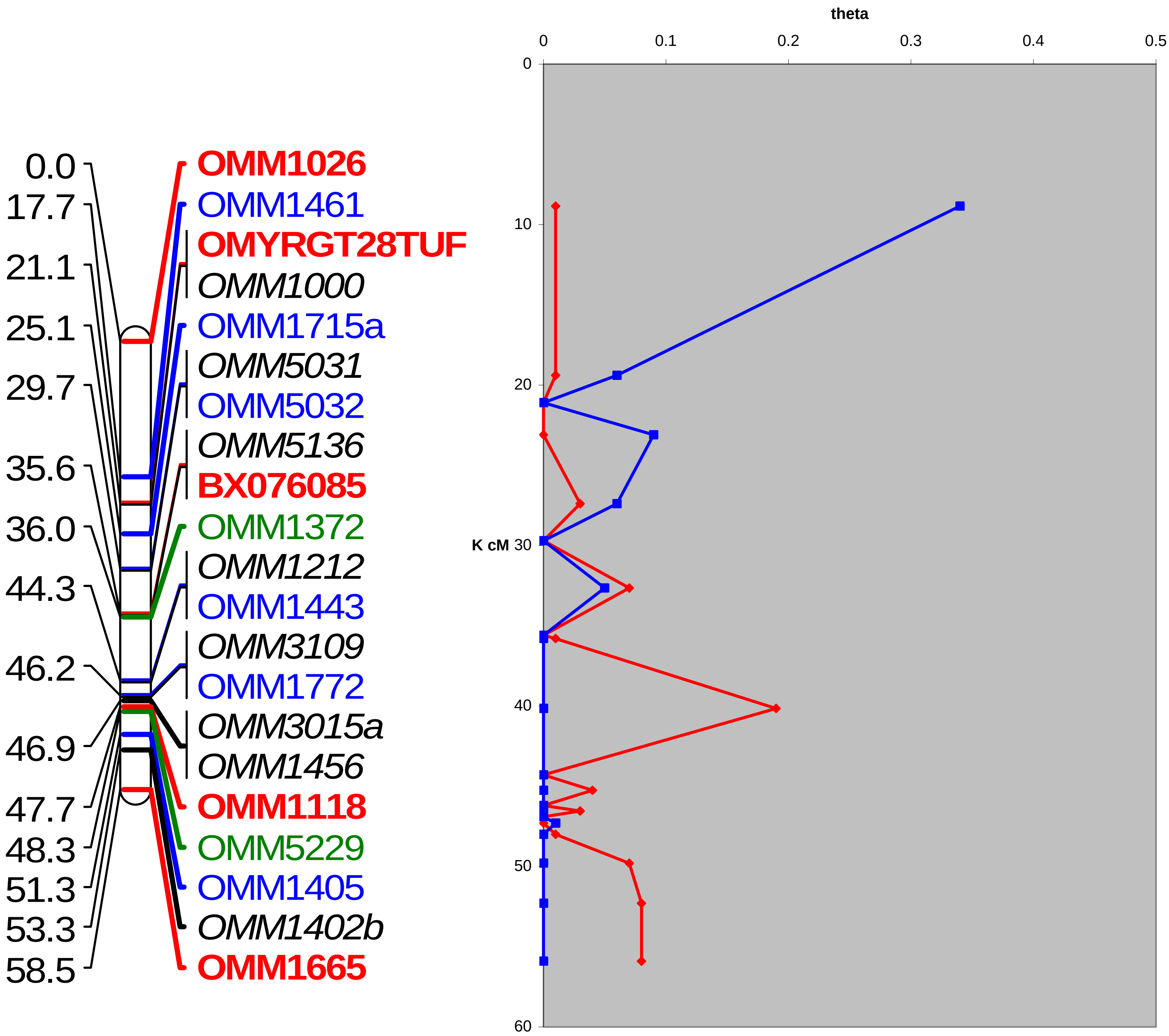

0.0
17.7
21.1
25.1
29.7
35.6
36.0
44.3
46.2
46.9
47.7
48.3
51.3
53.3
58.5
OMM1026
OMM1461
OMYRGT28TUF
OMM1000
OMM1715a
OMM5031
OMM5032
OMM5136
BX076085
OMM1372
OMM1212
OMM1443
OMM3109
OMM1772
OMM3015a
OMM1456
OMM1118
OMM5229
OMM1405
OMM1402b
OMM1665
theta
0
0.1
0.2
0.3
0.4
0.5
K cM
0
10
20
30
40
50
60
